# Artificial microbiome heterogeneity spurs six practical action themes and examples to increase study power-driven reproducibility

**DOI:** 10.1038/s41598-020-60900-y

**Published:** 2020-03-19

**Authors:** Abigail R. Basson, Alexandria LaSalla, Gretchen Lam, Danielle Kulpins, Erika L. Moen, Mark S. Sundrud, Jun Miyoshi, Sanja Ilic, Betty R. Theriault, Fabio Cominelli, Alexander Rodriguez-Palacios

**Affiliations:** 1 Division of Gastroenterology & Liver Diseases, Case Western Reserve University School of Medicine, Cleveland, OH USA; 2 Digestive Health Research Institute, University Hospitals Cleveland Medical Center, Cleveland, OH USA; 3 Department of Biomedical Data Science, Geisel School of Medicine, The Dartmouth Institute for Health Policy and Clinical Practice, Lebanon, NH USA; 4 Department of Immunology and Microbiology, The Scripps Research Institute, Jupiter, FL USA; 5 Department of Gastroenterology and Hepatology, Kyorin University School of Medicine, Tokyo, Japan; 6 Department of Human Sciences and Nutrition, The Ohio State University, Columbus, OH USA; 7 Department of Surgery, University of Chicago, Chicago, IL USA; 8 Mouse Models Core, Silvio O’Conte Cleveland Digestive Diseases Research Core Center, Cleveland, OH USA; 9 Germ-free and Gut Microbiome Core, Digestive Health Research Institute, Case Western Reserve University, Cleveland, OH USA

**Keywords:** Microbiome, Gastrointestinal models, Experimental models of disease

## Abstract

With >70,000 yearly publications using mouse data, mouse models represent the best engrained research system to address numerous biological questions across all fields of science. Concerns of poor study and microbiome reproducibility also abound in the literature. Despite the well-known, negative-effects of data clustering on interpretation and study power, it is unclear why scientists often house >4 mice/cage during experiments, instead of ≤2. We hypothesized that this high animal-cage-density  practice abounds in published literature because more mice/cage could be perceived as a strategy to reduce housing costs. Among other sources of ‘artificial’ confounding, including cyclical oscillations of the ‘dirty-cage/excrement microbiome’, we ranked by priority the heterogeneity of modern husbandry practices/perceptions across three professional organizations that we surveyed in the USA. Data integration (scoping-reviews, professional-surveys, expert-opinion, and ‘implementability-score-statistics’) identified Six-Actionable Recommendation Themes (SART) as a framework to re-launch emerging protocols and intuitive statistical strategies to use/increase study power. ‘Cost-vs-science’ discordance was a major aspect explaining heterogeneity, and scientists’ reluctance to change. With a ‘housing-density cost-calculator-simulator’ and fully-annotated statistical examples/code, this themed-framework streamlines the rapid analysis of cage-clustered-data and promotes the use of ‘study-power-statistics’ to self-monitor the success/reproducibility of basic and translational research. Examples are provided to help scientists document analysis for study power-based sample size estimations using preclinical mouse data to support translational clinical trials, as requested in NIH/similar grants or publications.

## Introduction

According to a U.S. National Science Foundation subcommittee on science replicability, “reproducibility refers to the ability of a researcher to duplicate the results of a prior study using the same materials as were used by the original investigator. That is, a second researcher might use the same raw data to build the same analysis files and implement the same statistical analysis in an attempt to yield the same results”^1^. More recently, reproducibility as a scientific concept has been proposed to be divided into three types: methods reproducibility, results reproducibility, and inferential reproducibility. While these terms are applied predominantly to the biomedical field, they are not without utility across other scientific fields, each of which are governed by their own internalized needs and criteria for “proof”^2,3^. With 73,363 PubMed publications using ‘mice’ in 2018, laboratory mice represent a critical component to understanding human biology in a variety of fields, from inflammatory bowel diseases, neurology, and cancer, to microbiome and nutrition. In the current era of microbiome research, multiple factors are becoming evident as sources for confounding. Integrating microbiome science into animal research necessitates that experiments control for confounding derived from emerging artificial factors, especially the ‘cage microbiome’^4–8^, which we recently discovered causes ‘cyclical microbiome bias’ due to the periodic accumulation of excrements in mouse cages^4^. Understanding the factors that contribute to research heterogeneity will address this need. Primary causes of artificial analytical heterogeneity and low study power include putting many mice into one cage, having insufficient cages per group, and using incorrect statistical methods assuming that groups of mice in a cage are independent, and not cage-clustered data.

In statistics and science, heterogeneity is a concept that describes the uniformity and variability of an organism, a surface, or the distribution of data. Sources of study heterogeneity can be natural or artificial. Artificial heterogeneity refers to study variance introduced by humans or anthropological factors, including animal husbandry and the ‘cage microbiome’, which non-uniformly affect mouse biology. Fundamental to hypothesis testing, data heterogeneity determines which statistical methods are needed to decisively quantify if two independent naturally-heterogeneous groups, truly differ. To appropriately select statistics controlling for cage-clustered data, scientists must be aware of study details, namely, which data points belong to which mice and respective cages in a dataset or published figure. Unfortunately, these details are often omitted during analysis and in publications, and misconceptions on heterogeneity, husbandry and analysis may exist among leading research organizations.

To exemplify that scientists are under pressure and need recommendations to prevent bias and improve animal research quality and reproducibility, the National Institutes of Health (NIH), a major federal funding institution in the US implemented a mandate on ‘Rigor and Reproducibility’ in 2014^9–11^. The mandate assures funding is constrained unless researchers prove that they consistently yield reproducible results. Our report seeks to illustrate concepts on study power and intra-class correlation among mice in a cage to support a framework based on Six Actionable Recommendation Themes (SART) to increase study reproducibility.

Concerning study power, two concepts of expected validity exist: internal and external validity. Both refer to the statistical expectation that results from a given study are true, reproducible, and not by random chance if a study is repeated locally (internal), or in another setting (external validity)^12–14^. Intrinsically, experiments have high internal validity if appropriate statistics and power are applied, and if data clusters and confounders are avoided. Studies with experiments in different settings (microbiota, mouse lines) are more likely replicable; but experimental reproducibility requires appropriate power. Validity thus depends on the study power, which is the probability of not making a type II error (fail to reject false null hypotheses in favor of true alternatives). Power is a statistical measure from 0 to 1, with 1 indicating highly-powered studies. While power 0.5 yields statistically haphazard results (‘tossing a coin’), powers >0.8 indicate optimal chance for replication. Power increases with large sample sizes (more mice), but decreases with clustering of animals in cages by introducing a ‘cage effect’, and intra-class correlation coefficient (ICC) complexity to the analysis of cage-clustered data. The negative impact of cage clustering is maximum when all mice of a study group are housed in one cage because it is impossible to differentiate ‘real’ from ‘confounding cage effects’. The negative impact of clustering is reduced when more cages, with *fewer mice per cage*, are used per group (*‘less mice-per-cage is more’*).

Despite the 5-year-old NIH mandate, the public and federal perception on mouse research reproducibility is often negative^10,15^. However, to our knowledge, there are no scientific studies (i) confirming that research reproducibility is an ongoing issue, (ii) defining what role perceptions and academic husbandry practices play on reproducibility, or (iii) predicting the implementability of potential solutions to increase study power, if proposed. To refine our understanding on research heterogeneity, study power and reproducibility, our study objectives were to, (i) verify research methods heterogeneity in current literature, (ii) quantify current perceptions on mouse husbandry and microbiome using a survey, (iii) identify potential areas of solution using a Delphi-based strategy, and (iv) to quantify the potential implementability of an evidence-based framework of six recommendation themes to cost-effectively increase study power using a grading scale based on perceived clarity, benefit and recommendability.

As an accompanying practical set of tools, we also created (i) a simple housing density cost calculator in Excel that can be used by scientists to determine whether less animals per cage, or more cages per experimental group suit research budgets, and (ii) provide graphical examples and a fully annotated statistical code to compute and report analysis of cage-clustered data, and power, for both single- and clustered-caged mice. Post-hoc study power calculations were deemed cumbersome and non-informative in the past^16^, but more sophisticated user-friendly software now provides emerging methods to compute such important statistics^17^, which we provide for investigators to infer and objectively self-monitor power and reproducibility across mouse research at large.

## Results

### Husbandry heterogeneity and cage-cluster effects are pervasive in current literature

**Figure 1:**
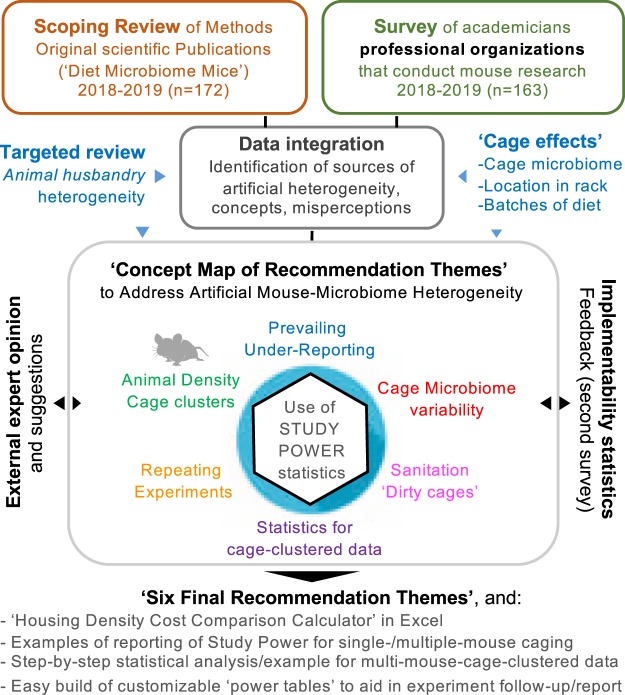
Study design to understand artificial heterogeneity in mouse microbiome research and a ‘Concept Map of Six Actionable Recommendation Themes’ to facilitate, increase, and promote the use of study power statistics to self-monitor the quality of research.

**Figure 2:**
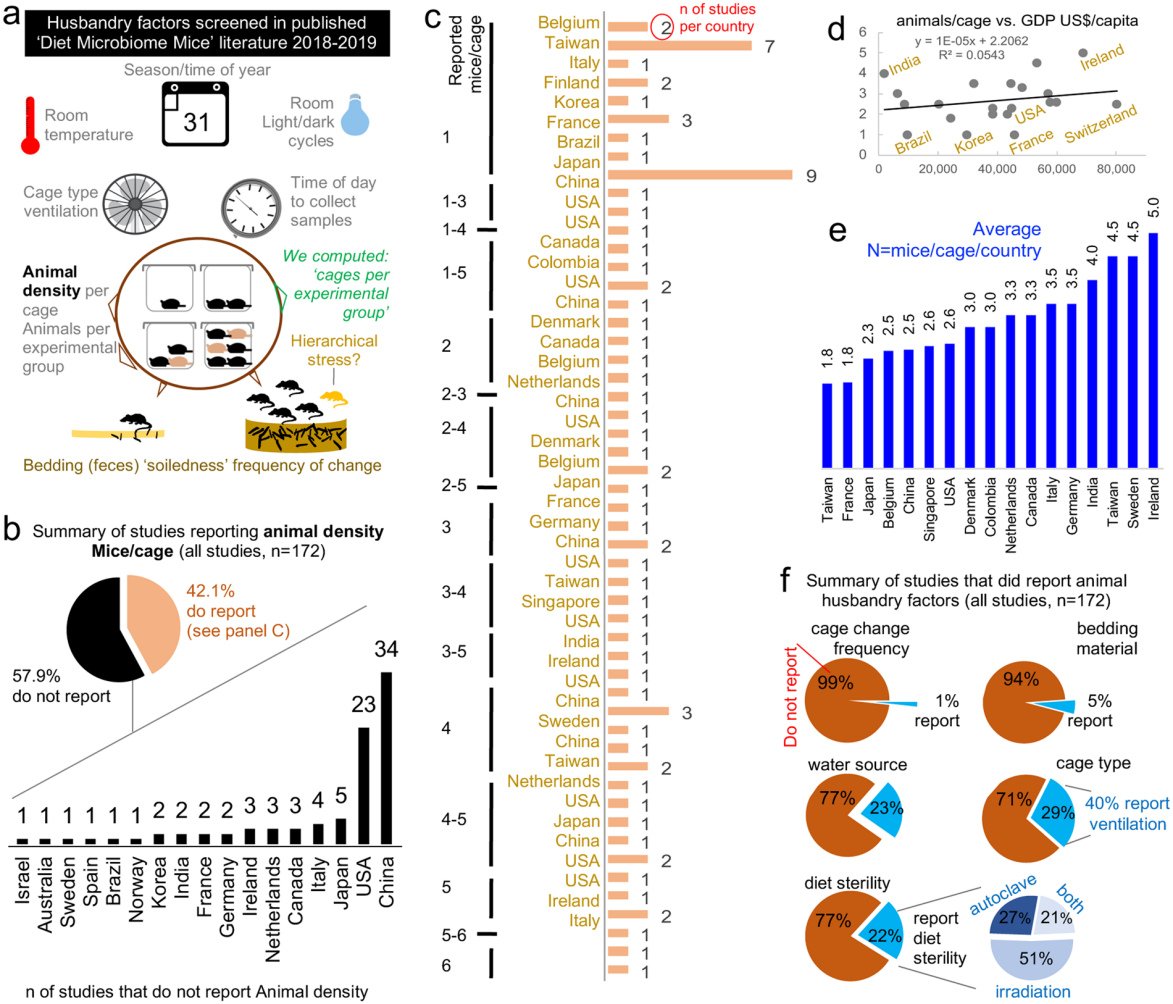
Literature on ‘diet, gut microbiome & mice’ illustrates ongoing animal density problematics. Published methodologies illustrate variability in husbandry and inconsistent animal density across studies as a major source of cluster-confounding. (**a**) Schematic representation of factors screened from the methods and results section in peer-reviewed publications. (**b**) Distribution of studies that did and did not report animal density. Pie chart shows that most studies (58%) do not report how many animals were housed per cage. (**c**) Ranking shows MxCg as reported by the number of studies per country based on the number of studies reporting animal density (78 of 172 reported). Note that 15% of reviewed studies reported exclusively housing 1 MxCg (27 of 172 total studies). (**d**) Correlation between number of MxCg reported by reviewed studies and their representative GDP US$/capita. Note that the country’s GDP does not correlate with number of MxCg suggesting experimental animal density practices are not related to wealth of a country. (**e**) Average MxCg used in experiments represented by country. (**f**) Summary of studies that reported cage change/sanitation frequency, bedding material and diet sterility (including method for diet sterilization; autoclaving, irradiation & dose used). Note that more studies reported ‘cage type’ (e.g., plastic flexible film, metal wired, Plexiglas, etc.) than those which reported ‘sterility of diet’ (25% vs 21%). Only one study reported ‘time of fecal collection’ (see complementary data in Supplementary Fig. 1).

To identify husbandry factors capable of influencing gut microbiome and study reproducibility, especially mice per cage (MxCg) and mice per group (MxGr), we reviewed 172 recent studies selected from PubMed searching ‘diet-microbiome-mice’ (Fig. 1). From 865 articles published over the past 10 years, 93% were published in the last five years (Supplementary Materials). Of concern, most studies failed to report in sufficient detail aspects of animal husbandry (*e.g*., cage density/sanitation frequency, diet sterility) making the study of cage-effects and confounding challenging to assess (Fig. 2, Supplementary Fig. 1). While 30% of the studies originated in China and USA (n = 52), it is remarkable that almost 60% of studies across all countries failed to report animal density (*i.e*., MxCg). Of the 72 studies that reported density, 30% (22/72) have highly cage-clustered data; reporting experiments with 5 MxCg. Slightly encouraging, 18% of studies housed mice at lower densities of ≤2 MxCg, which is ideal because it increases study power by decreasing cage effects (Fig. 2a–c). Although low animal density could be perceived as an expensive practice, density practices did not correlate with gross domestic product (GDP; yearly US$/capita) implying that national wealth is not a driving factor for housing mice individually during experiments. Irrespective of wealth, it was reassuring to identify scientists who publish studies stating that they exclusively housed mice individually in Belgium, Taiwan, Italy, Finland, Korea, France, Brazil and Japan^18–48^ (Fig. 2d,e).

Several husbandry aspects contribute to cage-cage variations and cause cage effects (see Supplementary Tables 1 and 2). Therefore, it is difficult to substantiate whether the significant effects identified in any given study, where all mice in a group were housed in one single cage (decreasing study power), were truthfully due to the experimental intervention and not from the random distribution of cage effects in a laboratory (Fig. 2f). To quantify the potential for ‘cage effect confounding’, we used the ‘total number of cages per group’ (TCgxGr) as a quantitative estimate (see Methods) to determine the prevalence of studies that conducted experiments using only a few cages per group. Estimates indicate that studies used on average 4.4 ± 3.2 TCgxGr (notice large SD), of which 39% (28/72) generated data derived from only 1–2 TCgxGr (Supplementary Fig. 1).

Given that cage clusters decrease study power^49–51^, experiments conducted with low animal density, ideally one MxCg, and the reporting of TCgxGr deserves to be highlighted as an exemplary habit. Despite available reporting guidelines^52^, data illustrates that inadequate reporting of methodological details in published literature continues in 2019, diminishing the ability to replicate studies. To complement guidelines, we propose to consider using a standard verbatim paragraph-style format to unify reporting and facilitate future meta-analyses (see below Recommendation Theme on ‘Reporting’).

### Expertise differences across scientific organizations surveyed

**Figure 3:**
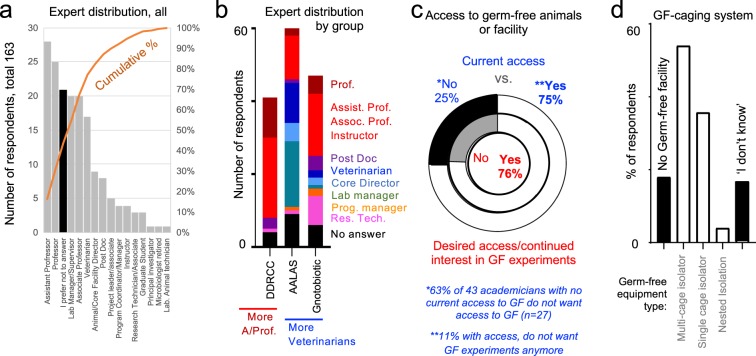
Demographics of surveyed professionals on ‘animal husbandry in microbiome research’. (**a**) Pooled distribution of job descriptions categorized based on information provided by all respondents. (**b**) Distribution of job descriptions by the three largest groups of participants. Notice that the DDRCC group has the largest proportion of faculty (from instructors to full professors) participating in the survey, but all groups were composed of academicians with comparable job descriptions. More veterinarians and project leaders were observed in the AALAS and Gnotobiotic listserv groups. (**c**) Distribution of participants who reported having current access to GF animals or facilities (outer pie circle chart) and that would like to have access, or continue working with, GF animals/facilities (inner circle chart). Notice that the majority of participants are expected to have high levels of expertise and understanding of GF mouse facilities, husbandry, and microbiome knowledge. ‘*’ and ‘**’ indicate subgroups who would like (or not) to change their current GF research trends. (**d**) Distribution of respondents who did or did not know about the presence of GF facilities in their institution, and the types of caging system used. This question contextualizes the knowledge of respondents in terms of GF equipment/systems.

To further advance our understanding of husbandry heterogeneity, we applied an online survey to academicians (Supplementary Fig. 2). After contacting over 2000 professionals, a total of 166 participants started the online survey. One-hundred and sixty-three (97%) surveys were completed and used for analysis. The majority of respondents were from USA (133; 81%, 95%CI = 74.3, 87.6) and participants reflected individuals with leading roles in science (Assistant Professors, Professors, Veterinarians) within the DDRCC, AALAS and GNOTOBIOTIC organizations (see Methods). The GNOTOBIOTIC respondent set had a smaller number of faculty/veterinary directors or managers (vs. Postdocs) compared to the DDRCC group (p = 0.087, 61.4% vs. 78.8%, Odds ratio [OR] = 2.15 95% CI = 0.82, 5.7) but included slightly more participants with access to germ-free (GF) animals compared to DDRCC (p = 0.083, 95.5% vs. 84.6%, OR = 3.82, 95%CI = 0.69, 38.5, Fig. 3a,b). Multi- and single-cage GF isolators (used as a proxy for state-of-the-art equipment and knowledge) were most frequently used as a GF-caging system among those with GF facility access. Collectively, demographic analysis indicates that although statistically different, all groups had comparable levels of expertise, access to state-of-the-art facilities and knowledge (note p-values and wide 95%CIs; see Fig. 3c,d) which is important to inferring that the perceptions acquired herein are relevant to current research.

### Scientific organizations rank similarly 15 husbandry factors that affect the mouse microbiome

To determine whether differences in knowledge/practices or perceptions on animal husbandry exist due to the professional nature of each organization, we asked participants to rank, from 1 to 5 (least to most important), how important each of 15 husbandry factors contribute to variability in mouse research (“*Rank how important you believe each of the following 15 aspects contribute to microbiome research variability)*”. Using ‘*diet composition*’ as a positive control (as diet affects gut microbes), we found that all groups of professionals ranked each parameter similarly (mean of ranks for all participants across factors, Kruskal-Wallis p > 0.05).

**Figure 4:**
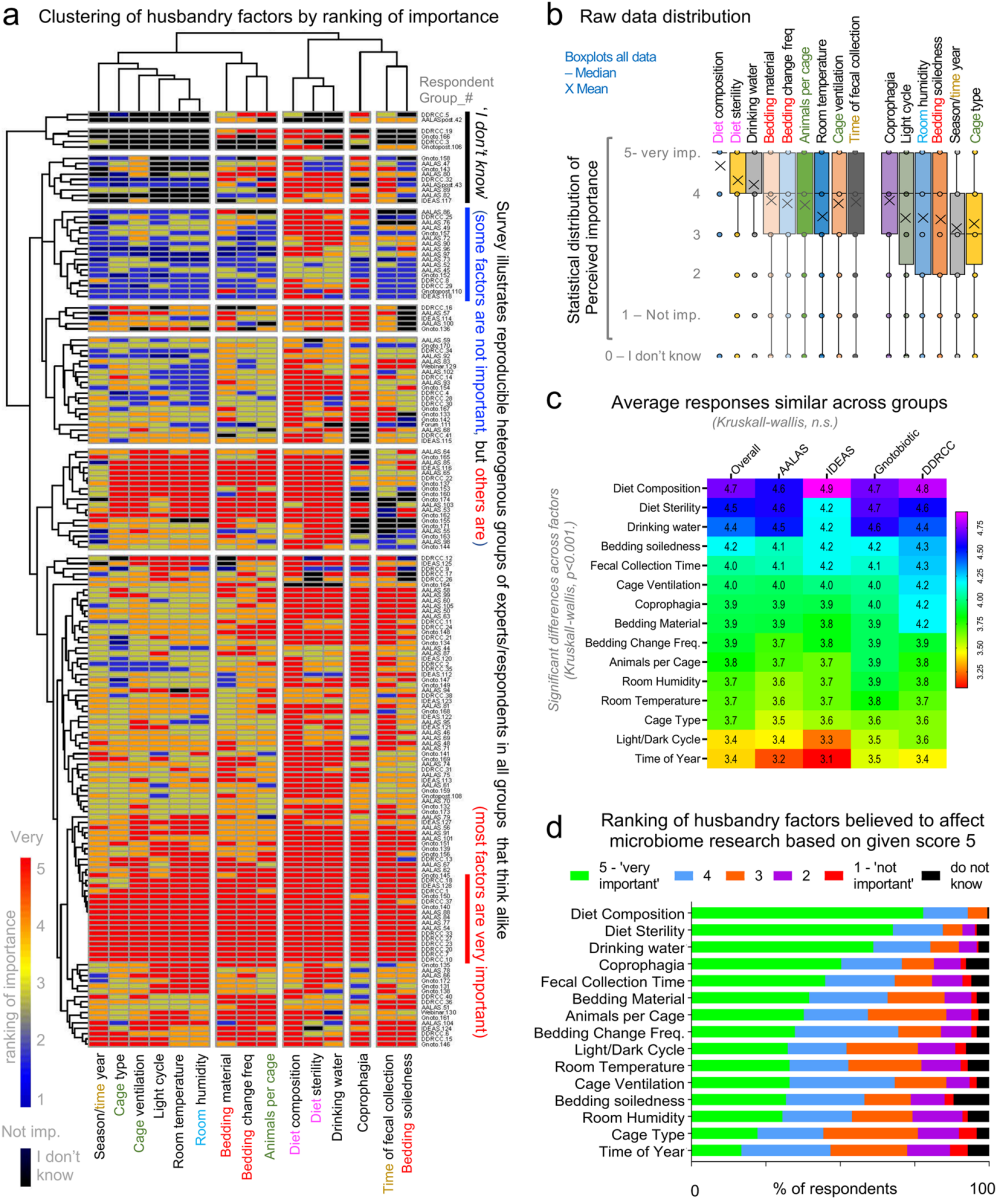
Ranking of 15 factors believed to cause microbiome research variability is reproducible. (**a**) Heat map shows respondent perceptions on the importance of various animal husbandry factors in microbiome research variability. The heterogeneity across respondent perceptions illustrates that individual thinking is not related to institutional affiliation. (**b**) Boxplots show raw data ranking distribution of respondent perceptions on the importance of various animal husbandry practices. (**c**) Heat map shows the overall ranking of variables according to institution. (**d**) Stacked bar graphs show overall ranking of variables. Note that diet composition, sterility and drinking water were identified by >50% of individuals as ‘very important’ contributors to microbiome research. Note the discordance between coprophagia (ranked 4^th^) to that of bedding soiledness (‘dirtiness’) and the importance of cage change frequency.

Except for *‘diet composition’*, ranked 1^st^ as ‘very important’ by the majority of respondents (>75%), there was marked heterogeneity in response patterns at the individual level (Fig. 4a). Importantly, perceptions of individuals did not cluster within their professional affiliation, suggesting that the organizations surveyed ‘think’ alike. Instead, we identified ‘patterns of beliefs/perception’ in academia that reflect ‘types of individuals’, with a given set of research practices in mind (beliefs), that differs from their peers within their organization (Fig. 4a–c). For example, although ‘*coprophagia’* ranked 4^th^ overall as a ‘very important’ factor to microbiome variability, fewer than 40% of participants ranked *‘number of animals per cage’* (ranked 8^th^) and *‘cage change frequency’* (ranked 9^th^) as aspects ‘very important’, even though coprophagia contributes to microbiome confounding depending on the extent of *‘cage bedding soiledness’* (ranked 12^th^), which depends on *‘number of animals per cage’* and *‘cage change frequency’*.

In the studies reviewed, aspects deemed ‘very important’ by survey respondents were not always reported, while ‘less important’ factors were frequently reported. This discordant pattern of thinking-reporting was further illustrated by individual perceptions on ‘*bedding type’* (*e.g*., corncob vs. non-edible wood shavings), ‘*cage ventilation’* type, ‘*room temperature’* and *‘room humidity*’, all of which contribute to cyclical bedding microbial overgrowth (which selects for aerobic microbes in cage bedding) and thus cage-cage microbiome variability^4,5,51^. Beliefs agreement was identified between ‘*diet composition’, ‘diet sterility’* and *‘water source’* (top 3 ranked factors) illustrating that dietary intake is perceived as a collective of all aspects consumed orally, including the microbial content of diet (Fig. 4d). Most respondents do not think *‘cage type’* (ranked 14^th^) is important. The majority of reviewed studies (Fig. 2f), however, reported cage type in their methods, while the ‘very important’ aspect of *‘diet sterility’* was described in only 22% of studies reviewed. Of concern, the *‘time of year/season’* was the least important aspect believed to influence the microbiome (ranked 15^th^); however, we have shown that cross-sectional metagenome experiments conducted in separate seasons produce contrasting results when assessing the role of *Helicobacter* spp. in spontaneous Crohn’s disease-like ileitis in mice^6^, implying that repeating experiments across seasons may yield unreproducible results over time.

As a recommendation, repeating experiments to build composite datasets, which often occurs across seasons, should be conducted with caution unless we understand the effect of season on the microbiome and animal physiology (see Recommendation Theme on ‘Repeating Experiments’).

### Diet-dwelling microbes and homogenizing cage microbiome variability before experiments

With sub-sterilizing radiation protocols, diets have variable microbial composition even within the same batch^4,5,53^. Survey questions interrogated basic knowledge relevant to irradiation and the degree of diet sterility. When asked whether standard irradiated commercial diets for mice were sterile, 67% answered that such diets were *‘sterile’*. Although diet sterility depends on the irradiation dose, in the case of commercial diets, companies employ a single, standard dose, insufficient to achieve GF-grade sterility. Of note, no studies reviewed reported irradiation dose when reporting *diet sterility*. Thus, unless certified as sterile, diets used during mice rearing and experiments expectedly contain potentially confounding microbes, primarily spore-formers and gamma-radiation resistant bacteria and fungi^54^. The random distribution of diet-dwelling microbes, bedding-dependent microbial overgrowth and other cage effect factors are sources of microbiome divergence^55^ and bias that accumulate across cages as animals are reared and aged before, or during experimentation.

**Figure 5:**
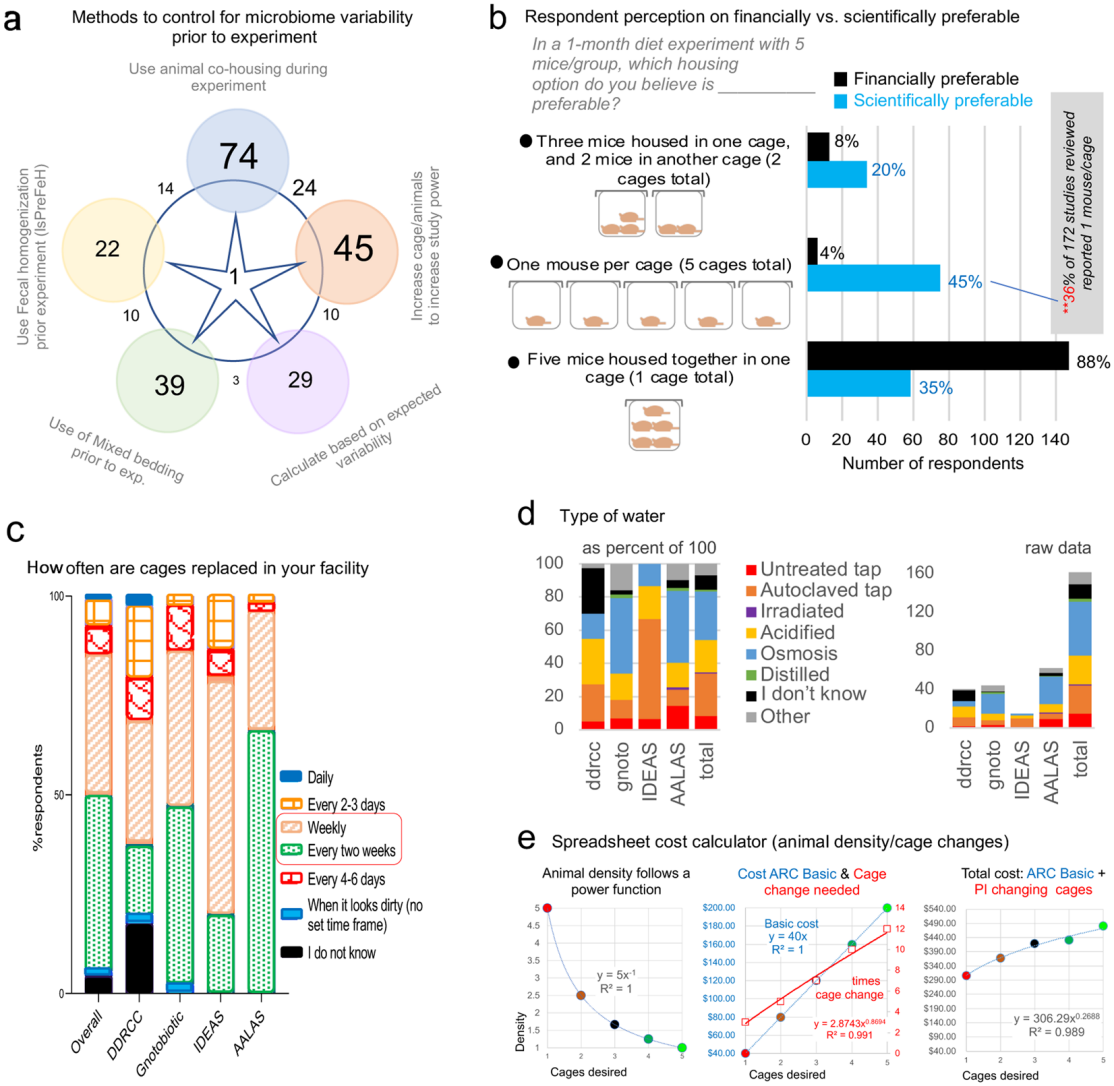
Survey responses for animal husbandry practices and cost. (**a**) Venn diagram (n of respondents) on ‘popularity’ of various methods used to control cage-cage microbiome variability prior to the experiment. Note ‘fecal homogenization protocol’ compared to others. (**b**) Perception contrast between the ‘financial’ and the ‘scientific’ preference when asked what animal density was preferable for a 1-month dietary experiment. Of interest, 88% and 35% of the survey respondents believe that 5 MxCg (i.e. group housing of mice) is financially and scientifically preferable than housing fewer animals per cage. (**c**) Stacked bar plots show ‘cage change frequency’. Most facilities change cages weekly or every 2 weeks. (**d**) ‘Water-type’ in facilities (8.6% ‘did not know’) shown as the raw data (right) and as a percentage of 100 (left). Note wide array of water sources, including untreated tap, autoclaved tap, acidified tap and reverse osmosis, all of which affect the gut microbiota^86^. (**e**) Cost analysis example using a customizable spreadsheet calculator (Supplementary File 1). Notice the power function correlation between ‘number of cages desired’ in a study and ‘animal density’ with the linear costs of husbandry due to payment of ‘basic costs’ in an Animal Resource Center (ARC) and the presumed costs of cage handling by a technician paid by the Principal Investigator (PI).

Since there is no consensus on one single approach to control for cage-cage microbiome variability before using mice in experiments, we surveyed which methods are used by scientists^55–58^. Despite evidence that co-housed mice have varying microbiome patterns^59,60^ and the recent evidence of cyclical bedding-dependent bias^4^, the most popular combination of methods used to control for cage microbiome variability was *‘cohousing’*, ‘*use of mixed bedding’* and *‘increasing the number of animals per cage’* (Fig. 5a). The least frequently used method was *‘fecal homogenization’* (animals exposed to a composite of feces harvested from all mice), yet this method is arguably the simplest and most effective in homogenizing cage microbiome variability (see Recommendation Themes on ‘Cage-cage microbiome variability BEFORE experiments’ and ‘Dirty cages and time-of-sampling DURING experiments’).

### Clusters and scientific-financial discordance when housing five mice in a study of five mice

To interrogate whether cost is a contributing factor to animal housing density practices, we posed two identical multiple-choice questions that differed only by the assumption of financial vs. scientific preference. The first survey question asked, “*In a 1-month diet experiment with 5 mice/group, which housing option do you believe is FINANCIALLY preferable?* while the second question replaced the capitalized word ‘*FINANCIALLY’* with *‘SCIENTIFICALLY’*. The three possible answers were, using ‘5 cages’, ‘2 cages’, or ‘1 cage’. The majority of participants believe it is both scientifically (54%) and financially (95.7%) preferable to maintain cages with higher animal density (2–3 or 5 MxCg), which, of concern, introduces cage cluster effects^61^. Thus, studies with 5 mice are underpowered as they consist of only 1–2 cages; commonly seen in studies reviewed. Intriguingly, while 45% (95%CI = 37.3, 52.6) of survey respondents think that it is more scientifically appropriate to have 1 MxCg, the same individuals do not think that this practice is economically feasible (Fig. 5b), which reflects current literature where only 15% (27 of 172 total, 95%CI = 9.6, 20.3) of studies reported exclusively housing 1 MxCg (see Fig. 2c).

Considering that the majority of respondents’ facilities implement weekly or every 2 weeks ‘*cage change’* protocols, with a wide array of drinking water sources across facilities (Fig. 5c), our data suggests that cage change/sanitation (via ‘cage microbiome’) (Fig. 5d), and animal density could contribute greatly to artificial heterogeneity in mouse research.

**Figure 6:**
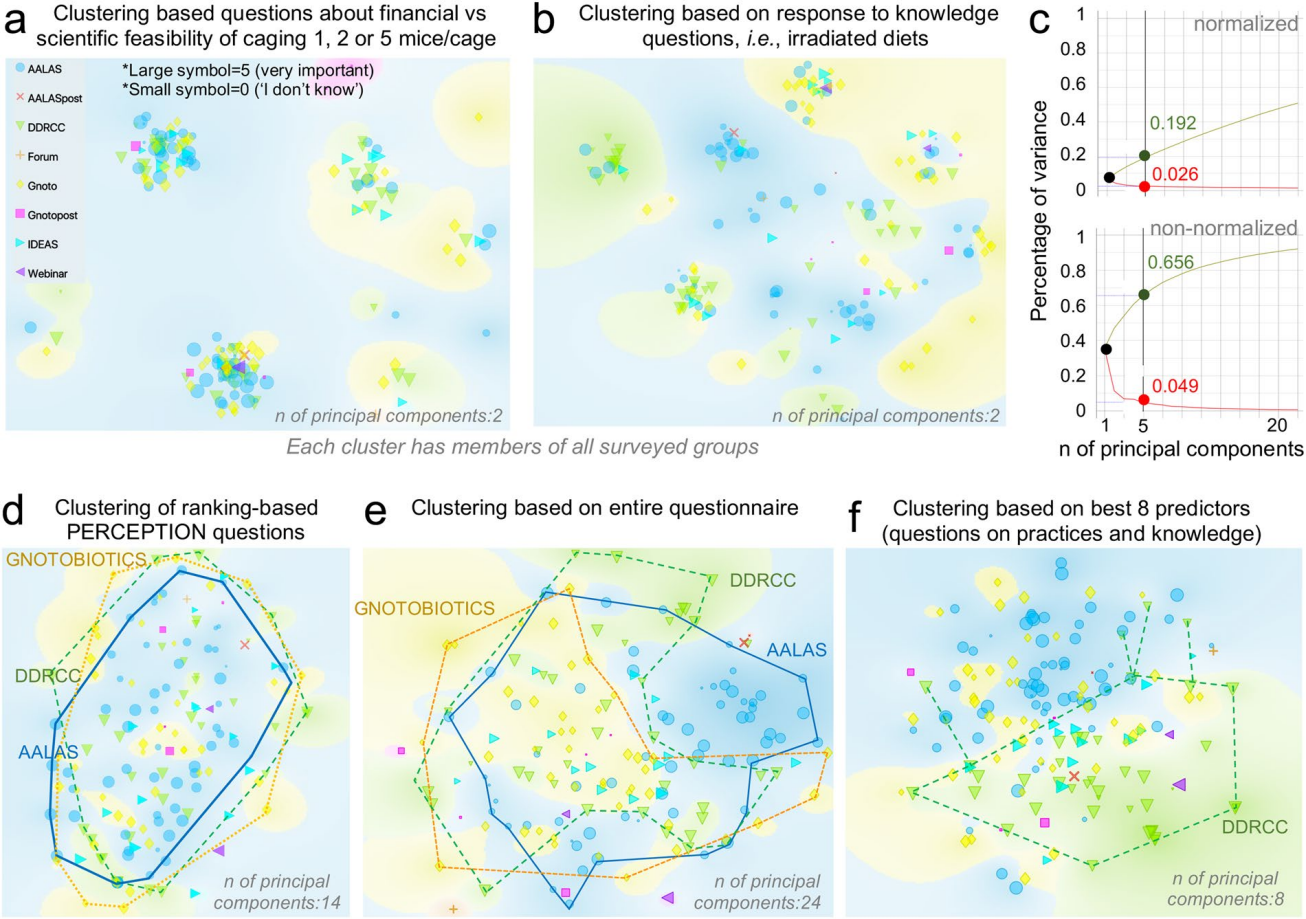
Beliefs on ‘husbandry and microbiome research variability’ are similar, but professional organizations differ in response to questions on practices and knowledge. Normalized principal component analysis of survey respondent data. Superscript asterisks: large or small symbols depict the individual response of each participant when asked how important ‘animal density’ was as a factor in influencing the gut microbiome. (**a**) Clustering-based questions about financial vs. scientific feasibility of caging 1, 2 or 5 MxCg. Notice that each cluster (type of response patterns) contains individuals from all professional groups, i.e., AALAS. (**b**) Clustering-based knowledge questions, i.e., irradiated diets. Notice the same pattern as in panel A, suggesting that response heterogeneity is not due to group. (**c**) Normalized and non-normalized percentage of variance in entire data set explained by the maximum number of components (questions; n = 24) using “animal density” as outcome for prediction (which cannot be achieved as large and small symbols occur throughout plot). (**d)** Cloud representation of collective influence of the 15 questions to predict group separation. **(e**) Clustering based on 15 ranking-based PERCEPTION questions + 11 Knowledge, Financial vs. Scientific feasibility, access to facilities and practices. Although clusters of individuals collectively think very similarly and slightly different than the rest,  analyses indicate that the different clustering for certain areas in the plot is due to differences in answers related to ‘type of facilities’, or practices that are more common among certain groups of professionals. (**f**) Best achievable clustering of individuals based on relief F scores to predict animal density shows surveys from different groups are distinct.

To address concerns of cost regarding the number of MxCg in context to ‘*cage change frequency’*, we developed an Excel spreadsheet ‘Housing Density Cost Comparison Calculator’. Graphical cost-effectiveness analysis illustrates that a higher number of MxCg requires more frequent cage changes (Fig. 5e, available as https://figshare.com/s/377fa429bd8cc405fc1b). Overall, costs increase when comparing 5 vs. 1 MxCg linearly over a continuum of cage cluster possibilities, therefore conducting highly clustered underpowered studies is not necessarily cheaper. When considering response patterns regarding financial vs. scientific feasibility of animal housing density, we show that the heterogeneity in respondents’ perceptions is not attributed to institution but instead to professional organization (Fig. 6a–f).

Although scientists could argue that statistical methods exist to control for clustering^61^, our analysis of literature indicates that scientists do not implement cluster-statistics. Since cluster-statistics are not trivial to implement (*e.g*., R Statistical Package ‘clusterPower’^62^), we provide technical guidelines on how to account for unbalanced MxCg designs, ICC and low sample size using clustered-data statistics (see Recommendation Themes 5–6 on ‘Animal density, clusters, ICC, and power’).

### Identification of potential areas of solution (themes) using a Delphi-based strategy

**Figure 7:**
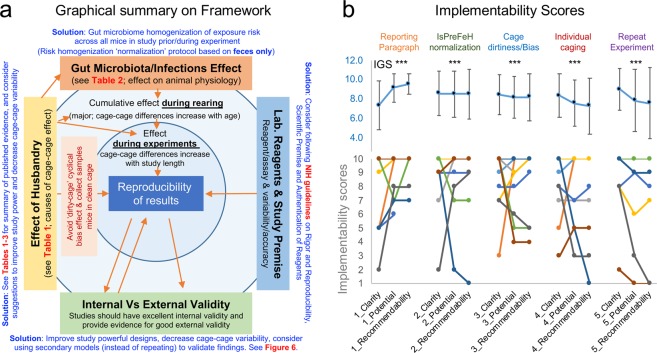
Implementability of recommendation theme framework. (**a**) Framework integrating NIH guidelines, and our recommendations. **(b**) Implementability grades scores (IGS) for each recommendation. Asterisks indicate IGS were statistically higher than random simulations (see statistics details in-text). Line plot connects individual grades. Notice that people who disagree with sentence clarity tend to disagree interpreting the potential benefits. High mean grades indicate high potential for implementability.

To enable a practical solution to the aspects of husbandry and reporting heterogeneity, highlighted in the results section above and described below as practical ‘action themes’, a multi-theme actionable framework was constructed for statistical validation of implementability (Figs. 1 and 7a). To statistically determine if the ‘action themes’ were (i) clearly drafted as a sentence (*sentence clarity*), (ii) had the potential benefit to improve power and reproducibility (*potential benefit*), and (iii) were deemed appropriate for readers to recommend to others (*would you recommend it?*), we asked active academicians and scientists conducting research to grade each recommendation and provide comments to create an ‘implementability grade metric’ (Supplementary Table 3).

To objectively quantify whether the obtained implementability grades were significantly different from random responses, we compared the distribution of grades to that of a random generator of 30 numbers, from 1–10. A grade of 1 indicates poor, while a grade 10 means outstanding. Of great practical value for the multi-theme framework proposed, analysis indicated that, collectively, all recommendations are very likely to be implemented by scientists (mean grade, 8.02 ± 1.4 vs. random grade 5.0, n = 20, t-test p < 0.001; Fig. 7b).

The wording of the post-expert-assessed action theme recommendations here described as the final result of the Delphi-based study (underlined with ‘*quotation marks and italics*’) reflect the improved version of the expert-graded sentences and comments received during the grading phase. See examples of the representative comments provided by the participants in Supplementary Table 4, and a synthesis of the husbandry concepts used to integrate the survey data with peer-reviewed literature supporting the framework in Supplementary Table 5. The implementability statistics, rationale (extended version in Supplementary Table 6), and goals for each suggested recommendation theme are described below.

#### Action theme 1 on ‘Reporting of diet and husbandry factors’

*‘Use of a paragraph-style template to report detailed diet and husbandry factors consistently and reproducibly (e.g., macronutrient, diet sterility), publishable as accompanying “Supplementary Materials”*. See an example of a reporting template in Supplementary Table 7. The goal of this theme would be to minimize reporting with insufficient detail or details that are open to interpretation, yet still suffice standard reporting checklists/guidelines^52^. The expert-prediction for implementation is significantly high (grade, 8.7 ± 1.2 with 99.5% probability of being significantly higher than random in 96.7% [n = 29] of t-test analysis conducted for 30 simulations with 30 random numbers, mean t-test p = 0.005 ± 0.012). Note that ‘text-recycling’ is currently allowed (when clearly justified) based on current code of ethics in scientific publishing^63–65^.

#### Action theme 2 on ‘Cage-cage microbiome variability BEFORE mouse experiments’

*‘Use of a fecal matter-based microbiome normalization protocol (e.g., by orally administering a homogenous pool of feces from a group of mice intended for experimentation to all the mice at baseline prior to starting the study) to homogenize the microbial exposure risk across all mice intended for an experiment, and thus reduce the cage-cage microbiome variability that naturally occurs as animals age during intensive production of animals for research and experiments*’. The goal would be to normalize microbiome variability that accumulates across cages over the lifespan of mice before experiments. The expert-prediction for implementation is significantly high (grade, 8.5 ± 0.04; 98.25% probability of higher score vs. random; significant in 86.6% of simulations, t-test p = 0.018 ± 0.03). Described in 2014 as ‘Inter-subject Pre-experimental Fecal Microbiota homogenization’ (IsPreFeH)^57^, this revised microbiome ‘normalization’ protocol, which excludes use of soiled bedding material, in combination with a reproducible protocol for oral gavage of microorganisms^66^, is a scalable solution, which has served to identify the role of the microbiome in the treatment of experimental Crohn’s disease in mice using anti-IL1-alpha antibody neutralization^67^.

#### Action theme 3 on ‘“Dirty cages” and time of sampling DURING experiments’

*‘Prevent the uncontrolled accumulation of animal excrements in the cage, (i) house a homogeneous number of animals per cage (ideally at low density, 1 mouse/cage), (ii) adjust frequency of cage sanitation based on animal density, and (iii) collect samples 1–2 days after mice have been in clean bedding/cages, because coprophagia and ‘dirty cages’ affect the mouse physiology and microbiota’*. The goal would be to minimize the uncontrolled permanent contact of mice with their (decomposing) feces. The expert-prediction for implementation is significantly high (grade, 8.3 ± 0.15; 98.7% of probability of higher vs. random; significant in 96.6% of simulations, t-test p = 0.014 ± 0.024). Given that coprophagia (not relevant to humans) and excrements in cages may cause bedding-dependent cyclical microbiome bias^4^, frequent cage replacements (increases with animal density^4^, Supplementary Fig. 3), studying/sampling mice in clean cages and/or the use of slatted floors^68^ deserve emphasis.

#### Action theme 4 on ‘Repeating experiments in different seasons’

*‘Plan and execute statistically powerful designs and do not repeat underpowered (cage clustered, low sample size) experiments in different seasons (because several unforeseen factors affecting animal husbandry are challenging to detect and control for in diet and personnel)’*. The goal would be to control for the variable effect of season on study reporting and heterogeneity using well-powered designs. The expert-prediction for implementation is significantly high (grade, 8.1 ± 0.76; 96% probability of higher score vs. random; significant in 76.6% of simulations, t-test p = 0.04 ± 0.062). We acknowledge that at times replication is desirable, and also that ‘poor breeding colonies’ often yield insufficient mice to perform final experiments. In this context, it is advisable to store fresh-frozen feces anaerobically (−80 °C with/without cryoprotectants; 7%-DMSO, 10%-glycerol) from initial experimental mice for the colonization of newly available mice, and to store sufficient vacuum-packed diet (−20 °C) and supplies to last across experiments.

#### Action theme 5 on ‘Animal density, clusters, and study power’

**Figure 8:**
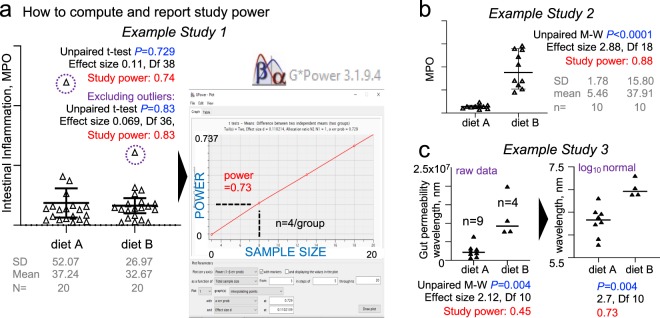
Graphical examples of rapid ‘study power’ calculations and reporting of individually-caged mouse data. (**a**) Example of study power calculation & graphical reporting (post-hoc means after study completion, all datasets are real unpublished data). Intestinal inflammation in mice from two groups housed individually after pre-experimental cage microbiome normalization using IsPreFeH (fresh feces only; no bedding material). Post-test plot analysis (inset, software screenshot of power vs. sample size) shows that in this case, only 4 mice would be needed. Notice p-value and power increase after excluding outliers (dashed circles, N = 19). (**b**) Power analysis for two groups with different variance (diet A, narrow SD; diet B, wide SD). Fecal MPO test following a diet intervention illustrates that for this diet, a sample size of 10 is sufficient to achieve a well-powered study despite large variance in diet B. (**c**) Example of importance of data normalization (e.g., from raw small changes in millions, 10^7^, to a log scale) in post hoc power analysis. Fluorescence intensity units in a test after intervention caused early mortality in diet B. Although the p-value does not change, normalized data (smooth edges of datasets) increases study power as it fulfills assumptions of t-tests normality. Since all mice were individually caged, the dataset quality and the early mortality are not due to, or are confounded by cage effects. Therefore, despite the small sample size (n = 9 vs. 4), this is a well-powered study. The most recent version of open-source software G*Power can be downloaded from http://www.psycho.uni-duesseldorf.de/abteilungen/aap/gpower3/. See Supplementary Fig. 4 with step-by-step process to compute powers herein shown. Examples for power and sample size for studies with individually caged mice intended for ANOVA or regression analysis are available at https://stats.idre.ucla.edu/other/gpower/.

*‘House one mouse per cage (unless more mice per cage is scientifically justifiable) and increase the number of cages per group (instead of few cages co-housing many mice which results in cage clustered-correlated data, lower study power and requires more mice to compensate for study power loss) to maximize the experimental and statistical value of each animal as a test subject during experimentation’*. The expert-prediction for implementation is moderately significant (grade, 7.7 ± 0.56; 91.4% probability of higher score vs. random; significant in 63.3% of simulations, p = 0.086 ± 0.13). The goal would be to maximize the scientific/test value of each mouse by promoting individual housing, emphasizing that social stress has been equally demonstrated, irrespective of sex, for single- and socially-housed mice^69,70^, and to promote the use of study power through cost-effective, reproducible experiments. As expected, this recommendation elicited the most heterogeneous responses, reflecting a partial reluctance to modify current animal density practices (Fig. 7b). To promote implementation and facilitate the accuracy/reproducibility of reports, we provide three graphical examples of why/how-to compute and report power/sample sizes for any completed experiment using single-caged mice and intuitive open-access software (‘G*power’^71^ in Fig. 8a–c, R^72^, and our STATA code below).

#### Action theme 6 on ‘Implementing statistical models to consider ICC in clustered data’

*‘Use statistical methods designed for analyzing clustered data when multiple mice are housed in one cage, and when data points are obtained from mice over time, to (i) properly assess treatment effects, (ii) determine the intraclass correlation coefficient (ICC) for each study, and then (iii) to use that information to rapidly generate experiment-specific, customizable study power tables to aid in the assessment, re-/design (if more mice or cages are needed), and reporting of adequately powered studies’*. The goal would be to promote and facilitate the implementation of cage-clustered data analysis in mouse research by (i) providing examples demonstrating the misleading effect when univariate methods are used for clustered-mice, and by (ii) making our statistical code available to the public to gain familiarity with protocol principles of cluster-data statistical tools. Recommendation six is intended to serve as a technical guide supporting the framework, and therefore was not tested for implementability.

**Figure 9:**
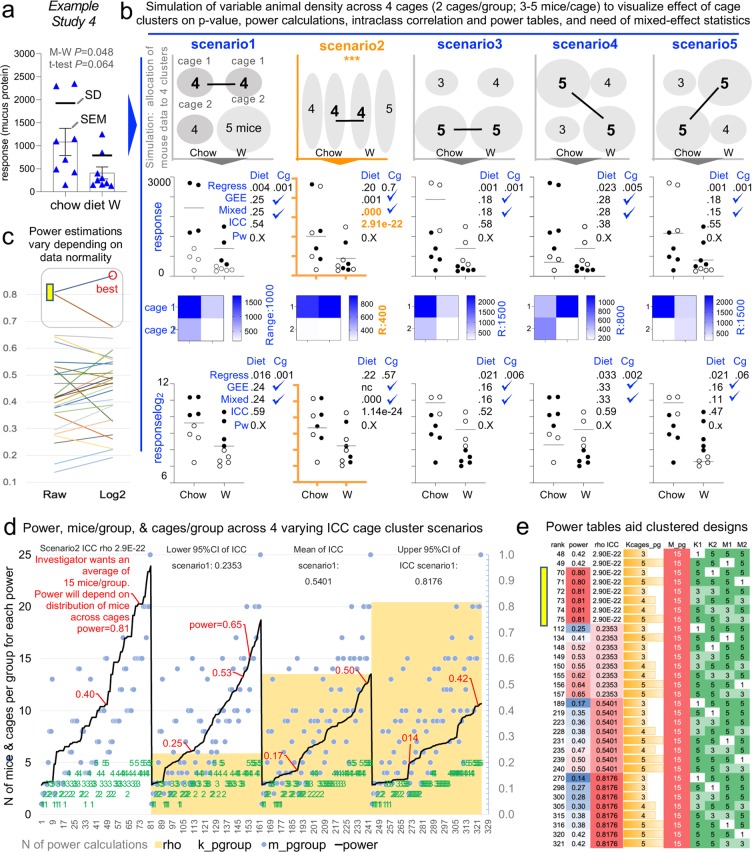
Analysis of cage-clustered data, intra-class correlation coefficients and power tables to facilitate study design by the number of cages/group and mice/cage. Five scenarios using a single dataset where mice housed as 5 mice/cage illustrate the effect of cage clustered data. Raw data extrapolated from one of the 172 reviewed studies. (**a**) Extrapolated raw data (original published dot plot; p-value = 0.059). Note that data from the diet ‘W’ group was not normally distributed (A-D p = 0.005). (**b**) Graphical representation of 5 scenarios considering different cage allocations of 2 cages/group. P < 0.05 for the regression analysis (‘Regress’) indicates cage effect. Except for scenario 2 (with mice representing the entire data range spectrum for treatment outcome ‘response’; y-axis), note that all scenarios are subject to significant cage effect. ‘Diet’, treatment; ‘Cg’, Cage effect. (**c**) Paired line plot depicting power estimates for the same data, without transformation (left) and after log2 transformation (right). Note the best power estimation for raw data may have a marked influence on the power estimation based on log2. We advise log2 transformed data for this dataset. (**d**) Line plot depicting power calculations for three ICCs, as a function of ICC, (histogram). Power estimations depend on ICC, simulation determined the n MxCg and TCgxGr needed to achieve a study power, which changes depending on degree of cage clustering (i.e., ICC, intra-class correlation coefficients). (**e**) Power table illustrates the number and distribution of mice using a clustered design to achieve study power.

To expand the outreach of our multi-theme framework, and to support scientists with their analysis and publication of justifiable/clustered experiments, we recommend to analyze and follow the statistical example we provide. The example is based on data extracted (using ImageJ^73^ analysis) from a published dot plot figure in a reviewed study that exclusively reported cohousing 5 MxCg, and where authors compared two diets using 8 and 9 MxGr (2 TCgxGr; Fig. 9a). The published p-value was 0.058, but to emphasize our message, we slightly/evenly adjusted the extrapolated data to achieve a univariate p < 0.050. By simulating 5 possible cage-clustering scenarios, Fig. 8 was designed to help visually understand the benefits of computing ICC and experiment-specific customizable power tables to determine whether more cages/group or mice/cage are needed to achieve study powers of ideally >0.8.

When using clustered-data methods, we showed that only one of the five scenarios yielded a significant diet treatment effect (*i.e*., scenario 2, where all cages were unbiased, having mice with high and low response values, something unlikely to occur naturally in clustered settings, Fig. 9b). Data proves that artificial heterogeneity due to mouse caging and unsupervised ‘cage-effects’ lead to poor reproducibility (80% of cases would misleadingly show that the test diet induces an effect on the mouse response). Graphically, we show that the variability of ICC (computed after running the mixed-effect models) depends on the hypothetical mouse allocation to cages, which in turn influences the post-hoc estimations of study power (Fig. 9c,d).

As a final practical product in this manuscript, we provide the statistical scheme/code in the GitHub repository (https://github.com/axr503/cagecluster_powercode) to implement this streamlined analysis and compute comprehensive power tables based on the ICC derived for each simulation to help scientists determine the best mice-to-cage combinations to match resources (Fig. 9e).

**Figure 10:**
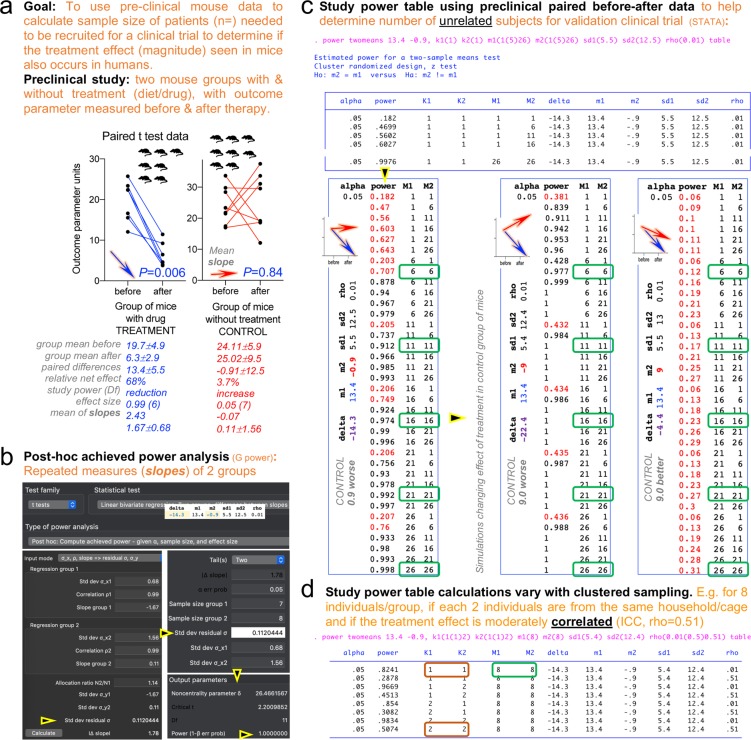
Translational analysis of preclinical mouse data to determine sample size estimations to validate in a human clinical trial. Treatment-control in two mouse groups (1 mouse/cage) before-after data (see Discussion). (**a)** Description of mouse study to plan human clinical trial that needs to be statistically supported/reported in an NIH grant. In this example, the best statistical strategy is to use paired (repeated-measures) data t-test. Notice control diet had no effect, which contrasted the significant effect induced by treatment (68% reduction on [inflammation] outcome). Post-hoc power analyses were conducted for each plot using paired-data t-test functions in G*power. (**b)** Integration of the two groups to determine overall study power was conducted using t-test linear bivariate regression (paired-data, two groups) using mean ± SD of all (before-after) slopes [computed in Excel using = slope(); = rsq(); = correl() functions]. G*Power screenshots illustrate input data needed and the output. Notice overall study power is perfect, *i.e*., 1.0. (**c)** STATA study power tables based on mouse data effect from panel ‘a’, and two simulations where controls had different outcomes. This example is for non-clustered sampling, *i.e*., K = 1. 1 mouse/cage. Red font, power <0.8. Simple one-line code in STATA (*power twomeans*) generates power tables that integrate cluster effects and ICC (‘rho’) when individuals cluster in households/cages (‘K’, cluster; K1(1), one-individual/cluster group 1; K1(2), two individuals/cluster group 1; ‘M’, individuals/group; ‘m’, group mean; ‘delta’, effect difference between groups; ‘rho’, ICC). Notice study power changes in each scenario after more patients are added to the study. If the effect observed in mice reproduces in humans at the same rate (68% improvement in treat/diet patients; 3.7% in control diet), the study could be stopped after 11 patients/group, because it would be unethical to not treat controls with the beneficial diet. However, if control diet has a different/unexpected effect, new study power tables could be generated based on data from first patients to adjust recruitment decisions. (**d**) STATA study power and sample sizes of patients/mice to be recruited varies if individuals cluster in households/cages and if the outcome is expected to be more/less similar between individuals within clusters (rho = 0.01, nonsimilar; rho = 0.51 more similar).

Thus far, we have examined the relevance of husbandry in mouse research practices. However, preclinical studies with animals are intended to inform human clinical trials, which are considered the “gold standard” in medical research. The observed effect size of interventions in animals (mean ± SD of treatment effect, % of improvement) may be used to initiate the design of clinical trials. Statistically, preclinical data may help determine the number of patients that need to be recruited (sample size estimations) to verify if the effects observed in animals also occur in humans. Variations on the implementability of this preclinical strategy to inform clinicians exist and could be complemented and paired with data derived from human pilot studies, or interim analysis starting with the first patients entering a study, which can be repeated at intervals through the study^74^. Herein, we provide an example of how dietary interventions in a ‘humanized’ IBD mouse model^6,75^ have been used to document the sample size calculations for patients during the implementation of the actual clinical trial, and for the seeking of federal grants that require statistical support sections in this regard. Figure 10a–d is an extension of the aforementioned scripts produced in G*power software followed by integration with STATA. The figure is an example of mouse data (treatment vs control in two mouse groups, 1 mouse/cage) in a preclinical study of a diet used to determine sample size estimations to validate the findings (good therapeutic effect) in a human clinical trial for which no human data existed.

Our study thus illustrates a translational strategy that links animal-based data and the need of physicians to estimate sample sizes and recruiting of patients for clinical trials for which various methods and issues have been described^74^. A ‘quick reference’ of actionable steps for all six themes is in Supplementary Table 8. To expand our implementability strategy for continuous assessment by the international scientific community the survey is available online (https://forms.gle/LxPCydbySddcndZ7A).

## Discussion

This study and proposed framework were motivated by the identification of a wide heterogeneity in published methods relevant to diet, microbiome, and the pathogenesis of inflammatory bowel diseases and digestive health in humans, where mice models are critical to study diseases biology, translational interventions, or to inform clinical trials for humans^75^. The action framework herein described as SART, specifically applies to promote the use of study power and improve reproducibility in any field of modern mouse research. Although it is impossible to develop a single consensus statement on practices pertaining to experimental supplies (*e.g*., bedding type, water, facilities) to accommodate every scientific community/goal, and not every mouse study involves the microbiome and there are mouse phenomena that are less affected by cage-level effects (for example, brain tumor studies initiated in genetically engineered mouse models), our proposed implementability statistics indicate that our 6 actionable recommendation themes could be widely adopted (possibly by >70–80% of investigators) to reduce the deleterious impact of these emerging concepts on artificial heterogeneity. This framework especially designed around reducing animal density, cage dirtiness, and cage microbiome bias, stresses the need of statistical methods for power and cluster data.

Herein, we confirmed that research methodology continues to vary in published literature, and as documented by a survey of academicians, such variability may be attributed to well-ingrained heterogeneous perceptions among scientists concerning how animal husbandry impacts the mouse microbiome. Animal density and the cost dilemma of how many cages are used to test hypotheses in experiments were deemed amenable for improvement. Because adjustments to facility settings are not easy to standardize, we propose that the most experimentally effective strategy to improve study power/reproducibility in the literature is to implement a lower number of mice per cage. From our analyses, we provide recommendation themes to minimize cage-clustering effects and implement clustered data analysis methods as a means to reduce artificial heterogeneity. To address the above challenges surrounding microbiome research and the experimental environment, we have drafted six thematic concepts that could be put forth for discussion as part of a consensus effort in the future by various professional organizations.

### Action theme 1 on ‘Reporting of diet and husbandry factors’

Reproducibility will occur only if critical study details are provided in published literature. Our review of studies combined with the high number of ARRIVE guideline^52^ citations (>7000) indicates that while ‘checklists’ may improve reporting quality, they do not ensure reporting with sufficient/consistent detail. A template paragraph for reporting would enforce uniform transparency, reproducibility, and enable rapid data mining for future meta-analyses, widely used to help guide the practice of medicine, but scarcely used in basic science.

### Action theme 2 on ‘Cage-cage microbiome variability BEFORE mouse experiments’

Fecal bacterial profiles can differ widely between cages within a single mouse strain housed under identical conditions and occurs even across mice produced for experimentation in contained breeding colonies^5,6,76^. Our survey demonstrated that although scientists implement strategies to control for cage microbiome variability before experiments^5,58,59^, there is ample variability of arguably reproducible method combinations used across organizations. Littermates have been proposed as an ideal for use as control groups^77^, however, numerous technical aspects (*e.g*., poor breeding colonies, small litter sizes, males fighting especially in certain genetic lines) frequently prevent this practice. As an alternative, or complementary option when littermates are available, a fecal homogenization protocol wherein all mice are administered a composite of freshly collected feces via oral gavage for 3 days^57^, has been shown to effectively minimize inter-cage gut microbiota heterogeneity before experimentation^57,58,67,78^.

### Action theme 3 on ‘“Dirty cages” and time of sampling DURING experiments’

Our survey showed ample heterogeneity in timing of mouse cage sanitation protocols despite recent studies indicating that bedding soiledness (‘dirtiness’) contributes to periodic variations in gut microbiome via contact/coprophagia^4,55^. Mouse experiments would benefit if conducted with cages having reduced animal density (1–2 MxCg) with biological samples systematically collected from clean cages at the same time of day to avoid diurnal variation^79–81^.

### Action theme 4 on ‘Repeating experiments in different seasons’

As reflected by the literature reviewed and the misconceptions documented in our survey, little is known about the effect of time of year/season on mouse research heterogeneity^6,76,82^. Since it is almost impossible to control for seasonal variation within long-term, or multiple short-term experiments spanning over several seasons, it is important to take measures to improve study variation/reproducibility over time (*e.g*., food batch, inter-experiment IsPreFeH).

### Action theme 5 on ‘Animal density, clusters, and study power’

First, our scoping review identified numerous laboratories publishing clustered MxCg data with few cages/groups, without the verification of study power/sample sizes, or use of statistics for clustered-data. Then, our survey and cost simulator showed financial-scientific discordance among scientists when deciding animal densities. Unless higher densities are scientifically (not only financially) justifiable, housing 1 MxCg could yield more cost-effective and powerful study designs by increasing the number of cages and minimizing the need to use advanced statistics^50,75,83^. Although adding more cages to a study increases handling costs, studying *‘less mice per cage is more’* is a pro-statistically powerful, comparably effective practice^75^. The use of cost as a rationale for conducting cage-clustered experiments needs conscientious consideration, since housing costs are just a fraction of the research funds required for tests. Perhaps, institutions could provide discounts to investigators for the cost of housing when conducting experiments, because fewer MxCg requires less cage changes and experiments are often short-term. Logistically, since fewer MxCg may be an option limited by space in certain facilities, well-powered and well-analyzed cage-cluster studies is desirable.

### Action theme 6 on ‘Implementing statistical models to consider ICC in clustered data’

Depending on the experiment, we recognize that it is not always possible to single-house mice. Our review showed that scientists often analyze clustered observations using methods that mathematically function under the assumption of data independence (student T-, Mann-Whitney, One-/Two-way ANOVAs), without implementing statistics for intra-class (‘intra-cage’) correlated (ICC) cage-clustered data (Multivariable linear/logistic, Marginal, Generalized Estimating Equations, or Mixed Random/Fixed Regressions)^50,84,85^. The ICC describes how units in a cluster resemble one another, and can be interpreted as the fraction of the total variance due to variation between clusters^50^. Housing multiple MxCg as homogeneous densities across study groups is logistically challenging using few cages.

As identified in the literature review, the statistical description in peer-review mouse studies is frequently suboptimal. The examples here provided were designed to assist scientists address this limitation. As an advanced example relevant to translational research, Fig. 10a is a case of an outcome measured in mice before and after treatment, which can be best analyzed by using the slope of the effect (as in repeated measures), or the net difference/change of the outcome measured (after-minus-before) to aid determine sample sizes for pilot clinical trials. Of practical value, G*power has statistical tests based on means and slopes, which can also be used to draw informative sample size-vs-power curves, and integrate repeated-measure studies with two groups (Fig. 10b). A simple line of code in STATA, in turn, however generates comprehensive study power tables to assist scientists determine the number of patients to recruit, and based on interim study power estimations, if the study needs to be halted earlier (due to more significant than expected, or non-significant effects) as patients are recruited. The STATA power tables can be used to document the analysis requested in NIH/similar grants or publications, when needed for the planning and justification of sample sizes of animals or humans. This is especially relevant to transitional science, because the tables integrate cluster effects and intra-class correlation coefficients (ICC, ‘rho’) in scenarios where individuals are clustered in households/cages, which ultimately influences the study power and the decisions to be made in follow-up interim analyses, for instance, after recruiting the first (*e.g*., 6, 11, 16, 21 or 26) individuals as shown in Fig. 10c for two groups. Study power and sample size estimates vary depending on whether individuals are sampled clustered in households/cages, and if the outcome is expected to be more or less similar between individuals within a cluster (rho = 0.01, non-similar; rho = 0.51 more similar, rho = 1, highly similar).

The notion that the gut microbiome influences mouse phenotypes/physiology is not a new concept and has been discussed in the past. For example, a 2016 perspective article accounting for reciprocal host–microbiome interactions in experimental science^77^ provided conceptual examples on different aspects that influence the microbiome biology during experimentation and different factors that influence host communities, such as diet, maternal transfer and host genetics, and emphasized the importance of GF animals to study microbes illustrating that GF studies do not account for the effect of microbes on host development. In the present paper, we integrated peer reviewed data with survey data from practitioners primarily in the USA via expert panel, and provide analytical insights and concrete statistical examples with their respective code.

We acknowledge that aspects described in this paper may not always be applicable to every research scenario or experimental objective. For example, studies involving morphological aspects of brain biology and development may not be subject to the same modulatory effect by the gut microbiome as one could anticipate in a study of intestinal mucosal biology. We emphasize again that the recommendation themes, succinctly discussed below, are not derived from a formal consensus as it is customary for societies to guide medical practice. However, as a unique contribution to the body of literature in husbandry and reproducibility is the emphasis we are placing on the use of statistical study power. Power study tables have helped scientists make decisions with respect to study design and execution of experiments to prevent and control bias, data interpretation, and the use of data derived from animals to inform the potential effects in human clinical trials.

In conclusion, we confirmed that research methodology continues to vary in published literature and as documented by a survey of academicians. Analyses indicate that the reporting of post-hoc study power calculations, in the context of the proposed framework, could be objectively used to guide and monitor the research power and reproducibility across mouse microbiome research at large. The examples here are expected to contribute to improvement of methods reproducibility, results reproducibility, and inferential reproducibility as previously described^3^. As a unique contribution, here we provide specific numeric and graphic examples to illustrate these concepts, and the means by which improper analysis may lead to spurious analysis or interpretations which in turn affect reproducibility. Our statistical illustrations highlight concepts on analytical problems (with their practical solutions) which frequently affect methods-, results- and inferential-reproducibility.

## Materials and Methods

### Study overview

As an overall methodological strategy to confirm and quantify the extent to which animal husbandry variability has been, and continues to be, present in mouse and microbiome research, we first conducted a quantitative verification of animal husbandry variability in academia (i) by screening the recent published peer-reviewed literature (2018–2019) to infer the historic prevalence of prevailing practices that could have influenced research and (ii) by conducting a survey of academicians across relevant professional organizations to determine the present status on beliefs and knowledge on husbandry practices. Then, we ranked the practices based on relevance to influence microbiome research, as perceived by respondents, to prioritize/make six recommendations. Lastly, to document the validity of such recommendations, we conducted a targeted literature search to cite examples enabling the analysis of such suggestions in future consensus efforts. Using a Delphi-based consensus strategy, these suggestions were graded for quality to compute heterogeneity and probability statistics for implementability by investigators. See Fig. 1 for illustrated study overview.

### Quantification of husbandry methods heterogeneity

As a test topic, we chose to use ‘dietary studies in mice’ as PubMed search terms to screen (scoping review) original peer-review studies for animal husbandry practices as of May 3^rd^, 2019, published literature (see references of identified studies in Supplementary Materials) To interrogate and quantify perceptions and opinions among academicians on animal husbandry practices that influence microbiome data variability, a one-time online IRB-approved survey with 11 multiple-choice questions was administered, via recruitment email, to eligible participants through membership list servers of the following: (i) faculty of 17 NIH National Institute Diabetes and Digestive and Kidney Diseases (NIDDK) Silvio O’Conte Digestive Diseases Research Core Centers (‘DDRCC’), which provide research support to local and national institutions, (ii) registrants of the 2018 Cleveland International Digestive Education and Science (IDEAS) Symposium hosted by the Cleveland DDRCC, Case Western Reserve University (CWRU), (iii) registrants of the Taconic Biosciences Webinar titled ‘Cyclical Bias and Variability in Microbiome Research’, (iv) members of the American Association of Laboratory Animal Science (‘AALAS’), and (v) members of ‘GNOTOBIOTIC’ ListServ, forum of the National Gnotobiotics Association.

### Six evidence-based recommendations graded for future implementability

To provide evidence-based suggestions and to support the development of a large-scale consensus report that can be implemented and beneficial to research, we used a ranking of the survey-derived husbandry practices to prioritize the husbandry topics deemed influential in mouse microbiome by respondents. Using Google PubMed and keywords contained in the survey question/topic (*e.g*., mouse, water), five coauthors cataloged relevant peer-reviewed scientific articles on each topic (targeted review). The information gathered, as tables, was used as assessment tools by 14 individuals to grade a table with 5-recommendations drafted by the lead and senior authors in this study. Collectively, the individuals comprised professional experiences across five research institutions; CWRU, The Scripps Research Institute, Kyorin University, South Dakota State University, The Ohio State University, University of Chicago, and Cornell University. To determine if the 5-recommendations could be implemented as a framework, individuals were asked to provide suggestions, new recommendations, and to grade (1, low; 10, highest) each item for sentence clarity, potential impact, and recommendability to others (Supplementary Materials). These ‘implementability grades’ numerically illustrate the potential for variance and adoption of the recommendations by others in mouse research. The sixth recommendation was constructed in response to the suggestions made by the implementability and study ‘blind’ anonymous reviewers.

### Ethical considerations

All research was approved by the Case Western Reserve University Institutional Review Board (STUDY20180138). All methods were carried out in accordance with the relevant guidelines and regulations. Informed consent was obtained from all participants.

### Statistics

For computation purposes, animal/cage density data extracted from the scoping review were used to create a secondary index. Specifically, the number of animals per group (group size, MxGr) and the number of mice housed per cage (animal density, MxCg) were used to compute a semi-descriptive index metric of ‘cage cluster effect’ on each study: ‘estimated number of cages per experimental group’ (*i.e*., total n of cage clusters per group, TCgxGr = MxGr divided by MxCg). If a range was provided for animal density (*e.g*., 1–5), estimations were computed using the median value within the range, as well as the minimum and maximum values. Average of estimated center values were used for analysis and graphical summaries. For Fig. 9, study selection was based on the use of 5 mice/cage, and that study results were published as dot plots (allowing us to infer the raw data for our analysis) in the manuscript. Descriptive statistics for parametric data were employed if assumptions were fulfilled (*e.g*., 1-way ANOVA). Non-fulfilled assumptions were addressed with nonparametric methods (*e.g*., Kruskal-Wallis). As needed, 95% confidence intervals are reported to account for sample size (*e.g*., MxCg; surveyed participants) and for external validity context. Significance was held at p < 0.05. Analysis, study powers, and graphics were conducted with R, STATA, Python 3.0 Anaconda, GraphPad and G*Power^71^. G*Power is an open-source power specialized software for various family of tests; calculations only require p-value (alpha), sample size, and mean ± SD to compute effect size. Excel was used to create a cage handling frequency and cost spreadsheet calculator.

### Permissions

We confirm that all drawings in manuscript figures were drawn by the corresponding author or one of the co-authors.

### Preprint

Motivated by Springer Nature Publishing policies*** on preprint sharing, and their encouragement to integrate input from the scientific community into formal peer-review at Nature Communications, section Scientific Community, we have shared the herein improved manuscript version, as a preprint. (bioRxiv preprint first posted online Sep. 25, 2019; 10.1101/778043). ***Nature 569, 307 (2019), 10.1038/d41586-019-01493-z.

## Supplementary information

Supplementary Materials.

## Data Availability

The authors will make all data available upon request.
