# Supplementary material for: Artificial microbiome heterogeneity spurs six practical action themes and examples to increase study power-driven reproducibility
Source: Sci Rep. 2020 Mar 19;10:5039. doi: 10.1038/s41598-020-60900-y (PMC7081340; doi:10.1038/s41598-020-60900-y)
Supplement: Supplementary file 1 — Supplementary Materials. [file 41598_2020_60900_MOESM1_ESM.pdf]

Artificial microbiome heterogeneity spurs six practical action themes and examples to increase study power-driven reproducibility

Abigail R Basson[1,2,3] Alexandria LaSalla[1], Gretchen Lam[1], Danielle Kulpins[1], Erika L Moen[5], Mark Sundrud[5], Jun Miyoshi[6], Sanja Ilic[7], Betty R. Theriault[8], Fabio Cominelli[1,2,3], Alexander Rodriguez-Palacios*[1,2,4]

[1] Division of Gastroenterology & Liver Diseases, Case Western Reserve University School of Medicine, Cleveland, OH, USA.
[2] Germ-free and Gut Microbiome Core, Digestive Health Research Institute, Case Western Reserve University, Cleveland, OH, USA.
[3] Digestive Health Institute, University Hospitals Cleveland Medical Center, Cleveland, OH, USA.
[4] Mouse Models Core, Silvio O'Conte Cleveland Digestive Diseases Research Core Center, Cleveland, OH, USA.
[5] Department of Biomedical Data Science, Geisel School of Medicine, The Dartmouth Institute for Health Policy and Clinical Practice, Lebanon, NH, USA
[6] Department of Immunology and Microbiology, The Scripps Research Institute, Jupiter, FL, USA
[7] Department of Gastroenterology and Hepatology, Kyorin University School of Medicine, Tokyo, Japan.
[8] Department of Human Sciences and Nutrition, The Ohio State University, Columbus, OH, USA.
[9] Department of Surgery, University of Chicago, Chicago, IL, USA.

***Corresponding Author**: Alexander Rodriguez-Palacios (axr503@case.edu)

Supplementary Materials

1. **Supplementary Methods**
2. **Supplementary Figures**
    a. **Supplementary Figure 1.** Literature review of studies in 'diet gut microbiome and mice' illustrates heterogeneity in animal husbandry in experiments (complement to data presented in Figure 2).
    b. **Supplementary Figure 2**. Physical appearance of survey pertaining to opinions/beliefs and animal facilities.
    c. **Supplementary Figure 3.** Accumulation of organic matter (feces) in cage bedding.
    d. **Supplementary Figure 4.** Screenshot of open-source power calculator G*Power (complement to data presented in Figure 8 and Figure 10).
3. **Supplementary Table**
    a. **Supplementary Table 1.** Five leading causes of cage-cage variability ('cage effect') in mouse research relevant to experiments and simple solutions*
    b. **Supplementary Table 2.** Microbiome variability: sources of cage-cage differences prior/during experimentation table for expert grading of five 'evidence-based' Recommendations for sentence clarity and potential benefit to increase study power and reproducibility in mouse research.
    c. **Supplementary Table 3.** Table for expert grading of proposed action theme recommendations for 'sentence clarity', 'potential benefit to increase study power' and 'reproducibility' in mouse research
    d. **Supplementary Table 4**. Compilation of comments made by individuals that graded the initially drafted five Recommendations
    e. **Supplementary Table 5.** Evidence-based husbandry suggestions to improve study power **Supplementary Table 6.** Rationale for action theme recommendations **Supplementary Table 7.** Methodology reporting template
    f. **Supplementary Table 8.** Summary of actionable items for each action theme recommendation
4. **Supplementary Discussion**
    a. Suppl. discussion on Recommendation 1 on reporting and diet.
        i. 1.1 Type of water and decontamination
    b. Suppl. discussion on Recommendation 2 on cage microbiome variability
    c. Suppl. discussion on Recommendation 3 on cage dirtiness and time of sampling
        i. 3.1 Cage change frequency and bedding 'soiledness' (dirtiness).
        ii. 3.2 Timing of Fecal Collection.
        iii. 3.3 Cage Bedding Material.
        iv. 3.4 Environmental temperature and humidity.
        v. 3.5 Cage Ventilation.
        vi. 3.6 Cage Type.
    d. Suppl. discussion on Recommendation 4 on repeating experiments.
    e. Suppl. discussion on Recommendation 5 on animal density, clusters, and study power
        i. 4.1 Housing density and cage effect.
        ii. 4.2 Animal well-being.
5. **Supplementary References**

1. Supplementary Methods

Historic and quantitative verification of husbandry variability in published literature. As a test topic, we chose to use 'dietary studies in mice' as a theme to screen for reporting animal husbandry practices in the published literature. Original peer-review research publications were identified using the search terms 'diet and gut microbiome and mice' in PubMed. Abstract screening, and then Article review and data extraction were conducted by two authors (AL, GL) under the supervision of AB and iterative testing/training and problem-solving sessions with ARP. Studies were included if they met the following criteria/definition: 'original article examining the gut microbial community in fecal or cecum samples in mice administered a diet-based intervention'. Studies published in letter or abstract format were excluded as they did not include enough information on animal husbandry for our review. Extracted study information was conducted using a standardized extraction form and included the following variables**;** ***i)*** study location (country), ***ii)*** whether the diet intervention altered the microbiome, ***iii)*** number of mice per cage, ***iv)*** number of mice per experimental group, as well as reporting of; ***v)*** sterile diets (autoclave, irradiated), ***vi)*** water source and decontamination process, ***vii)*** frequency of bedding change, ***vii)*** light/dark cycle, ***ix)*** room temperature, ***x)*** room humidity, ***xi)*** season/time of year, ***xii)*** mouse strain and GF status of mice. Where appropriate, binary interpretation of data was used for quantitative assessment.

Verification of current husbandry variability among academicians via electronic survey. A one-time online survey with multiple-choice questions pertaining to opinions on animal husbandry practices that influence microbiome data variability was used. Survey questions are described within the results section (and in Supplementary Materials). All research was approved by the Case Western Reserve University Institutional Review Board (STUDY20180138). The survey was administered via secure email (Google Survey) links to eligible participants between August and November in 2018. Informed electronic consent preceded the voluntary, anonymous, and pretested (with 4 individuals) survey. All subjects had the option to withdraw at any time; and no compensation was provided. Eligible participants were >18 years old and recruited via email using the following list servers were used: *i)* membership lists of faculty affiliated to the 17 NIH National Institute Diabetes and Digestive and Kidney Diseases (NIDDK) Silvio O'Conte Digestive Diseases Research Core Centers (DDRCC), which provide research support to investigators in local and national institutions ***ii)*** registrants and attendees of the 2018 Cleveland International Digestive Education and Science (IDEAS) Symposium hosted by the Cleveland DDRCC, CWRU (September 16th- 18th 2018)***, iii)*** registrants of the Taconic Biosciences Webinar (August, Thursday 16th, 2018) titled 'Cyclical Bias and Variability in Microbiome Research', ***iv)*** members and affiliates of the American Association of Laboratory Animal Science (AALAS), and ***v)*** members of the 'Gnotobiotics ListServ', an open professional forum affiliated with the National Gnotobiotics Association. Incomplete surveys were discarded. All multiple-choice questions had an "I do not know" option. A total of 3 follow up reminder emails were sent.

Literature to support evidence-based expert-graded recommendations. To provide evidence-based recommendations and support the development of a consensus report that can be implemented throughout microbiome research practices, we prioritized topics using the ranking of husbandry practices derived from the survey analysis. Using the keywords contained in the individual survey question or topic (*e.g.,* mouse, water), as well as secondary screening of relevant papers using google search and grey literature performed by three individuals, we create a list of relevant supporting or contrasting evidence and potential solutions for consideration to be used as an information tool by the key experts to propose the number and the description/wording of overarching recommendations. Finally, a table containing the list of the core recommendations were submitted to external experts for comments and grading using a simplified objective Delphi protocol based on the agreement between experts. The presented recommendations are listed based on the average grading (ranked from 1 – 10, each Recommendation is graded separately for sentence structure, content, and experimental relevance as integrated solutions; 10 is the highest quality). Secondary experts were provided with the abstract of this manuscript.

2. Supplementary Figures

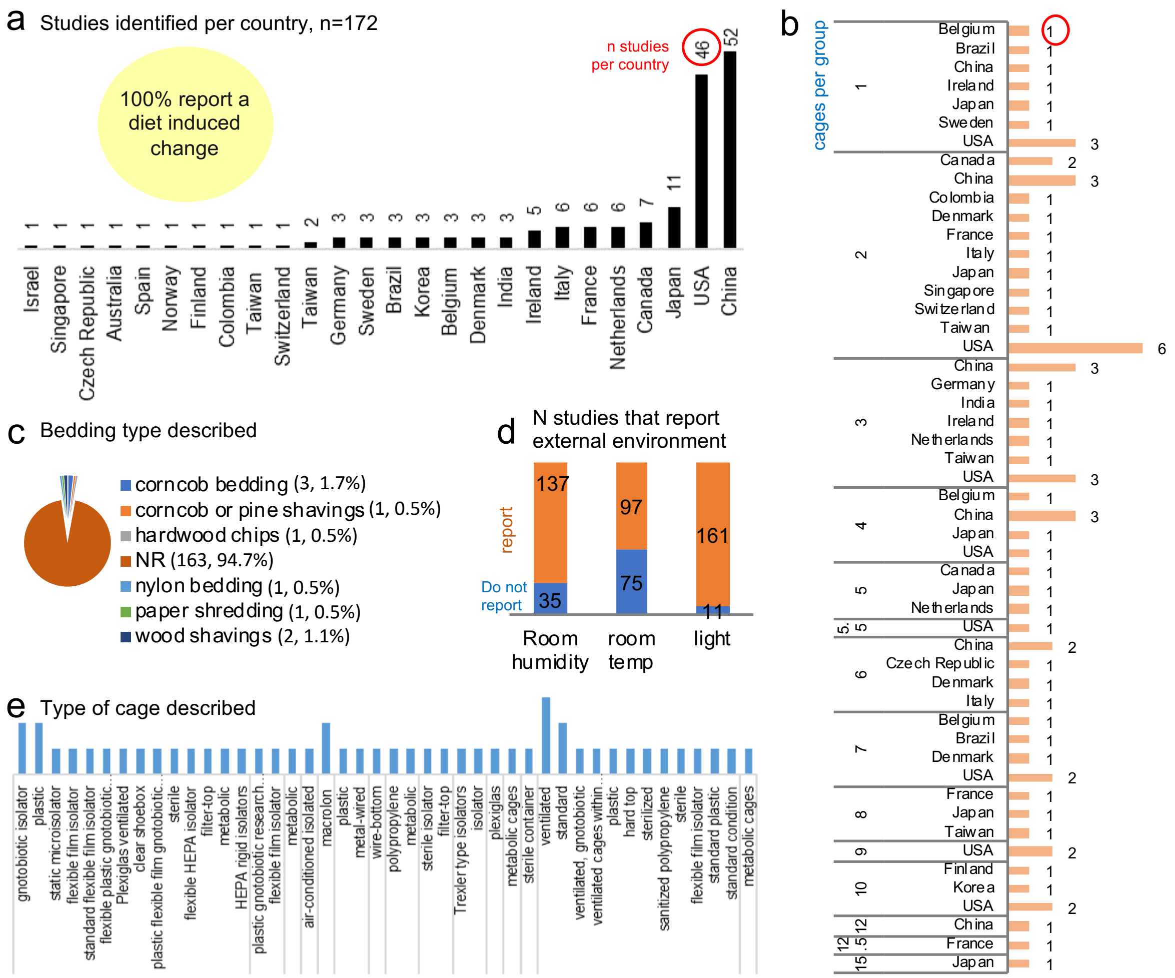

Supplementary Figure 1. Literature review of studies in 'diet gut microbiome and mice' illustrates heterogeneity in animal husbandry in experiments (complement to data presented in Figure 2). a) Distribution of studies identified per country. **b)** Average number of cages per experimental group used illustrated by country. **c)** Type of bedding described by studies that report bedding type. NR; not reported. **d)** Number of studies that do report vs. do not report aspects of external environment. **e)** Type of cage described when reported.

Opinions and Beliefs

Q1/11. Rank how important you believe each of the following aspects contribute to microbiome research variability (in mice). *

	1 - Not Important	2	3	4	5 - Very Important	I do not know
Animal diet composition	○	○	○	○	○	○
Sterility of diet during experiment	○	○	○	○	○	○
Drinking water	○	○	○	○	○	○
Type of animal cage	○	○	○	○	○	○
Cage bedding material	○	○	○	○	○	○
Frequency of bedding material replacement	○	○	○	○	○	○
Number of animals per cage	○	○	○	○	○	○
Animal room light/dark cycle	○	○	○	○	○	○
Room temperature	○	○	○	○	○	○
Room humidity	○	○	○	○	○	○
Cage ventilation system	○	○	○	○	○	○
Season/Time of year	○	○	○	○	○	○
Standardizing time of day for fecal collection (morning vs afternoon)	○	○	○	○	○	○
Amount of moisture/feces in cage bedding ('soiledness') when samples are collected	○	○	○	○	○	○
Coprophagia (mice eating their feces)	○	○	○	○	○	○

Opinions and Beliefs

Q2/11. In a 1-month diet experiment with 5 mice/group, which housing option do you believe is FINANCIALLY preferable? *

- ○ Five mice housed together in one cage (1 cage total)
- ○ Three mice housed in one cage, and 2 mice in another cage (2 cages total)
- ○ One mouse per cage (5 cages total)

Q3/11. In a 1-month diet experiment with 5 mice/group, which housing option do you believe is SCIENTIFICALLY preferable? *

- ○ Five mice housed together in one cage (1 cage total)
- ○ Three mice housed in one cage, and 2 mice in another cage (2 cages total)
- ○ One mouse per cage (5 cages total)

Animal Facilities

Q4/11. What kind of drinking water is most commonly used in your regular mouse (SPF/conventional) facility? *

- ○ Untreated tap water
- ○ Autoclaved tap water
- ○ Irradiated tap water
- ○ Acidified water
- ○ Reverse osmosis water
- ○ Distilled water
- ○ I do not know
- ○ Other: ______

Q5/11. How often is the cage bedding (or entire caging system including bedding) replaced during a mouse experiment? *

- ○ Daily
- ○ Every 2-3 days
- ○ Every 4-6 days
- ○ Weekly
- ○ Every two weeks
- ○ When it looks dirty (no set time frame)
- ○ I do not know

Supplementary Figure 2. Physical appearance of survey pertaining to opinions/beliefs and animal facilities. a) Ranking question on the importance of animal husbandry-related factors in variability of microbiome research**.** To understand academia perceptions, we formulated four questions, using 'diet composition' as a positive control because intuitively, we know the quality of responses would be reflected appropriately. The first was a ranking based question (from 1-to-5 with 1 being not important and 5 being very important) comprised of 11 variables pertaining to animal husbandry practices and each in context to the primary question; "Rank how important you believe each of the following aspects contribute to microbiome research variability (in mice)?**"**. **b)** Opinion questions on scientific vs. financial of different animal housing options. **c)** 'Animal facilities' question to determine facility-dependent practices on type of water used (Q4/11) and cage change frequency (Q5/11).

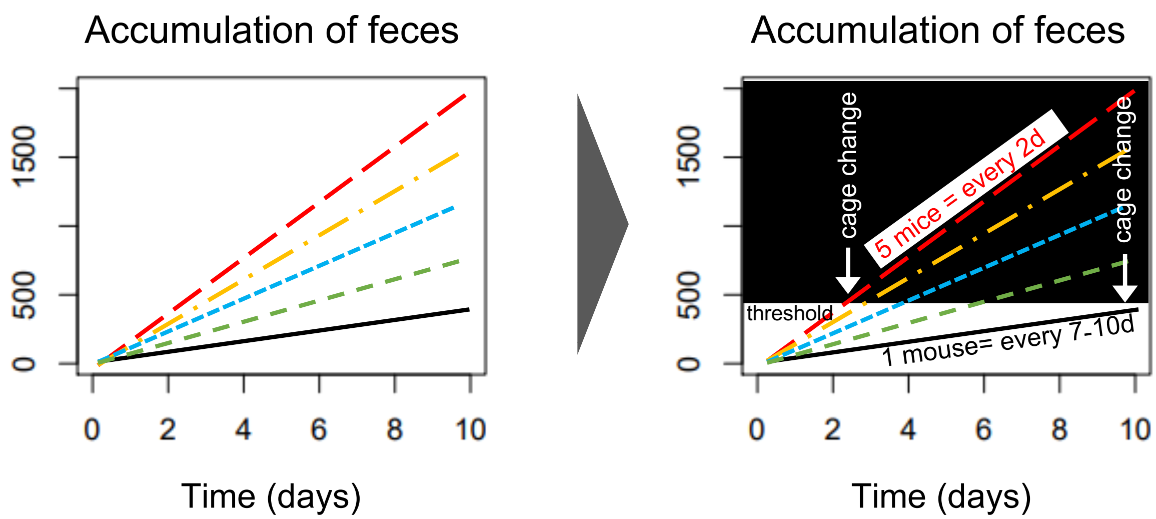

\

Supplementary Figure 3. Accumulation of organic matter (feces) in cage bedding. The plot illustrates the linear accumulation of fecal material which increases linearly based on the number of mice housed in one cage (assuming the production of feces by each mouse is consistent). Note that frequency of cage change increases with higher animal density based on the faster accumulation of fecal content in bedding. Cage changes represent a 'clean break' in bacterial overgrowth. From left to right, each line represents 5, 4, 3, 2 and 1 mouse projections. Adapted illustration.[1]

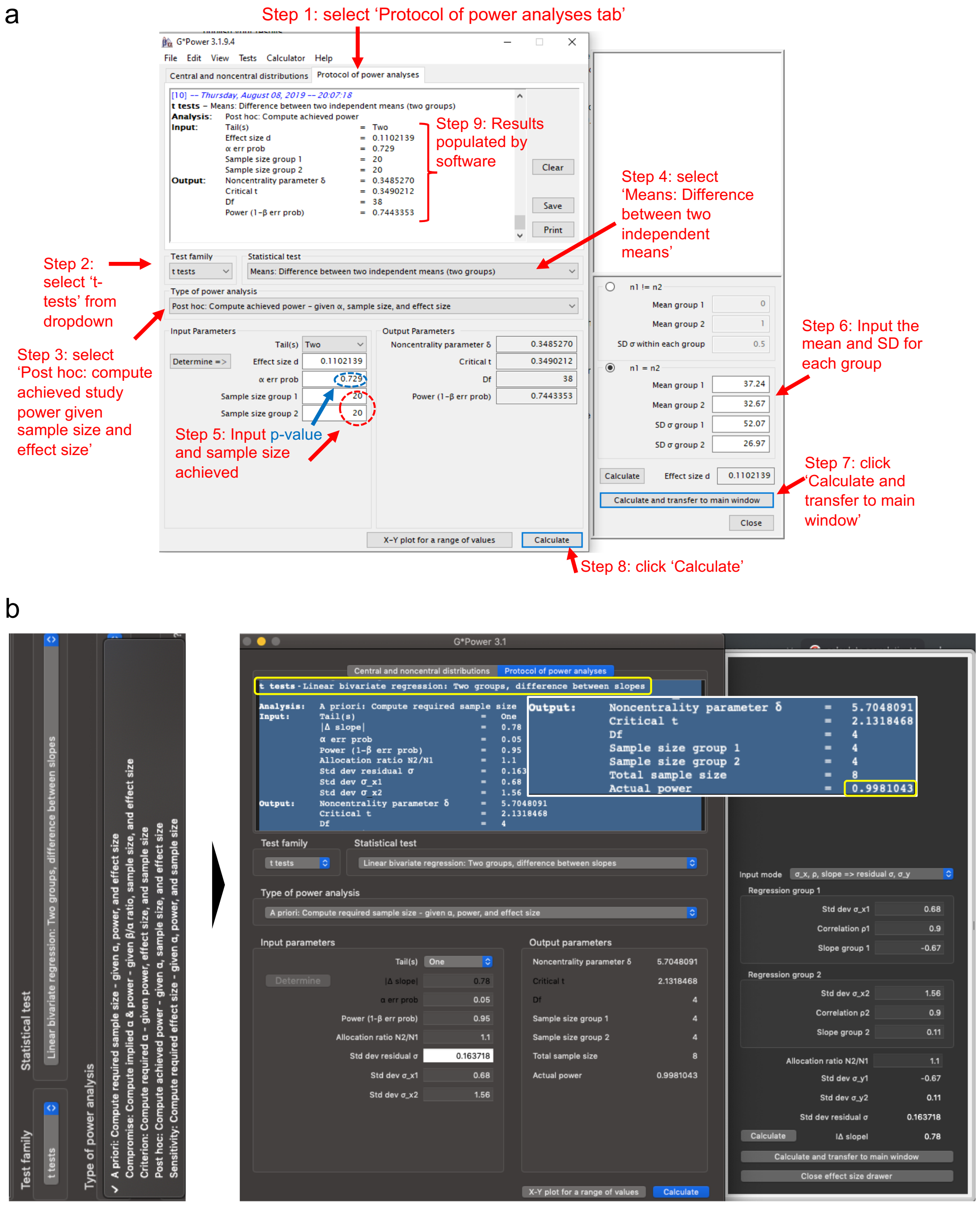

Supplementary Figure 4. Screen shot of open-source power calculator G*Power (complement to data presented in Figure 8). a) Screenshot of G*Power window for Figure 8A following completion of steps 1-8 (described above) and results populated by software. **b)** Screenshot of G*Power window for Figure 10 and results populated by software. Open-source software G*Power and detailed manual can be downloaded from https://www.gpower.hhu.de.

3. Supplementary Tables

Supplementary Table 1. Five leading causes of cage-cage variability ('cage effect') in mouse research relevant to experiments and simple solutions*

Animal husbandry factor & Comment on how element introduces variability and solutions
**Microbiota drifts and cage type**<br>Ammonia levels vary across cages based on cage differences in ventilation, animal density, bedding material, temperature, humidity (moisture level), fecal accumulation, diet, and microbiota.<br>**Variable consequences:** Variable high levels in ammonia have shown to disrupt the 'normal' mouse environment, causing stress, inhibition of small intestine motility, as well as affect upper respiratory functions.[2, 3] Cyclical 'dirty cage' effect.<br>**Solution**: Fecal homogenization. Here, it is important to consider the presence of immunomodulatory organisms (*e.g.*, SFB)[4], as well as richness and diversity of inoculum with differences in outcome transfer reported in mice colonized with simpler microbiota.[5]
**Cage position on cage rack**<br>Cages located in high shelves are exposed to *brighter light* and *warmer air*, (chimney effect, warm air floats), more *vibration*. The opposite applies to cages on bottom shelves. Cages next to corridor are exposed to *human traffic*; cages facing the wall are quieter. Not all *ventilation ports* in pressurized ventilated cages are expected to function equally.<br>**Variable consequences:** animal distraction such as sleep deprivation, visual & auditory stimulation, anxiety, and abnormal feeding/grooming. Closer cages to ventilator will have faster sir flow (less resistance to airflow.<br>**Solution:** 'The more cages, the better' in any experiment to increase heterogeneity and account for statistical errors (*i.e.*, statistical residuals in y=bo + b1x1 + E).
**Animal density/cage soiledness**<br>N of mice/cage affect Behavioral responses; hierarchy, aggression, and barbering. More mice in a cage will consume food faster, therefore more food replacements mean fresher diet.<br>**Variable consequences: High levels of ammonia (see above).** Coprophagia or coprophagy is considered a normal behavior in rodents and mice and refers to the consumption of expelled feces that are present in the animal cage. While the behavior has been shown to contribute to the animal's nutrition (B-vitamins),[6, 7] it also holds strong potential to introduce microbial variability in microbiome research studies, especially as the degree of cage bedding 'soiledness' (dirtiness) increases.[1] In addition, inter-individual variations in mouse gut microbiota are common in animals housed within the same facility room. To address coprophagy as a potential confounding factor, previous studies have proposed housing mice with floors of larger mesh size (to allow feces to fall through)[8] as well as to increase cage-change frequency.[1]<br>**Solution**: Individual housing of mice.
**Expected less controllable Cage-variable Batch effect**<br>Diet is an expected, but less controlled and reported variable that influences cage effect.<br>**Variable consequences:** Differences in nutritional composition, specifically fatty acids of commercial laboratory rodent diets have been shown to alter exocrine pancreatic function in rodents.[9] Equally, potential for batch-to-batch variability could exist with closed-formula commercial diets (ingredients not publicly available).[10] In this context, studies performed over extended periods may include data generated from diets comprised of different ingredients even if sourced from a single vendor. Diet quality (*i.e.,* ingredients, mold, micro toxins, dust, expiration) and storage of experimental diets (*i.e.,* were diets refrigerated or frozen upon receipt to ensure freshness throughout experiment) should also be considered whether using open- or closed-formula diets.[10, 11]<br>***Diet sterility.*** Intuitively, diet sterility is also an essential factor to minimize external microbial exposure to animals, with non-irradiated diets acting as a variable source of bacteria and fungi. Autoclaving and irradiation are used to improve diet sterility, although in-house autoclaving methods are not standardized and likely lead to variations in nutritional composition of the autoclaved diet.[12]<br>**Solution**: Every effort should be made to ensure diet sterility (autoclaving, irradiation).
**Unexpected sporadic/ accidental/non-anticipated sources of technical error**<br>Flooding (*e.g.*, accidental water bottle leak). Provision of incorrect bedding or diet treatment.

*Cages can experience different levels of noise, lighting, vibration, and humidity based on rack location. Differences in individual cage humidity levels are a factor of bedding moisture level, type of cage ventilation (static vs mechanically ventilated), number of mice per cage.

Supplementary Table 2. Microbiome variability: sources of cage-cage differences prior/during experimentation

Gut microbiota modulators	Comment
Vendor, Shipping and Institution	Vendor, shipping, the individual institution and facility have been shown to influence and alter gut microbiota with transfer of mice from one facility to another, resulting in microbial shifts.[13, 14] One of the most recognized differences is Segmented filamentous bacteria (SFB) presence (commercial vendors) or absence (Jackson Laboratory).[15] Facility-dependent differences in animal husbandry (*e.g.* caging, bedding, cage change freq.; refer to **Supplementary Table 1**), diet[16, 17] and water contamination methods.[18, 19]
Breeding	Established colonies harbor a stable microbiota over generations.[20] However, the introduction of new dams or a sire harboring difference microbiota can cause downstream variation in gut microbiota of pups. Host strain background, gender and mouse age also influence microbiome.[21]
Methods to eliminate unwanted bacteria	Bacterial elimination methods with downstream effects on gut bacteria include; ***i)*** Rederivation[22-24] and mode of pup delivery (cesarean vs. vaginal),[25] ***ii)*** transfer of mice between facilities or from conventional to barrier settings,[12] ***iii)*** resuscitation of mice from cryopreserved germplasm or *iv)* therapeutic antibiotic use to eliminate unwanted pathogens[26, 27] all hold potential to introduce microbial variation. Antibiotic efficacy is highly dependent on regime and baseline richness and diversity of microbiota before initiation.[12, 28, 29] More targeted measures include reconstituting germ-free mice with specific microbial communities and rederivation via surgical embryo transfer, although these are not without their disadvantages.[12]
Intestinal habitants	The presence or absence of certain members of the gut microbiota can dramatically modulate model immune response and phenotype.[30-32] and thus reproducibility These include; ***i)*** rodent **helicobacters** (*e.g., Helicobacter hepaticus),* [33-42] ***ii)*** **segmented filamentous bacteria** (see detailed review), [15] ***iii)*** certain **protozoans** (*e.g.*, *Tritrichomonas* spp.),[43, 44] ***iv)*** the **human isolates** (*Bifidobacterium adolescentis),* [45] and ***v)*** mouse parvoviurs (MPV).[46] It is also highly likely that multiple agents collectively contribute to model phenotype.[47, 48]

Co-housing and cross-fostering (transfer of pups to a timed-mating foster dam) are non-targeted solutions that cannot rule out the gut microbiota contribution to phenotype and should be accompanied by next-generation sequencing. See further rational and discussion on how the water type and sterility, which vary by vendor,[18, 19, 49-53] and the role of coprophagia influence the gut microbiota in **Supplementary discussion**.

Supplementary Table 3. Table for expert grading of proposed action theme recommendations for 'sentence clarity', 'potential benefit to increase study power' and 'reproducibility' in mouse research

For each of the five recommendations listed below, please grade from 1-to-10 the sentence for clarity, the potential benefit to improve study power & research reproducibility in mouse research, and the 'recommendability'. Mark with an 'X' your grade for each row.	1 (lowest grade, minimal clarity/ minimal expected benefit to improve study reproducibility, I would not recommend it)<br>10 (highest grade, complete clarity/ outstanding benefit and potential, I would absolutely recommend it)										
Five Recommendations	Rows to grade	1	2	3	4	5	6	7	8	9	10
1. On reporting of diet and animal husbandry factors:<br>Use a *paragraph-style template* (here proposed, as non-plagiarism amenable for future meta-analysis) to facilitate the consistent reporting of detailed diet description and husbandry parameters as publishable accompanying 'Supplementary Materials' in future publications, instead of only following reporting checklists (e.g., ARRIVE guidelines).<br>**Suggestions:**	Grade for Clarity										
	Grade for Potential Benefit										
	Would you recommend it										
2. On cage-cage microbiome variability BEFORE experiments:<br>Use a *fecal matter-based microbiome normalization protocol* (e.g., by orally administering a homogenous pool of feces from a group of mice intended for experimentation, to all the mice, prior the beginning of the study) to homogenize the microbial exposure risk across all mice intended for an experiment, and thus reduce cage-cage microbiome variability that naturally occurs in intensive research mouse production/farming.<br>**Suggestions:**	Grade on Clarity										
	Grade on Potential benefit										
	Would you recommend it										
3. On cage dirtiness and time of sampling DURING experiments:<br>*Prevent the uncontrolled accumulation of animal excrements in the cage* by housing a homogeneous number of animals per cage (ideally low density, 1 mouse/cage), or increase frequency of sanitation of cages, *and collect samples 1-2 days after mice have been in clean bedding/cages* (because coprophagia and 'dirty cages' affect the mouse physiology and microbiota).<br>**Suggestions:**	Grade on Clarity										
	Grade on Potential benefit										
	Would you recommend it										
4. On animal density, clusters, and study power:<br>*House one mouse per cage* (unless more mice per cage is scientifically justifiable) and *increase the number of cages per group* (instead of few cages with many mice, because cohousing results in lower study power due to cage clustered correlated data which requires more mice to compensate for study power loss) to maximize the experimental and statistical value of each animal as a test subject during experimentation.<br>**Suggestions:**	Grade on Clarity										
	Grade on Potential benefit										
	Would you recommend it										
5. On Repeating experiments:<br>*Plan and execute statistically powerful designs* to determine the effect of season and reproducibility of results and *do not repeat underpowered* (cage clustered, low sample size) experiments in different seasons (because several unforeseen factors affecting animal husbandry are very difficult to detect and control in diet and personnel).<br>**Suggestions:**	Grade on Clarity										
	Grade on Potential benefit										
	Would you recommend it										

OTHER RECOMMENDATION/S you may think of? Please describe here: ____________________

Supplementary Table 4. Compilation of comments made by individuals that graded the initially drafted five recommendations

1. General comments.

Assistant Professor: The way you are putting this paper is interesting. Good luck with the review.

2. Specific comments for each recommendation.

Notice that no comments were excluded from this compilation. In parenthesis are the scores provided for the Recommendation, in the following order, sentence clarity, Potential beneficial impact on study power and reproducibility, and ‘recommendability’ to other scientists. All graduate research associates were native English speakers.

RECOMMENDATION 1. On reporting of diet and animal husbandry factors:

Use a *paragraph-style template* (here proposed, as non-plagiarism amenable for future meta-analysis) to facilitate the consistent reporting of detailed diet description and husbandry parameters as publishable accompanying ‘Supplementary Materials’ in future publications, instead of only following reporting checklists (e.g., ARRIVE guidelines).

Comments:

- **Professor**: I cannot understand this paragraph, clearly. (Scores provided 5,6,-).
- **Associate Professor**: Clarity - The portion in parentheses regarding (here proposed, as non-plagiarism amenable) …. is not clear to me. (Scores provided 7, 10, 10)
- **Graduate Research Associate**: Diet description might be determined to be an explanation of macronutrient composition, which many studies include rather than diet sterility and storage details. Scores Provided (9,10.10).
- **Assistant Professor**: not clear what (here proposed, as non-plagiarism amenable for future meta-analysis) refers to. (Scores provided, 5, 10, 10)
- **Graduate Research Associate**: Unclear as written – “Report detailed diet and husbandry descriptions within the supplementary materials section of all submitted work. Information should be presented in a paragraph-style template, instead of only following reporting checklists.” (Scores provided, 2,8,8).
- **Associate Professor:** Use a paragraph-style template to facilitate consistent reporting of in vivo study detail such as diet description, husbandry parameters and method of material(s) sterilization. (Suggested example of sentence modification) (Scores provided, 7,10,10).

RECOMMENDATION 2. On cage-cage microbiome variability BEFORE experiments:

Use a *fecal matter-based microbiome normalization protocol* (e.g., by orally administering a homogenous pool of feces from a group of mice intended for experimentation, to all the mice, prior the beginning of the study) to homogenize the microbial exposure risk across all mice intended for an experiment, and thus reduce cage-cage microbiome variability that naturally occurs in intensive research mouse production/farming.

Comments:

- **Associate Professor**: Clarity – are you suggesting that feces from all mice in the experiment be collected at baseline, homogenized, and then a sample be fed orally to all mice in the experiment? How would you ensure that all mice ate the feces sample – an oral gavage? (Scores provided 6,6,6).
- **Associate Professor**: How many samples should be taken for baseline? (Scores provided, 7,10,10).
- **Graduate Research Associate**: Unclear as written and overly verbose. “Prior to study initiation, fecal samples from experimental mice should be collected and processed for oral gavage. Homogenization of the microbiome in this manner reduces cage-to-cage variability.” (Scores provided, 2,8,9).
- **Assistant Professor**: Replace mouse production/farming with ‘settings’. (Scores provided, 9,10,10).
- **Associate Professor:** Not sure “exposure risk” is optimal terming to homogenize microbial exposure across all mice . . . my understanding is that this is a phenomenon not limited to intensive research mouse production/farming but rather occurs with standard mouse housing practices. (Scores provided, 10,7,7).

RECOMMENDATION 3. On cage dirtiness and time of sampling DURING experiments:

Prevent the uncontrolled accumulation of animal excrements in the cage by housing a homogeneous number of animals per cage (ideally low density, 1 mouse/cage), or increase frequency of sanitation of cages, *and collect samples 1-2 days after mice have been in clean bedding/cages* (because coprophagia and ‘dirty cages’ affect the mouse physiology and microbiota).

Comments:

- **Assistant Professor**: I agree to this recommendation. The word “dirtiness” in the title could be [added] considered, e.g. environment, because this recommendation has a potential to normalize not only dirtiness but also other cofounding factors as you mentioned. (Scores provided, 9, 10,10)
- **Professor**: can you collect two or three times a day with a group of mice? (Scores provided 8,8,9).
- **Assistant Professor**: Delete low density because it gets confusing and conflicting with the one below. Not clear if this is one or two recommendations. Needs rewording. (Scores provided, 3, 10, 10).

Supplementary Table 5. Compilation of comments made by individuals that graded the initially drafted five recommendations *(cont.)*

- **Graduate Research Associate**: I think that the last part of the suggestion (collecting samples 1-2 days after cleaning) is a little unclear. Perhaps, try saying “collect samples within a few days of cage changes to avoid the effect of coprophagia on the mouse physiology and microbiota. (Scores provided 7,9,10).
- **Graduate Research Associate:** Ok as written, but I would use less parenthesis. (Scores provided 7,6,5).
- **Assistant Professor:** One mouse per cage may not be economically feasible. (Scores provided 10,5,5).
- **Associate Professor:** Frequency of sanitation requires multifactorial considerations; for some phenotypes, “soiled caging” environments are desirable. The emphasis of timing and cognition of sample collection timing relative to cage change is most important. (Scores provided, 10,7,7).

RECOMMENDATION 4. On animal density, clusters, and study power:

House one mouse per cage (unless more mice per cage is scientifically justifiable) and *increase the number of cages per group* (instead of few cages with many mice, because cohousing results in lower study power due to cage clustered correlated data which requires more mice to compensate for study power loss) to maximize the experimental and statistical value of each animal as a test subject during experimentation.

Comments:

- **Assistant Professor**: I think this point might be difficult to understand for some researchers because people are familiar with the “traditional” way (multiple mice per cage). If there is more space, the more detailed explanation on the rationale of this recommendation would be helpful. (Scores Provided, 9, 10, 10).
- **Associate Professor**: My recommendation to follow this suggestion would be based on calculating cost of individual housing versus the savings in lower number of mice needed for the experiment. (Scores provided. 10, 9, 9).
- **Professor**: put one mouse per cage, IACUC may not approve it. (Scores provided 8, 7, 8).
- **Assistant Professor**: The recommendation is partially same as above, so they both need to be refined. (Scores provided 5,10,10).
- **Associate Professor**: It could make mice more depressive and apatic, that will influence microbiome as well. (Scores provided, 7,7,7).
- **Graduate Research Associate**: I’m not entirely convinced on this point. (Scores provided, 8,3,3)
- **Assistant Professor**: Separate your thoughts here. Sentence seems run on. (Scores provided, 3,5,5).
- **Associate Professor:** Lack of social interactions between animals with one mouse per cage may contribute to the altered hormone levels and increase stress, thus affecting the interaction between the host (e.g., immune system) and the microbiome. (Scores provided, 10,8,7).

RECOMMENDATION 5. On Repeating experiments:

Plan and execute statistically powerful designs to determine the effect of season and reproducibility of results and *do not repeat underpowered* (cage clustered, low sample size) experiments in different seasons (because several unforeseen factors affecting animal husbandry are very difficult to detect and control in diet and personnel).

Comments:

- **Professor**: You need to calculate how many mice are really needed for this experiment. (Scores provided 8,9,8).
- **Graduate Research Associate**: This seems wordy to me…try making it more concise (Scores provided 8,6,7).
- **Graduate Research Associate:** This is all well and good in theory, but in practice it seems unrealistic to wait an entire year between experiments. The first part of the suggestion is a given for most experiments. (Scores provided, 8, 3, 1).
- **Assistant Professor:** Not sure what the point is here. (Scores provided, 2, 1,1).
- **Associate Professor:** Presumably, this is to control for variation in natural product variability (diet, bedding substrate). Alternative if this is truly critical for the particular study design, study interpretation may be use of standardized synthetic or purified materials. This may not be needed and maybe cost prohibitive, an area for further assessment I believe. In table 3, no references are provided for seasonal variation and only variation reported in rats is mentioned. I am concerned about the foundation for the strong recommendation made for this category. (Scores provided, 10,3,1).

Supplementary Table 5. Evidence-based husbandry suggestions to improve study power

Animal Husbandry	Peer-Reviewed Scientific Evidence	Considerations to support relevance of the actionable Recommendations
Diet composition	Short- & long-term changes in diet (type, source; e.g., maintenance vs. breeder chow) induce microbial shifts. Such shifts alter their ability to harvest energy and in turn can affect host systemic immune response.[10, 54]	• Accurate/thorough description of diet composition.<br>• Reporting should include; *i)* diet type, *ii)* diet source, *iii)* batch, *iv)* storage method (frozen, refrigerated, room temperature).
Diet sterility	Autoclaving and irradiation influence external microbial exposure to animals, with non-irradiated diets (*e.g.,* commercially available rodent chow) providing a variable source of bacteria and fungi (see impact of SFB in Table 2).[10, 12] Autoclaving of diet can lead to variations in nutritional composition.	• Every effort should be made to ensure diet sterility.<br>• Thorough description diet sterility (autoclaving, irradiation, none) is required and should include the protocol (*i.e.,,* autoclave settings, irradiation dosage used.
Drinking water	Water source (tap, filtered) and decontamination process (UV light, acidification, autoclaving) alter gut microbiota, specifically via differences in pH, chlorine content, and ion presence. Geographical differences also influence water composition.[50, 53] These factors are animal facility-dependent and almost impossible (and not scientifically correct) to standardize.	• Description of water source/decontamination.<br>• Reporting should include; *i)* method of water treatment (*e.g.,* acidification with HCl, autoclave,), *ii)* the water given by the commercial vendor, *iii)* the source of water, and, *iv)* the final pH.<br>• To minimize bias water bottles should be changed at the same time for all cages.
Coprophagia	Coprophagia is a normal rodent behavior and refers to the consumption of expelled feces present in the cage. Although unavoidable, coprophagia holds strong potential to introduce microbial variability, especially as the degree of cage bedding dirtiness increases (bacterial overgrowth).[1]	• Reducing animal density and increasing cage-changing frequency is advisable.<br>• Prior to each study, a **fecal homogenization protocol** that uses fresh fecal pellets from all mice (rather than soiled cage bedding or mixing of bedding protocols) should be used; cages should be replaced after each inoculation.[55-57]
Fecal collection time	Sampling of feces and intestinal gut microbiota is the most popular method to examine different microbial profiles, but is subject to diurnally host-microbiome changes that bear relevance on experimental outcomes of disease.[58, 59]	• Systematic fecal collection in all mice under consistent conditions (*e.g.,* morning or afternoon) performed 1-2 days after cage changes.[1]<br>• At sacrifice, fecal/other samples should be collected from one mouse per experimental group until all mice have been sampled.
Bedding material	Many varieties of cage bedding are available such as paper products, wood chips and corn cob, each with its advantages and disadvantages in terms of cost, urine absorbency and ammonia build-up.<br>Collectively, evidence indicates that corncob and hardwood bedding hold higher potential of microbial overgrowth to that of paper bedding, although bedding-dependent microbial overgrowth is a factor of cage ventilation (see below), housing density and most importantly, cage change frequency. [12, 60, 61]	• Type of animal bedding should be adequately described and details of related animal husbandry factors (number of mice per cage.<br>• Description of animal husbandry factors that influence microbial overgrowth must accompany.<br>• Diet-microbiome studies should use a non-edible bedding material to avoid bias derived from animals consuming cage bedding (*e.g.,* corn bedding).[62]
Housing density (animals per cage)	Under laboratory conditions, it is common practice to house mice by gender, which contrasts the social organization of wild mice. Within-cage differences (e.g. hormonal stress) may be observed among socially housed mice.[63, 64] Ultimately, the decision to single or group house animals should be based on the type of experiment and assessments (*e.g.,* single caging is advisable in drug safety/efficacy. studies).	• Animal density should be limited to 1-2 mice/cage with animal feces being sampled on day 2 post-cage replacement ('2 × 2 cage sampling rule').[1]<br>• **Single housing** of mice allows for each cage to be treated as a unit for statistical purposes and thus single-cage studies may be more cost-effective since the number of animals can be reduced.[65]
Light/Dark cycle	Maintaining mice in standard light/dark conditions is a generally accepted practice. Disruptions in the circadian rhythm of light/dark cycle have been shown to affect the gut microbiome significantly.	• Description of light/dark conditions, including any disruptions, should be reported.<br>•
Environmental Temperature	Temperature (and humidity, see below) within a cage affects the rate of microbial overgrowth in bedding material and can vary by as much as 5°F to that of the external room temperature. Factors influencing cage temperature include; ***i)*** cage design/material (filter top), ***ii)*** presence/type of bedding, ***iii)*** frequency of bedding replacement, ***iv)*** animal density (including age/sex) and, ***v)*** the presence of forced ventilation.	• Continuous monitoring of cage temperature levels in individual cages should be performed and reported.

Husbandry variables presented according to the ranking of importance by respondents (Figure 4).

Supplementary Table 5. Evidence-based husbandry suggestions to improve study power *(cont.)*.

Animal Husbandry	Evidence	Suggestion
Cage ventilation	Cage ventilation is a factor of air changes per hour [ACPH=(flowmeter reading (in L/min)/cage volume in Litres) x 60min/hr] that largely determines individual cage measurements of ammonia levels, humidity and cage temperature, thus playing an integral role in bedding moisture levels and rate of microbial overgrowth. The interaction between cage ventilation type and bedding material has strong potential to alter intestinal microbial profiles,[1, 60, 66] thereby affecting study reproducibility.	• Description of type of cage ventilation should be reported. Ideally, this should be combined with monitoring cage ammonia, temperature/humidity, which will vary with cage density.<br>• Static isolation caging (without forced ventilation) restricts ventilation, and it is important to compensate by adjusting animal husbandry practices such as cage-change frequency, bedding selection, animal density, placement of cages in a secondary enclosure and macroenvironment adjustments to temperature and humidity.
Bedding soiledness	The degree of bedding soiledness ('dirtiness') is directly related to cage change frequency and animal housing density (see above), and exerts a formidable effect on microbiome bias[1, 67] primarily due to microbial overgrowth in cage bedding and the coprophagic behavior of mice.	See cage change frequency above
Environmental Humidity	Humidity levels can vary from up to 11% higher within an individual cage.[61] Higher humidity levels within a cage (particularly in combination with higher temperature; see above) increase bedding moisture levels, & promote bedding microbial growth.[1, 61, 67]	• Continuous monitoring of individual cage humidity levels could be performed and reported, but it may not be realistic. Instead, reporting should include all factors that contribute to individual cage variability in humidity levels (*e.g.,* number of mice, cage ventilation, cage change frequency, etc.).
Cage type	Bacterial community divergence has been reported concerning different cage types such as barrier access vs open cages.	• Description of type of cage used should be reported
Seasonal variation	Changes to gut microbiome with seasonal variation have been reported in rats.	• Details of seasonal variation both within and across different experiments, should be reported.

Husbandry variables presented according to the ranking of importance by respondents (Figure 4). See further details and discussion in Supplementary Discussion.

Supplementary Table 6. Rationale for six actionable recommendation themes

Rationale for six actionable recommendation themes
**Action theme 1 Rationale.** Our scoping review of literature and the high number of citations for the ARRIVE guidelines (over 3000 times),[68] show that 'checklists' could be a successful approach to improve reporting quality in mouse research. However, with over half a million papers in mouse research, checklists do not ensure that sufficient details are reported, or that methods are reported in a clear, non-misleading fashion and not open to interpretation. We created, as a template, a methods section from three journals deemed by the authors who conducted the literature search to be of good reporting quality (including the J of the AALAS) and the ARRIVE guidelines. The availability of such paragraph would enforce a more uniform transparency, enhance reproducibility, and enable the rapid data mining of future more comprehensive meta-analyses, widely used to help guide the practice of medicine, but scarcely use in basic science. As described in publishing policies, use of the paragraph proposed here should be allowed if authors cite their previous use in their work, and the rationale that lead to their implementation (*i.e.*, this paper) as described under the 'text recycling' section of well-established publisher[69-72] (see **Supplementary discussion**). Reproducibility will occur only if critical study details are provided in published literature.
**Action theme 2 Rationale.** Fecal bacterial profiles can differ widely between cages within a single mouse strain housed under identical conditions. Fecal homogenization, wherein all mice are administered, via oral gavage, a composite of freshly collected feces for 3 days, before the experiment, has been shown as an effective method to minimize inter-cage heterogeneity in gut microbiota.[55-57] Fecal homogenization inoculum should consist only of fresh fecal pellets collected from all experimental mice (rather than soiled cage bedding or mixing of bedding protocols), and all cages should be replaced following each inoculation.[55] The use of cohousing (discussed elsewhere [12]) is a simple approach that that relies on passive transfer of gut microbiota between co-housed animals. Co-housing and is frequently used to assess the influence of complex gut microbiota, treatment effects and has potential to minimize variation between mice within a cage, but the method does not ensure equal dissemination of low-abundance bacteria,[73, 74] or facilitate experiments where males tend to be more aggressive. Although useful in genetic studies, co-housing requires to control for animal density variability and unbalanced clustered designs.
**Action theme 3 Rationale.** Recent culture and microbiome studies of feces and bedding material indicate that increased bedding soiledness ('dirtiness') contributes to period variations in gut microbiome, although the extent to which all potential animal density factor combinations such as body weight, drinking, grinding behavior could ultimately influence gut microbial structure has not yet been examined. Microbiome experiments would benefit if conducted with cages having comparably reduced animal density (*e.g.*, 1-2 mice/cage), with animals being sampled for analysis on day 2 post-cage replacement (*e.g.,* '2×2 cage sampling rule').[1] Mouse biological samples (feces) should be systematically collected at the same time of day (*e.g.*, morning) to avoid diurnal variation.[75]. See further details in **Supplementary Figure 3** and **Supplementary discussion** under 'housing density and cage effect', 'cage frequency and bedding', 'bedding material', and 'time of fecal collection'.
**Action theme 4 Rationale.** Little is known about the impact of season, climate, and latitude in laboratory animal biology, including mouse research. Some studies have shown that the mouse microbiome varies over time in the same facility, but the precise causes of variation are unknown.[13, 76, 77] Seasonal changes to the community composition of gut microbiota [78] could reflect differences in geographic availability to foodstuff suppliers of food ingredients/crops.[79, 80] It is also possible that batch/season variability in diets introduces microbes from distinct locations that are not destroyed by standard irradiation. While it is almost impossible to control for seasonal variation within long-term experiments or multiple experiments spanning over several years, it is crucial to report measures taken to reasonably control for seasonal variation (*e.g.*, food batch storage, inter-experiment fecal homogenization). In certain husbandry settings, the influence of human health and seasonal variation on animal handler microbiome (*e.g.,* respiratory/intestinal infections) could be temporally relevant, but this remains speculative.[81, 82]
**Action theme 5 Rationale:** The Guide requires the social housing of social species, mice, except for where scientific justification for variance is requested. One report describing literature trends and reporting solutions[83] determined that investigators fall into four categories with respect to how they handle clustered data: some ignore clustering, while others either reduce clusters (*e.g.,* cages) to independent observations; use fixed effects regression or ANOVA methods; or explicitly account for clustering in design and methods. Before controlling for cluster effects, investigators must thus determine the extent by which data clustering occurs in their design. We provide the statistical rationale[83] to justify the housing of mice at low or individual densities to promote the most efficient use of animals since grouping of mice in cage clusters have also been shown to increase data variability equally in both males and female mice during experimentation.[84] When rearing mice colonies intended for experimental purposes at large scale, we acknowledge that for technological reasons and space limitations it is cost-effective to use the highest possible densities allowed by the Guide or approved by the local institutional/IACUC boards.
**Action theme 6 Rationale**. Conventional statistical analysis of experimental mouse data considers each mouse as an individual data point irrespective of caging allocation. In the case of experiments where multiple mice are housed in one cage (*e.g.,* 5mice/cage and 10 mice per group), this approach leads to a statistical dependence on clustered data. Data of this kind is referred to as cluster-correlated data. For example, when clustering is a result of cage effect, measurement on units within a single cage (with multiple mice) are more similar than measurements on a cage within the same treatment group. Inappropriate analysis of clustered data can give substantially different results that are not reproducible, particularly when the analysis includes between-mouse treatment. As a general statistical approach, mixed models can be used to account for ICC, which can be retrofitted to compute and monitor the achieved study power.

Supplementary Table 7. Methodology reporting template

***C1. Recommendation 1: Reporting of diet and husbandry factors**: Use of a paragraph-style template (herein provided) to consistently report detailed diet and husbandry factors (e.g., macronutrient, diet sterility) as reproducible publishable accompanying 'Supplementary Materials'.*

**Complete the blanks** [example text in red italics provided] **as appropriate. Specify details for each experimental group. Refer to the ARRIVE guidelines[68] for a complete description of each reporting recommendation.**
This experiment tested groups [*number of experimental mice per group*] of [*age of mice*]-week *old* [*include terms 'age- and/or sex-matched' as applicable*] [*specify gnotobiotic status of mice*] [*mouse strain/species*] mice, housed using [*type of cage(s)*] as [*number of mice per cage*] to provide a study power of [*specify study power calculation and software used*]. Experimental mice were treated with [*specify treatment; drug formulation,* at a dose of [*dose*] administered via [*administration site and route*] and provided with [*specify analgesia/anesthesia and/or specialist equipment (including vendor) used*] for [*treatment duration*] weeks/days. Mice [*were/were not*] randomly allocated to treatment groups.[*specify controls used for each experiment and experimental group*]. Mice were maintained on [*bedding type*] [*specify type of enrichment added to cage*] and fed an [*'autoclaved', 'irradiated', 'non-sterilized'*] [(*specify irradiation dose and/or autoclave settings*)] diet [*diet name and vendor*] [(*kcal, %CHO, %PRO, %FAT/5g*)] and [*'autoclaved', 'irradiated', 'non-sterilized'*], [*tap, filtered, reverse osmosis, etc*] water [*specify water pH*] [*ad libitum*]. All mouse [*cages*], [*food*], [*water*] was replaced every [*X*] days, and a [*fecal homogenization, mixed bedding, co-housing, none*] protocol was used [*time protocol employed*]. Fresh murine feces were collected routinely in the [*morning, afternoon*] from [*specify whether all cages or select cages*] cages [*number of days*] [*before, after, at time of*] cage replacement and stored for [*#days*] at [*temperature, C*] until analysis.<br>***In studies where multiple experiments span over two or more seasons:***<br>Experiments were conducted in [*specify season and year*]. The batch [*specify if single or multiple*] of food/experimental materials [*were/ were not*] used for all experiments. Homogenous colonization of mice spanning across all experiments [*was/was not*] performed **(see Recommendation 4)**. Data analyses were performed in a [*blinded, non-blinded*] manner with [*single animal, group, cage of animals*] considered as an experimental unit.
Example paragraph:
This experiment tested groups (*12 mice/group'*) of *8*-week *old* '*age- and/or sex-matched' germ-free Black 6* mice housed using *ventilated microisolator* cages with an equal number of *2* mice/cage to provide *80% study power*. Age- and sex-matched untreated GF mice were used as negative controls. Experimental mice were administered *2mg/kg dexamethasone treatment via I.P injection each morning for 7 consecutive days, followed by 14-day recovery. No specialist equipment was used.* Mice were randomly allocated to treatment groups. All mice were maintained on *Aspen* bedding (*with nestlet*) and fed *autoclaved (60 minute wet cycle) 40–50 kGy irradiated* (*6/5 irradiated*) pellet food (*PMI Nutrition Int'l., LLC. Labdiet® Charles River. Vac-Pac Rodent*) diet (*5% kcal% fat*) and *autoclaved, irradiated tap* water (*ph 5*) '*ad libitum'*. All mouse *cages*/*food*/*water* was replaced every *7* days. A *fecal homogenization* protocol was used before the start of all experiments. *Fresh* murine feces were collected *routinely, in the morning,* from *all* mice, *2* days *after* cage replacement, and stored for *90* days (*-80C*) until *batch* analysis. Experiments were conducted in *Spring 2019* and *Fall 2020. The same batch of food was used for all experiments.* Data analyses were performed in a *blinded* manner with a *single animal* considered as an experimental unit.

Supplementary Table 8. Summary of actionable items for each recommendation theme

**Action theme 1 on 'Reporting of diet and husbandry factors'**	*Use of a paragraph-style template to report detailed diet and husbandry factors consistently and reproducibly (e.g., macronutrient, diet sterility), publishable as accompanying 'Supplementary Materials"*
	• Determine the aspects required by ARRIVE that apply to your institution, laboratory and experiment.<br>• Use the template provided (**Supplementary Table 7**) and create a standard animal methodology section for your laboratory.<br>• Save customized template on a secure folder. Updates/amendments can be saved as 'version xx'.<br>• Upload your customized template to journals with your manuscripts as supplementary materials.
**Action theme 2 on 'Cage-cage microbiome variability BEFORE mouse experiments'**	*Use of a fecal matter-based microbiome normalization protocol (e.g., by orally administering a homogenous pool of feces from a group of mice intended for experimentation to all the mice at baseline prior to starting the study) to homogenize the microbial exposure risk across all mice intended for an experiment, and thus reduce the cage-cage microbiome variability that naturally occurs as animals age during intensive production of animals for research and experiments.*
	• Determine the number of animals needed and cage density per group, the cage type (e.g., microisolator, static) and bedding material type (*e.g.,* non-edible bedding in diet experiments).<br>• Implement a fecal homogenization; collect fresh feces from all experimental mice and create inoculum to gavage all mice. Repeat for 3 days. Start experiment four days after the final gavage. Detailed protocol previously described.<br>• Collect samples before and after fecal homogenization to confirm microbiota normalization; include in publication.
**Action theme 3 on "Dirty cages' and time of sampling DURING experiments'**	*Prevent the uncontrolled accumulation of animal excrements in the cage, **i)** house a homogeneous number of animals per cage (ideally at low density, 1 mouse/cage), **ii)** adjust frequency of cage sanitation based on animal density, and **iii)** collect samples 1-2 days after mice have been in clean bedding/cages, because coprophagia and 'dirty cages' affect the mouse physiology and microbiota*
	• House an equal number of mice in each cage and maintain a low animal density (see Recommendation 5).<br>• Adjust frequency of cage sanitation (cage changes) to the number of mice per cage (see Figure below)<br>• Systematically collect biological samples (feces) at the same time of day (*e.g.,* morning).<br>• Fresh feces should be collected ideally 2 days after mice placed in clean cages.
**Action theme 4 on 'Repeating experiments in different seasons'**	*Plan and execute statistically powerful designs and do not repeat underpowered (cage clustered, low sample size) experiments in different seasons (because several unforeseen factors affecting animal husbandry are challenging to detect and control for in diet and personnel*
	• *In studies where multiple experiments will span over two or more seasons;*<br>• Obtain sufficient food (from a single batch) to sustain all experimental groups. Store food at -20C or as per manufacturer instructions.<br>• Collect and store fresh frozen feces (-80C with cryo-preserving buffer such as glycerol) from the original, 'first round' of experimental mice for use as a normalizing colonization inoculum in mice of future experimental within study. *Note*: this should be performed in addition to Recommendation 2, point 2.
**Action theme 5 on 'Animal density, clusters, and study power'.**	*House one mouse per cage (unless more mice per cage is scientifically justifiable) and increase the number of cages per group (instead of few cages co-housing many mice which results in cage clustered-correlated data, lower study power and requires more mice to compensate for study power loss) to maximize the experimental and statistical value of each animal as a test subject during experimentation.'*
	• House experimental mice individually housed otherwise scientifically justifiable.<br>• Maintain study power by increasing the total number of mice if implementing higher animal densities (>1 mice/cage) to increase the number of cages per group.<br>• Verify statistical power using open-source study power calculators/software G*power or R.
**Action theme 6 on 'Implementing statistical models to consider ICC in clustered data**	*Use statistical methods designed for analyzing clustered data when multiple mice are housed in one cage, and when data points are obtained from mice over time, to **i)** properly assess treatment effects, **ii)** determine the intraclass correlation coefficient for each study, and then **iii)** to use that information to rapidly generate experiment-specific, customizable study power tables to aid in the assessment, re-/design (if more mice or cages are needed), and reporting of adequately powered studies.'*
	• *In studies where multiple mice are housed per cage';*<br>• Analyze data as cluster-correlated data (and not using methods intended for independent data sets).<br>• Mixed models can be used to account and compute the inter-class correlation coefficient (ICC) (the complete annotated code for the statistical methods is available in the Github code repository)<br>• The computed ICC should be retrofitted in customizable power tables to determine whether more cages per group or more mice per cage are needed to achieve a study power of at least 0.7, ideally 0.8.<br>• As a general statistical approach, mixed models can be used to account for ICC, which can be retrofitted to compute and monitor the achieved study power.

4. Supplementary discussion

To date, there are no perception survey based-studies on the relevance of animal husbandry and variability of microbiome research. Animal models, either SPF or human-associated transplantation models arguably represent a useful platform in microbiome research. However, written and verbal commentaries on the subject might suggest inadvertently that animal husbandry practices introduce sources of confounding,

Perception: The discord between perception in academia and experimental practices highlights the need for appropriate reporting guidelines in this regard in peer-reviewed publications. Unless we implement corrective reporting measures, the lack of information, or misinformation will not self-correct over time. Having an accurate reporting is crucial toward promoting study reproducibility and in understanding diet microbiome interactions and improve current models of disease to benefit patients, but also is critical to prevent the systematic spreading of miscommunication and regain public distrust. The following sections below provide a supplementary evidence-based discussion to support the six actionable Recommendations proposed in this manuscript.

Our collective examination of recent literature in 'mouse microbiome and diet' as a theme illustrates considerable heterogeneity in animal husbandry reporting practices that influence reproducibility in microbiome research, which resembles what happens in the medical literature. [85, 86] From a social perspective, a critical finding derived from the survey was the recognition that academicians from three professional organizations ranked 15 husbandry factors in a reproducible manner, providing for the first time a widely applicable framework to prioritize areas for intervention. Efforts to decrease sources of technical variability could improve the quality of published data with enforcement of reporting guidelines to promote reproducibility, which has been deemed to be strongly confounded.[87] To promote the implementability and adoption of practices to improve research quality, *social incentives* (response to opinion of others as an incentive to lead to change [88]), *immediate reward* (significant findings based on highly powered experiment), and *progress monitoring* (controlling for bias could result in better results to support grants) should be considered by respective institutions.

Suppl on Action theme *1: Reporting of diet and husbandry factors*:

Our data support discussions that journals should continue to prevent at all costs plagiarism in results, but they should be aware that text recycling in methods sections as here proposed has the tremendous potential of increasing study reproducibility in basic and translational research.

Implementation and availability of a template paragraph for rapid and complete husbandry reporting in manuscripts will enable the rapid data mining of future more comprehensive meta-analyses, widely used to help guide the practice of medicine, but scarcely use in basic science. A paragraph and photographs (an image is worth 1000 words) will facilitate the extraction of data in future studies by scientists interested in meta-analysis of basic research. Extracting data for systematic, scoping, or meta-analysis studies is tedious, slow, and prone to errors due to the lack of consistency in reporting strategies. Such inconsistency in reporting, be it in order, natural variation of methods, or lack of information, could also be the result of limited editorial space to publish, and the pressure that exists to avoid plagiarism.

To alleviate concerns with respect to 'text recycling' for 'Method Sections' in publications, here we present the exact excerpt from the BioMed Central publisher available as; http://media.biomedcentral.com/content/editorial/BMC-text-recycling-editorial_guidelines.pdf: "Use of similar or identical phrases in methods sections where there are limited ways to describe a method is not unusual; in fact text recycling may be unavoidable when using a technique that the author has described before, and it may actually be of value when a technique that is common to a number of papers is described. Editors should use their discretion and knowledge of the field when deciding how much text overlap is acceptable in the methods section. An important factor to consider is whether the authors have been transparent, stating that the methods have already been described elsewhere and providing a citation." This statement and our proposition are in line with the interest that exists in the field across

multiple disciplines. For advanced discussions on the topic (July 26th, 2019), please refer to the summary of a meeting hosted by the International Society of Managing & Technical Editors (https://www.ismte.org/) on March 1st, 2019 on the findings of an NSF-sponsored study on text recycling and self-plagiarism.[72, 89]

We propose that to promote reproducibility, studies should follow a basic template where methods sections could be used to support the study and facilitate others to continue and integrate the findings from multiple studies. Of concern, publications in high-tier journals often have limited descriptions of animal husbandry despite growing evidence that multiple husbandry factors serve as sources for confounding. Paraphrasing husbandry baseline practices to avoid infringing on plagiarism is of little value when it results in the omission of crucial details required for study reproducibility. Important methodology components can be described using a paragraph/customizable checklist template. See the rational and discussion below on how the water type and sterility, which vary by vendor[18, 19, 49-53] influence the gut microbiota.

1.1 Type of water and decontamination

The source and decontamination process (UV light, acidification, autoclaving) of the water is often overlooked in mouse studies even though phenotypic gut microbiome-based changes have been reported as a result of the pH level, chlorine content and ion/mineral content of water. [49, 50] Acidification of water (lower pH) is most commonly used across facilities because of its effectiveness in killing gram-negative bacteria (*e.g.,* Pseudomonas spp.) but may also influence phenotypic outcomes of disease. For example, low pH of drinking water has been shown to affect incidence of DM1 and associated gut microbiome differences in non-obese diabetic mice compared to neutral pH.[18, 51] This change was associated with a decrease in *Bacteroides* and *Prevotella* and an increase in *Parabacteroides* when mice switched from acidified drinking water to neutral pH. Acidified water also holds potential to increase heavy metal leakage from water bottles into the drinking water.[52] Chlorination of water has been shown to alter gut microbiome by decreasing *Clostridium perfringens*, *C. difficile*, *Enterobacteriaceae* and *Staphylococcus*, which increased incidence of colon cancer in Apc mice,[19] with other reports showing chlorine content can enhance bacterial antibiotic resistance.[19]

Alternative water sterilization methods, autoclaving and UV sterilization retains much of the original ion content of water which can influence the gut microbiome and murine phenotype including inflammation severity and immune response.[53] It is important to note that mouse suppliers like the Jackson Lab provide acidified drinking water (pH 2.5-3.0 with HCl) for their mice, while other distributors, including Charles River and Taconic, filter and hyper chlorinated water, all of which will influence the microbiome.[53]

Suppl. on Action theme 2: Cage-cage microbiome variability BEFORE mouse experiments

In 2014, our laboratory proposed a fecal homogenization protocol (IsPreFeH) that included use of both fresh mouse feces and bedding material to create the homogenization inoculum.[55] Since then, however, we have discovered a novel source of microbiome bias variability based on microbial overgrowth in bedding material that is depending on degree of cage 'dirtiness'. In light of this discovery, we have amended our original protocol to include only fresh fecal pellets of all mice for the inoculum.

Suppl. on Action theme 3: 'Dirty cages' and time of sampling DURING experiments

3.1 Cage change frequency and bedding 'soiledness' (dirtiness). Sufficient cage change frequency is necessary to maintain animal wellbeing and microenvironmental conditions and should be based on the number and size of animals, cage size, bedding dirtiness and moisture levels (due to organic matter output; urine, feces) and experimental conditions. Institutional protocols for cage changing range from 3 to 7 days, to that of weekly bedding changes with complete sanitation of cages monthly, to once every 14 days (ventilation rates of 60-90 ACH). [67, 90] Although frequent cage changing can have

adverse effects on rodent behavior,[91, 92] consistent and timely cage changing is a prerequisite for sound scientific methodology, especially in microbiome research. Inadequacies in prolonged cage-changing presents a formidable confounding effect on intra-cage ammonia (mucosal membrane irritant),[93] as well as gut microbiome composition and heterogeneity due to microbial proliferation of some (e.g. *Enterobacteriaceae*), but not all bacterial species in cage bedding.[1] Systematic, simultaneous changing of cages based on animal density is advisable (**Supplementary Figure 3**).

3.2 Timing of Fecal Collection. Fecal sampling is the most popular method due to the ease of sampling and non-invasiveness. Samples can also be taken post mortem from along the GI tract (like the cecum), which will yield different densities and compositions of microbiota.[60, 94] However, recent studies now demonstrate that microbiome profiles can fluctuate within as little as 24hrs and that this microbiota diurnal rhythmicity tends to occur between morning and evening within about 20% of the microbial species.[58, 95] Therefore, it is possible that changes in the gut microbiome may be misinterpreted when arbitrarily collected (am vs. pm) throughout an experiment, including at time of sacrifice [60] in that it is advisable to sample intestinal fecal samples systematically across groups (*i.e.,* sacrificing one mouse per experimental group until complete rather than experimental groups 'in batch). Furthermore, diseases that are modeled and studied in mice that affect the proximal GI tract should be sampled more proximally than the feces, which best reflects bacteria of the distal colon.[96]

3.3 Cage Bedding Material. Bedding material is a fundamental component of husbandry. Many varieties are available to laboratory facilities such as paper products, wood chips and corn cob, each with its advantages and disadvantages in terms of cost, urine absorbency and ammonia build-up although the latter is factor of cage type; static vs ventilated [97-99] Corncob is one of the most frequently used bedding materials due to its low cost and high absorbency, but can contribute to discrepancies in feed conversion (mice consume corncob bedding) and can thus interference with diet-microbiome studies.[62] A series of papers have also demonstrated differences in the presence of estrogenic compounds[100] and endotoxin concentrations,[101] between different bedding types, with corncob reported to directly or indirectly alter estrogen signaling and reproductive behavior in mice and rats.[102] For instance, significant differences between Corncob, hardwood and paper bedding in coliform count (corncob highest, paper lowest) and lipopolysaccharide (LSP) levels (hardwood and corncob significantly higher levels compared to paper) may explain mucosal immune response including IgA production.[101, 103]

Of equal importance for consideration is the reported interaction between cage ventilation and various non-edible cage bedding alternatives (paper vs wood chips), which, dependent on the strain and immuno-competence of the animal, has various effects on gut microbiota composition (particularly cecal content).[60] [66] It is possible that interaction between bedding and ventilation reflects a variable presence of bedding-derived, unknown aromatic or volatile compounds (exogenous from bedding material or host-derived; urinary ammonia) and that these are removed from the a ventilated, but not static cage.[60]

3.4 Environmental temperature and humidity. Cage temperature and humidity can be affected by house design/material, presence or type of housing/nesting material, presence of filter top, type of bedding and how often it is changed, number, age, and sex of mice within, and the presence of forced ventilation. Changes in temperature and humidity can disrupt the 'normal' mouse environment, causing stress, inhibition of small intestine motility (downregulating CCK and upregulating VIP), and consequent changes in gut bacteria.[2, 3] Specifically, higher temperature and humidity levels promote inter-cage ammonia levels and microbial overgrowth in cage bedding (increases as cage bedding dirtiness increases), both important elements that can influence experimental outcomes. ***Cage temperature.*** Individual cage temperature has been shown to differ to that of the external environment by as much as 5°F,[61] with fluctuation resulting from the interaction between day and bedding volume.[67] Increased

exposure to cold temperatures can cause adaptive changes in the gut microbiome and brown adipocyte metabolism, making metabolism less efficient and leaking energy in the form of heat.[104, 105] For instance, changes to gut bacteria, including increased *Firmicutes* and *Deferribacteres* and a decrease of *Bacteroidetes* and *Verrucomicrobia*, can be introduced within a couple days of exposure to colder temperatures of 6 degrees Celsius.[104] ***Cage humidity.*** Current guidelines for rodents recommend maintaining a relative microenvironment humidity between 30% to 70%,[106] however significant variations of up to 11% higher have been reported as a result of bedding volume [61] and static mouse caging with levels exceeding 35% can dramatically promote ammonia generation. [67, 90]

3.5 Cage Ventilation. The purpose of ventilation is to provide appropriate air quality and a stable environment. Cage ventilation is a factor of air changes per hour [ACPH= (flowmeter reading (in L/min)/cage volume in Litres) x 60min/hr] that largely determines individual cage measurements of ammonia levels, and humidity and temperature, and thus serves as an important aspect in deciding acceptable cage sanitation intervals. ***Ventilated cages*** typically employ exhaust air recycled into HVAC systems to prevent cross-contamination including airborne animal pathogens of travel of fomites and use a provision of 10-15 fresh air changes/hr as an acceptable guideline. Inlet air velocity but not exhaust design has been shown to affect intra-cage air velocity distribution, with static isolator cages having lower air velocities and higher humidity compared to mechanically ventilated cages.[107] ***Static isolation caging*** (without forced ventilation) restricts ventilation, and it is important to compensate by adjusting animal husbandry practices such as cage-change frequency, bedding selection, animal density, placement of cages in a secondary enclosure and macroenvironment adjustments to temperature and humidity.

Since ventilated cages remove compounds from the air more easily, static cages may differ in the presence of aromatic or volatile compounds like ammonia. Cage ventilation also affects moisture levels in bedding material (results in increased bacterial growth),[1] with the interaction between the type of bedding material (aspen vs paperchip) and cage ventilation (static vs ventilated micro isolator cages) recently shown to modulate the gut microbiota isolated from various intestinal regions,[60] thereby impacting reproducibility of animal models. On the other hand, ventilated cages can be colder or exhibit within-cage temperature disparities, which can lead to murine stress in the mice and gut microbiome changes.[60, 66, 108]

3.6 Cage Type. Divergence in descriptive aspects of bacterial communities (alpha, beta diversity) has been reported for different cage types such as barrier access vs open cages.[109] Type of cage material (*e.g.,* plastic flexible film, plexiglass, polypropylene) and can influence inter-cage temperature and humidity levels.

<u>Suppl. on Action theme 4: Repeating experiments in different seasons.</u>

Changes to the structure and community composition of the gut microbiome with seasonal variation have been reported by our laboratory[77] and by others in rats.[77]. Specifically, in a metagenome-based analysis comparing pool-based sampling of a mouse colony in August which showed a significant association between ileitis with Helicobacter spp. in SAMP1/YitFc vs. control AKR/J mice, which was not observed when the study was repeated 8 months later and sampling individual breeders (Helicobacter was then observed only in control mice).[77] While it is almost impossible to feasibly control for seasonal variation, both within a long-term experiment or that of multiple experiments spanning over several years, it is important to report details of seasonal variation in the methods.

<u>Suppl. on Action theme 5: Animal density, clusters, and study power:</u>

The implementability grades indicate that Recommendation 5 (‘on animal density and individual caging) based on comments and the distribution of the average grades (which are still high and significant

with a t-test p=0.086±0.129 vs. random grades) is likely to continue to be the most heterogeneous form of husbandry methods variability in the literature (**Figure 7B**). The goal of this Recommendation was to investigate the perceptions that exist among leading scientists. Based on external comments, it was clear that this Recommendation elicited the most heterogeneous responses reflecting the resistance that exists among researchers to house 1 MxCg.

The potential for stress is an arguable concern with indivudually housed mice despite that social stress has been equally demonstrated for single- and socially-housed mice.[63, 64] Housing conditions have been shown to modify behavior (aggression, anxiety, distress) and social housing of mice is generally recommended. [106] Under experimental conditions, mice are most commonly housed by gender which is in contrast to the social organization of wild mice,[63, 64] with co-housed males subject to dominance and hierarchy.[64] In a study comparing individual and social housing (C57BL/6, BALB/c) neither the male nor female individually housed mice showed stronger signs of stress compared to that of socially-housed (n=3 mice/cage) mice.[63] However, within-cage differences among socially housed mice (hormonal stress response) were observed.[63] To minimize potential stress in individually housed mice enrichment tools can be used.[110, 111] See details on '*animal well-being*', and '*social housing*' in **Supplementary Discussion** and **Supplementary Table 5**.

4.1 Housing density and cage effect. Previous animal density assessments did not consider effects of husbandry-related environmental effects (noise, lighting differences, diet, hierarchy) or source of animals (vendor) that may affect the microbiota and contribute to 'cage effect' and thus misinterpretation of experimental findings (**Supplementary Figure 3;** accumulation of feces and animal density).[12, 65] Specifically, increasing housing density to increase the number of mice per group is counterintuitive without including a sufficient number of cages per group to avoid 'cage effect'. In addition, the approach of 5 mice/cage is less cost-effective when compared to single housing of mice because of increased need for cage change frequency with higher density cages to minimize bias derived from high organic matter output (see cost calculator). The sections below discuss the primary factors that contribute to cage dirtiness; cage change frequency

4.2 Animal well-being. A recent summary of conclusions of housing density studies (static and ventilated cages) over the last two decades concluded that for most mice strains, a significant decrease in floor space allowance does not negatively impact rodent well-being and thus animals can be kept at twice the recommended levels by the *Guide*.[65] It is important to note however that one study evaluating housing density across 5 inbred mouse strains, reported increased housing density (up to 4 MxCg; 77.4 cm2 for mice between 15 and 25 g) when to significantly and consistently affect certain parameters pertaining to health status, namely kidney weight, adrenal weight, heart rate and percent body fat (increased fat seen in certain mouse strains).[112] Because animal density introduced uncertainty and potential effects ion physiology; it would be easier to conduct balanced experiments (all caged with same number of animals) and low cage animal density.

6. Supplementary References

To identify husbandry factors capable of influencing gut microbiome and study reproducibility we focused on the 172 most recent studies, see references below.[1, 113-284]

1 Rodriguez-Palacios, A. et al. 'Cyclical Bias' in Microbiome Research Revealed by A Portable Germ-Free Housing System Using Nested Isolation. Sci Rep 8, 18, doi:10.1038/s41598-018-20742-1 (2018).

2 Laukens, D., Brinkman, B. M., Raes, J., De Vos, M. & Vandenabeele, P. Heterogeneity of the gut microbiome in mice: guidelines for optimizing experimental design. Fems Microbiol Rev 40, 117-132, doi:10.1093/femsre/fuv036 (2016).

3 Cao, S. G., Wu, W. C., Han, Z. & Wang, M. Y. Effects of psychological stress on small intestinal motility and expression of cholecystokinin and vasoactive intestinal polypeptide in plasma and small intestine in mice. World J Gastroenterol 11, 737-740, doi:10.3748/wjg.v11.i5.737 (2005).

4 Ericsson, A. C. et al. Isolation of segmented filamentous bacteria from complex gut microbiota. Biotechniques 59, 94-98, doi:10.2144/000114319 (2015).

5 Ericsson, A. C., Personett, A. R., Turner, G., Dorfmeyer, R. A. & Franklin, C. L. Variable Colonization after Reciprocal Fecal Microbiota Transfer between Mice with Low and High Richness Microbiota. Front Microbiol 8, 196, doi:10.3389/fmicb.2017.00196 (2017).

6 Barnes, R. H., Kwong, E. & Fiala, G. Decreased Growth Rate Resulting from Prevention of Coprophagy. Fed Proc 22, 125-& (1963).

7 Daft, F. S., Hegner, J. R., Mcdaniel, E. G., Romine, M. K. & Herman, L. G. Role of Coprophagy in Utilization of B Vitamins Synthesized by Intestinal Bacteria. Fed Proc 22, 129-& (1963).

8 Ebino, K. Y., Yoshinaga, K., Saito, T. R. & Takahashi, K. W. A Simple Method for Prevention of Coprophagy in the Mouse. Lab Anim-Uk 22, 1-4, doi:Doi 10.1258/002367788780746548 (1988).

9 Sabbatini, M. E., Pellegrino, N., Rios, M., Blanciotti, L. G. & Vatta, M. S. Variation in exocrine pancreatic secretion in rats due to different commercial diets. Lab Animal 35, 41-49, doi:DOI 10.1038/laban0606-41 (2006).

10 Barnard, D. E., Lewis, S. M., Teter, B. B. & Thigpen, J. E. Open- and Closed-Formula Laboratory Animal Diets and Their Importance to Research. J Am Assoc Lab Anim 48, 709-713 (2009).

11 Thigpen, J. E. et al. Phytoestrogen content of purified, open- and closed-formula laboratory animal diets. Lab Anim Sci 49, 530-536 (1999).

12 Franklin, C. L. & Ericsson, A. C. Microbiota and reproducibility of rodent models. Lab Animal 46, 114-122, doi:10.1038/laban.1222 (2017).

13 Montonye, D. R. et al. Acclimation and Institutionalization of the Mouse Microbiota Following Transportation. Front Microbiol 9, 1085, doi:10.3389/fmicb.2018.01085 (2018).

14 Ericsson, A. C. et al. Effects of vendor and genetic background on the composition of the fecal microbiota of inbred mice. PLoS One 10, e0116704, doi:10.1371/journal.pone.0116704 (2015).

15 Ericsson, A. C., Hagan, C. E., Davis, D. J. & Franklin, C. L. Segmented filamentous bacteria: commensal microbes with potential effects on research. Comp Med 64, 90-98 (2014).

16 Turnbaugh, P. J. et al. The Effect of Diet on the Human Gut Microbiome: A Metagenomic Analysis in Humanized Gnotobiotic Mice. Sci Transl Med 1, doi:ARTN 6ra1410.1126/scitranslmed.3000322 (2009).

17 Jin, U. H. et al. Microbiome-Derived Tryptophan Metabolites and Their Aryl Hydrocarbon Receptor-Dependent Agonist and Antagonist Activities. Mol Pharmacol 85, 777-788, doi:10.1124/mol.113.091165 (2014).

18 Sofi, M. H. et al. pH of Drinking Water Influences the Composition of Gut Microbiome and Type 1 Diabetes Incidence. Diabetes 2014;63:632-644 RESPONSE. Diabetes 64, E20-E21, doi:10.2337/db15-0554 (2015).

19 Sasada, T. et al. Chlorinated Water Modulates the Development of Colorectal Tumors with Chromosomal Instability and Gut Microbiota in Apc-Deficient Mice. Plos One 10, doi:ARTN e013243510.1371/journal.pone.0132435 (2015).
20 Chung, H. C. et al. Gut Immune Maturation Depends on Colonization with a Host-Specific Microbiota. Cell 149, 1578-1593, doi:10.1016/j.cell.2012.04.037 (2012).
21 Stappenbeck, T. S. & Virgin, H. W. Accounting for reciprocal host-microbiome interactions in experimental science. Nature 534, 191-199, doi:10.1038/nature18285 (2016).
22 Biasucci, G. et al. Mode of delivery affects the bacterial community in the newborn gut. Early Hum Dev 86 Suppl 1, 13-15, doi:10.1016/j.earlhumdev.2010.01.004 (2010).
23 Dominguez-Bello, M. G. et al. Delivery mode shapes the acquisition and structure of the initial microbiota across multiple body habitats in newborns. Proc Natl Acad Sci U S A 107, 11971-11975, doi:10.1073/pnas.1002601107 (2010).
24 Korte, S. W., Franklin, C. L., Dorfmeyer, R. A. & Ericsson, A. C. Effects of Fenbendazole-impregnated Feed and Topical Moxidectin during Quarantine on the Gut Microbiota of C57BL/6 Mice. J Am Assoc Lab Anim 57, 229-235 (2018).
25 Lotz, M. et al. Postnatal acquisition of endotoxin tolerance in intestinal epithelial cells. J Exp Med 203, 973-984, doi:10.1084/jem.20050625 (2006).
26 Antonopoulos, D. A. et al. Reproducible community dynamics of the gastrointestinal microbiota following antibiotic perturbation. Infect Immun 77, 2367-2375, doi:10.1128/IAI.01520-08 (2009).
27 Hill, D. A. et al. Metagenomic analyses reveal antibiotic-induced temporal and spatial changes in intestinal microbiota with associated alterations in immune cell homeostasis. Mucosal Immunol 3, 148-158, doi:10.1038/mi.2009.132 (2010).
28 Robinson, C. J. & Young, V. B. Antibiotic administration alters the community structure of the gastrointestinal micobiota. Gut Microbes 1, 279-284, doi:10.4161/gmic.1.4.12614 (2010).
29 Sekirov, I. et al. Antibiotic-induced perturbations of the intestinal microbiota alter host susceptibility to enteric infection. Infect Immun 76, 4726-4736, doi:10.1128/IAI.00319-08 (2008).
30 Hart, M. L., Ericsson, A. C. & Franklin, C. L. Differing Complex Microbiota Alter Disease Severity of the IL-10(-/-) Mouse Model of Inflammatory Bowel Disease. Front Microbiol 8, 792, doi:10.3389/fmicb.2017.00792 (2017).
31 Franklin, C. L. Microbial considerations in genetically engineered mouse research. ILAR J 47, 141-155, doi:10.1093/ilar.47.2.141 (2006).
32 Cominelli, F., Arseneau, K. O., Rodriguez-Palacios, A. & Pizarro, T. T. Uncovering Pathogenic Mechanisms of Inflammatory Bowel Disease Using Mouse Models of Crohn's Disease-Like Ileitis: What is the Right Model? Cell Mol Gastroenterol Hepatol 4, 19-32, doi:10.1016/j.jcmgh.2017.02.010 (2017).
33 Cahill, R. J. et al. Inflammatory bowel disease: an immunity-mediated condition triggered by bacterial infection with Helicobacter hepaticus. Infect Immun 65, 3126-3131 (1997).
34 Hailey, J. R. et al. Impact of Helicobacter hepaticus infection in B6C3F1 mice from twelve National Toxicology Program two-year carcinogenesis studies. Toxicol Pathol 26, 602-611, doi:10.1177/019262339802600503 (1998).
35 Foltz, C. J. et al. Spontaneous inflammatory bowel disease in multiple mutant mouse lines: association with colonization by Helicobacter hepaticus. Helicobacter 3, 69-78 (1998).
36 Chin, E. Y., Dangler, C. A., Fox, J. G. & Schauer, D. B. Helicobacter hepaticus infection triggers inflammatory bowel disease in T cell receptor alphabeta mutant mice. Comp Med 50, 586-594 (2000).
37 Ray, A. et al. Gut Microbial Dysbiosis Due to Helicobacter Drives an Increase in Marginal Zone B Cells in the Absence of IL-10 Signaling in Macrophages. J Immunol 195, 3071-3085, doi:10.4049/jimmunol.1500153 (2015).

38 Alvarado, C. G. et al. Pathogenicity of Helicobacter ganmani in mice susceptible and resistant to infection with H. hepaticus. Comp Med 65, 15-22 (2015).
39 Cook, L. C. et al. The role of estrogen signaling in a mouse model of inflammatory bowel disease: a Helicobacter hepaticus model. PLoS One 9, e94209, doi:10.1371/journal.pone.0094209 (2014).
40 Hillhouse, A. E., Myles, M. H., Taylor, J. F., Bryda, E. C. & Franklin, C. L. Quantitative trait loci in a bacterially induced model of inflammatory bowel disease. Mamm Genome 22, 544-555, doi:10.1007/s00335-011-9343-5 (2011).
41 Livingston, R. S. et al. Pneumocystis carinii infection causes lung lesions historically attributed to rat respiratory virus. Comp Med 61, 45-59 (2011).
42 Lofgren, J. L. et al. Prevalence of murine Helicobacter spp. Infection is reduced by restocking research colonies with Helicobacter-free mice. J Am Assoc Lab Anim Sci 51, 436-442 (2012).
43 Chudnovskiy, A. et al. Host-Protozoan Interactions Protect from Mucosal Infections through Activation of the Inflammasome. Cell 167, 444-456 e414, doi:10.1016/j.cell.2016.08.076 (2016).
44 Escalante, N. K. et al. The common mouse protozoa Tritrichomonas muris alters mucosal T cell homeostasis and colitis susceptibility. J Exp Med 213, 2841-2850, doi:10.1084/jem.20161776 (2016).
45 Tan, T. G. et al. Identifying species of symbiont bacteria from the human gut that, alone, can induce intestinal Th17 cells in mice. Proc Natl Acad Sci U S A 113, E8141-E8150, doi:10.1073/pnas.1617460113 (2016).
46 Besselsen, D. G. et al. Transmission probabilities of mouse parvovirus 1 to sentinel mice chronically exposed to serial dilutions of contaminated bedding. Comp Med 58, 140-144 (2008).
47 Zackular, J. P., Baxter, N. T., Chen, G. Y. & Schloss, P. D. Manipulation of the Gut Microbiota Reveals Role in Colon Tumorigenesis. mSphere 1, doi:10.1128/mSphere.00001-15 (2016).
48 Besselsen, D. G., Franklin, C. L., Livingston, R. S. & Riley, L. K. Lurking in the shadows: emerging rodent infectious diseases. ILAR J 49, 277-290, doi:10.1093/ilar.49.3.277 (2008).
49 Bidot, W. A., Ericsson, A. C. & Franklin, C. L. Effects of water decontamination methods and bedding material on the gut microbiota. Plos One 13, doi:ARTN e019830510.1371/journal.pone.0198305 (2018).
50 Dias, M. F. et al. Changes in mouse gut bacterial community in response to different types of drinking water. Water Res 132, 79-89, doi:10.1016/j.watres.2017.12.052 (2018).
51 Wolf, K. J. et al. Consumption of Acidic Water Alters the Gut Microbiome and Decreases the Risk of Diabetes in NOD Mice. J Histochem Cytochem 62, 237-250, doi:10.1369/0022155413519650 (2014).
52 Nunamaker, E. A., Otto, K. J., Artwohl, J. E. & Fortman, J. D. Leaching of Heavy Metals from Water Bottle Components into the Drinking Water of Rodents. J Am Assoc Lab Anim 52, 22-27 (2013).
53 Barnett, J. A. & Gibson, D. L. H2Oh No! The importance of reporting your water source in your in vivo microbiome studies. Gut Microbes 10, 261-269, doi:10.1080/19490976.2018.1539599 (2019).
54 Ooi, J. H. et al. Dominant effects of the diet on the microbiome and the local and systemic immune response in mice. PLoS One 9, e86366, doi:10.1371/journal.pone.0086366 (2014).
55 Rodriguez-Palacios, A., Aladyshkina, N. & Cominelli, F. Stereomicroscopy and 3D-target myeloperoxidase intestinal phenotyping following a fecal flora homogenization protocol. Protocol Exchange, doi:doi:10.1038/protex.2015.065 (2015).
56 McCafferty, J. M., M.; Gharaibeh, RZ.; Arthur, JC.; Perez-Chanona, E.; Sha, W.; Jobin, C.; Fodor, AA. Stochastic changes over time and not founder effects drive cage effects in microbial community assembly in a mouse model. The ISME Journal volume 7, 2116-2125, doi:10.1038/ismej.2013.106 (2013).
57 Miyoshi, J. et al. Minimizing confounders and increasing data quality in murine models for studies of the gut microbiome. Peerj 6, e5166, doi:10.7717/peerj.5166 (2018).

58 Nobs, S. P., Tuganbaev, T. & Elinav, E. Microbiome diurnal rhythmicity and its impact on host physiology and disease risk. EMBO Rep 20, doi:10.15252/embr.201847129 (2019).
59 Deaver, J. A., Eum, S. Y. & Toborek, M. Circadian Disruption Changes Gut Microbiome Taxa and Functional Gene Composition. Front Microbiol 9, 737, doi:10.3389/fmicb.2018.00737 (2018).
60 Ericsson, A. C. et al. The influence of caging, bedding, and diet on the composition of the microbiota in different regions of the mouse gut. Sci Rep 8, doi:ARTN 406510.1038/s41598-018-21986-7 (2018).
61 Rosenbaum, M. D., VandeWoude, S., Volckens, J. & Johnson, T. Disparities in ammonia, temperature, humidity, and airborne particulate matter between the micro-and macroenvironments of mice in individually ventilated caging. J Am Assoc Lab Anim Sci 49, 177-183 (2010).
62 Ambery, A. G., Tackett, L., Penque, B. A., Hickman, D. L. & Elmendorf, J. S. Effect of Corncob bedding on feed conversion efficiency in a high-fat diet-induced prediabetic model in C57Bl/6J mice. J Am Assoc Lab Anim Sci 53, 449-451 (2014).
63 Arndt, S. S. et al. Individual housing of mice--impact on behaviour and stress responses. Physiol Behav 97, 385-393, doi:10.1016/j.physbeh.2009.03.008 (2009).
64 Bartolomucci, A. et al. Social factors and individual vulnerability to chronic stress exposure. Neurosci Biobehav Rev 29, 67-81, doi:10.1016/j.neubiorev.2004.06.009 (2005).
65 Svenson, K. L. & Paigen, B. Recommended housing densities for research mice: filling the gap in data-driven alternatives. Faseb J 33, 3097-3111, doi:10.1096/fj.201801972R (2019).
66 Thoene-Reineke, C. et al. Composition of Intestinal Microbiota in Immune-Deficient Mice Kept in Three Different Housing Conditions. Plos One 9, doi:ARTN e11340610.1371/journal.pone.0113406 (2014).
67 Rosenbaum, M. D., VandeWoude, S. & Johnson, T. E. Effects of Cage-Change Frequency and Bedding Volume on Mice and Their Microenvironment. J Am Assoc Lab Anim 48, 763-773 (2009).
68 Kilkenny, C., Browne, W. J., Cuthill, I. C., Emerson, M. & Altman, D. G. Improving bioscience research reporting: the ARRIVE guidelines for reporting animal research. PLoS Biol 8, e1000412, doi:10.1371/journal.pbio.1000412 (2010).
69 Harriman, S. & Patel, J. Text recycling: acceptable or misconduct? BMC Med 12, 148, doi:10.1186/s12916-014-0148-8 (2014).
70 Burdine, L. K., de Castro Maymone, M. B. & Vashi, N. A. Text recycling: Self-plagiarism in scientific writing. Int J Womens Dermatol 5, 134-136, doi:10.1016/j.ijwd.2018.10.002 (2019).
71 Trim, C. M. & Axiak Flammer, S. M. Managing text-recycling: an ongoing issue. Vet Anaesth Analg 44, 695-696, doi:10.1016/j.vaa.2017.08.001 (2017).
72 Barham, K. ISMTE Recap: Text recycling and self-plagarism in academic publishing
73 Velazquez, E. M. et al. Endogenous Enterobacteriaceae underlie variation in susceptibility to Salmonella infection. Nat Microbiol 4, 1057-1064, doi:10.1038/s41564-019-0407-8 (2019).
74 Robertson, S. J. et al. Comparison of Co-housing and Littermate Methods for Microbiota Standardization in Mouse Models. Cell Rep 27, 1910-1919 e1912, doi:10.1016/j.celrep.2019.04.023 (2019).
75 Leone, V. et al. Effects of diurnal variation of gut microbes and high-fat feeding on host circadian clock function and metabolism. Cell Host Microbe 17, 681-689, doi:10.1016/j.chom.2015.03.006 (2015).
76 Ericsson, A. C. & Franklin, C. L. Manipulating the Gut Microbiota: Methods and Challenges. ILAR J 56, 205-217, doi:10.1093/ilar/ilv021 (2015).
77 Rodriguez-Palacios, A. et al. The Artificial Sweetener Splenda Promotes Gut Proteobacteria, Dysbiosis, and Myeloperoxidase Reactivity in Crohn's Disease-Like Ileitis. Inflamm Bowel Dis 24, 1005-1020, doi:10.1093/ibd/izy060 (2018).
78 Liu, J. et al. Seasonal Variation Influences on Intestinal Microbiota in Rats. Curr Microbiol 75, 1006-1010, doi:10.1007/s00284-018-1480-6 (2018).

79 Heindel, J. J. & vom Saal, F. S. Meeting report: batch-to-batch variability in estrogenic activity in commercial animal diets--importance and approaches for laboratory animal research. Environ Health Perspect 116, 389-393, doi:10.1289/ehp.10524 (2008).
80 Moraal, M. et al. The influence of food restriction versus ad libitum feeding of chow and purified diets on variation in body weight, growth and physiology of female Wistar rats. Lab Anim 46, 101-107, doi:10.1258/la.2011.011011 (2012).
81 Sonnenberg, A. Seasonal variation of enteric infections and inflammatory bowel disease. Inflamm Bowel Dis 15, 809, doi:10.1002/ibd.20770 (2009).
82 Sonnenberg, A. Seasonal variation of enteric infections and inflammatory bowel disease. Inflamm Bowel Dis 14, 955-959, doi:10.1002/ibd.20408 (2008).
83 Galbraith, S., Daniel, J. A. & Vissel, B. A study of clustered data and approaches to its analysis. J Neurosci 30, 10601-10608, doi:10.1523/JNEUROSCI.0362-10.2010 (2010).
84 Prendergast, B. J., Onishi, K. G. & Zucker, I. Female mice liberated for inclusion in neuroscience and biomedical research. Neurosci Biobehav Rev 40, 1-5, doi:10.1016/j.neubiorev.2014.01.001 (2014).
85 Bramhall, M., Florez-Vargas, O., Stevens, R., Brass, A. & Cruickshank, S. Quality of methods reporting in animal models of colitis. Inflamm Bowel Dis 21, 1248-1259, doi:10.1097/MIB.0000000000000369 (2015).
86 Jin, Y. et al. Does the medical literature remain inadequately described despite having reporting guidelines for 21 years? - A systematic review of reviews: an update. J Multidiscip Healthc 11, 495-510, doi:10.2147/JMDH.S155103 (2018).
87 Ioannidis, J. P. A. Why most published research findings are false. Plos Med 2, 696-701, doi:ARTN e12410.1371/journal.pmed.0020124 (2005).
88 Edelson, M., Sharot, T., Dolan, R. J. & Dudai, Y. Following the crowd: brain substrates of long-term memory conformity. Science 333, 108-111, doi:10.1126/science.1203557 (2011).
89 Text Recycling and Self-plagiarism: Findings from an NSF-sponsored study and implications for Journal Offices, https://www.ismte.org/events/EventDetails.aspx?id=1209792&hhSearchTerms=%22text+and+recycling%22.
90 Reeb-Whitaker, C. K. et al. The impact of reduced frequency of cage changes on the health of mice housed in ventilated cages. Lab Anim-Uk 35, 58-73, doi:Doi 10.1258/0023677011911381 (2001).
91 Burn, C. C., Peters, A., Day, M. J. & Mason, G. J. Long-term effects of cage-cleaning frequency and bedding type on laboratory rat health, welfare, and handleability: a cross-laboratory study. Lab Anim 40, 353-370, doi:10.1258/002367706778476460 (2006).
92 Beynen, A. C. & van Tintelen, G. Daily change of cage depresses mass gain in mice. Z Versuchstierkd 33, 106-107 (1990).
93 Perkins, S. E. & Lipman, N. S. Characterization and quantification of microenvironmental contaminants in isolator cages with a variety of contact beddings. Contemp Top Lab Anim Sci 34, 93-98 (1995).
94 Pang, W., Vogensen, F. K., Nielsen, D. S. & Hansen, A. K. Faecal and caecal microbiota profiles of mice do not cluster in the same way. Lab Anim 46, 231-236, doi:10.1258/la.2012.011128 (2012).
95 Thaiss, C. A. et al. Microbiota Diurnal Rhythmicity Programs Host Transcriptome Oscillations. Cell 167, 1495-1510 e1412, doi:10.1016/j.cell.2016.11.003 (2016).
96 Turner, P. V. The role of the gut microbiota on animal model reproducibility. Animal Model Exp Med 1, 109-115, doi:10.1002/ame2.12022 (2018).
97 Smith, E., Stockwell, J. D., Schweitzer, I., Langley, S. H. & Smith, A. L. Evaluation of cage micro-environment of mice housed on various types of bedding materials. Contemp Top Lab Anim 43, 12-17 (2004).
98 Institute for Laboratory Animal Research. 8 edn, (National Academies Press, 2011).

99 Ferrecchia, C. E., Jensen, K. & Van Andel, R. Intracage Ammonia Levels in Static and Individually Ventilated Cages Housing C57BL/6 Mice on 4 Bedding Substrates. J Am Assoc Lab Anim 53, 146-151 (2014).
100 Lorand, T., Vigh, E. & Garai, J. Hormonal Action of Plant Derived and Anthropogenic Non-Steroidal Estrogenic Compounds: Phytoestrogens and Xenoestrogens. Curr Med Chem 17, 3542-3574, doi:Doi 10.2174/092986710792927813 (2010).
101 Whiteside, T. E., Thigpen, J. E., Kissling, G. E., Grant, M. G. & Forsythe, D. B. Endotoxin, Coliform, and Dust Levels in Various Types of Rodent Bedding. J Am Assoc Lab Anim 49, 184-189 (2010).
102 Landeros, R. V. et al. Corncob Bedding Alters the Effects of Estrogens on Aggressive Behavior and Reduces Estrogen Receptor-alpha Expression in the Brain. Endocrinology 153, 949-953, doi:10.1210/en.2011-1745 (2012).
103 Ewaldsson, B., Fogelmark, B., Feinstein, R., Ewaldsson, L. & Rylander, R. Microbial cell wall product contamination of bedding may induce pulmonary inflammation in rats. Lab Anim-Uk 36, 282-290, doi:Doi 10.1258/002367702320162397 (2002).
104 Zietak, M., Chabowska-Kita, A. & Kozak, L. P. Brown fat thermogenesis: Stability of developmental programming and transient effects of temperature and gut microbiota in adults. Biochimie 134, 93-98, doi:10.1016/j.biochi.2016.12.006 (2017).
105 Chevalier, C. et al. Gut Microbiota Orchestrates Energy Homeostasis during Cold. Cell 163, 1360-1374, doi:10.1016/j.cell.2015.11.004 (2015).
106 Industries, D. o. P. Code of Practice for the Housing and Care of Laboratory Mice, Rats, Guinea Pigs and Rabbits. (Victorian Government Department of Primary Industries, 2004).
107 Memarzadeh, F., Harrison, P. C., Riskowski, G. L. & Henze, T. Comparison of environment and mice in static and mechanically ventilated isolator cages with different air velocities and ventilation designs. Contemp Top Lab Anim 43, 14-20 (2004).
108 David, J. M., Knowles, S., Lamkin, D. M. & Stout, D. B. Individually Ventilated Cages Impose Cold Stress on Laboratory Mice: A Source of Systemic Experimental Variability. J Am Assoc Lab Anim 52, 738-744 (2013).
109 Rausch, P. et al. Analysis of factors contributing to variation in the C57BL/6J fecal microbiota across German animal facilities. Int J Med Microbiol 306, 343-355, doi:10.1016/j.ijmm.2016.03.004 (2016).
110 Jirkof, P. Effects of experimental housing conditions on recovery of laboratory mice. Lab Anim (NY) 44, 65-70, doi:10.1038/laban.662 (2015).
111 Xie, H. et al. Enrichment-induced exercise to quantify the effect of different housing conditions: a tool to standardize enriched environment protocols. Behav Brain Res 249, 81-89, doi:10.1016/j.bbr.2013.04.032 (2013).
112 Morgan, J. L. et al. Effects of Housing Density in Five Inbred Strains of Mice. Plos One 9, doi:ARTN e9001210.1371/journal.pone.0090012 (2014).
113 Chen, K. et al. Preventive Effects and Mechanisms of Garlic on Dyslipidemia and Gut Microbiome Dysbiosis. Nutrients 11, doi:10.3390/nu11061225 (2019).
114 Cowardin, C. A. et al. Mechanisms by which sialylated milk oligosaccharides impact bone biology in a gnotobiotic mouse model of infant undernutrition. Proc Natl Acad Sci U S A, doi:10.1073/pnas.1821770116 (2019).
115 Zhang, Z. et al. Effects of a lactulose-rich diet on fecal microbiome and metabolome in pregnant mice. J Agric Food Chem, doi:10.1021/acs.jafc.9b01479 (2019).
116 Kim, H. et al. Persistent changes in liver methylation and microbiome composition following reversal of diet-induced non-alcoholic-fatty liver disease. Cell Mol Life Sci, doi:10.1007/s00018-019-03114-4 (2019).
117 Yuan, T. et al. ApoE-Dependent Protective Effects of Sesamol on High-Fat Diet-Induced Behavioral Disorders: Regulation of the Microbiome-Gut-Brain Axis. J Agric Food Chem, doi:10.1021/acs.jafc.9b01436 (2019).

118 Shikano, A. et al. Effects of Lactobacillus plantarum Uruma-SU4 fermented green loofah on plasma lipid levels and gut microbiome of high-fat diet fed mice. Food Res Int 121, 817-824, doi:10.1016/j.foodres.2018.12.065 (2019).
119 Boehme, M. et al. Mid-life microbiota crises: middle age is associated with pervasive neuroimmune alterations that are reversed by targeting the gut microbiome. Mol Psychiatry, doi:10.1038/s41380-019-0425-1 (2019).
120 Rasmussen, T. S. et al. Mouse Vendor Influence on the Bacterial and Viral Gut Composition Exceeds the Effect of Diet. Viruses 11, doi:10.3390/v11050435 (2019).
121 Vitaglione, P. et al. Coffee prevents fatty liver disease induced by a high-fat diet by modulating pathways of the gut-liver axis. J Nutr Sci 8, e15, doi:10.1017/jns.2019.10 (2019).
122 Neuman, V. et al. Human gut microbiota transferred to germ-free NOD mice modulate the progression towards type 1 diabetes regardless of the pace of beta cell function loss in the donor. Diabetologia, doi:10.1007/s00125-019-4869-2 (2019).
123 Li, Z. et al. The Gut Microbiome on a Periodized Low-Protein Diet Is Associated With Improved Metabolic Health. Front Microbiol 10, 709, doi:10.3389/fmicb.2019.00709 (2019).
124 Liu, Y., Wu, X. & Jiang, H. High dietary fat intake lowers serum equol concentration and promotes prostate carcinogenesis in a transgenic mouse prostate model. Nutr Metab (Lond) 16, 24, doi:10.1186/s12986-019-0351-x (2019).
125 Wollam, J. et al. The Microbiota-Produced N-Formyl Peptide fMLF Promotes Obesity-Induced Glucose Intolerance. Diabetes, doi:10.2337/db18-1307 (2019).
126 Nerurkar, P. V., Orias, D., Soares, N., Kumar, M. & Nerurkar, V. R. Momordica charantia (bitter melon) modulates adipose tissue inflammasome gene expression and adipose-gut inflammatory cross talk in high-fat diet (HFD)-fed mice. J Nutr Biochem 68, 16-32, doi:10.1016/j.jnutbio.2019.03.003 (2019).
127 Munch, N. S. et al. High-fat Diet Accelerates Carcinogenesis in a Mouse Model of Barrett's Esophagus via IL8 and Alterations to the Gut Microbiome. Gastroenterology, doi:10.1053/j.gastro.2019.04.013 (2019).
128 Bang, S. J. et al. Effect of raw potato starch on the gut microbiome and metabolome in mice. Int J Biol Macromol 133, 37-43, doi:10.1016/j.ijbiomac.2019.04.085 (2019).
129 Poole, A. C. et al. Human Salivary Amylase Gene Copy Number Impacts Oral and Gut Microbiomes. Cell Host Microbe 25, 553-564 e557, doi:10.1016/j.chom.2019.03.001 (2019).
130 Singh, K. et al. Dietary Arginine Regulates Severity of Experimental Colitis and Affects the Colonic Microbiome. Front Cell Infect Microbiol 9, 66, doi:10.3389/fcimb.2019.00066 (2019).
131 Liao, X. et al. Alteration of gut microbiota induced by DPP-4i treatment improves glucose homeostasis. EBioMedicine, doi:10.1016/j.ebiom.2019.03.057 (2019).
132 Kovatcheva-Datchary, P. et al. Simplified Intestinal Microbiota to Study Microbe-Diet-Host Interactions in a Mouse Model. Cell Rep 26, 3772-3783 e3776, doi:10.1016/j.celrep.2019.02.090 (2019).
133 Tauzin, A. S. et al. Sucrose 6(F)-phosphate phosphorylase: a novel insight in the human gut microbiome. Microb Genom 5, doi:10.1099/mgen.0.000253 (2019).
134 Wang, W. et al. Optimal Dietary Ferulic Acid for Suppressing the Obesity-Related Disorders in Leptin-Deficient Obese C57BL/6J -ob/ob Mice. J Agric Food Chem 67, 4250-4258, doi:10.1021/acs.jafc.8b06760 (2019).
135 Tran, T. T. T. et al. Prebiotic supplementation in frail older people affects specific gut microbiota taxa but not global diversity. Microbiome 7, 39, doi:10.1186/s40168-019-0654-1 (2019).
136 Liu, T. et al. A More Robust Gut Microbiota in Calorie-Restricted Mice Is Associated with Attenuated Intestinal Injury Caused by the Chemotherapy Drug Cyclophosphamide. Mbio 10, doi:10.1128/mBio.02903-18 (2019).

137 Larsen, I. S. et al. Human Paneth cell alpha-defensin 5 treatment reverses dyslipidemia and improves glucoregulatory capacity in diet-induced obese mice. Am J Physiol Endocrinol Metab, doi:10.1152/ajpendo.00019.2019 (2019).
138 Bernard, A. et al. A Preventive Prebiotic Supplementation Improves the Sweet Taste Perception in Diet-Induced Obese Mice. Nutrients 11, doi:10.3390/nu11030549 (2019).
139 Rangan, P. et al. Fasting-Mimicking Diet Modulates Microbiota and Promotes Intestinal Regeneration to Reduce Inflammatory Bowel Disease Pathology. Cell Rep 26, 2704-2719 e2706, doi:10.1016/j.celrep.2019.02.019 (2019).
140 Ahmadi, S. et al. Prebiotics from acorn and sago prevent high-fat-diet-induced insulin resistance via microbiome-gut-brain axis modulation. J Nutr Biochem 67, 1-13, doi:10.1016/j.jnutbio.2019.01.011 (2019).
141 Vidal-Lletjos, S. et al. Dietary Protein Intake Level Modulates Mucosal Healing and Mucosa-Adherent Microbiota in Mouse Model of Colitis. Nutrients 11, doi:10.3390/nu11030514 (2019).
142 Caro-Gomez, E. et al. Green Coffee Extract Improves Cardiometabolic Parameters and Modulates Gut Microbiota in High-Fat-Diet-Fed ApoE(-/-) Mice. Nutrients 11, doi:10.3390/nu11030497 (2019).
143 Wang, X. X. et al. Amelioration of Growth Performance, Lipid Accumulation, and Intestinal Health in Mice by a Cooked Mixture of Lean Meat and Resistant Starch. Mol Nutr Food Res 63, e1801364, doi:10.1002/mnfr.201801364 (2019).
144 Wu, Y. et al. Inhibition of Tumor Growth by Dietary Indole-3-Carbinol in a Prostate Cancer Xenograft Model May Be Associated with Disrupted Gut Microbial Interactions. Nutrients 11, doi:10.3390/nu11020467 (2019).
145 Caminero, A. et al. Lactobacilli Degrade Wheat Amylase Trypsin Inhibitors to Reduce Intestinal Dysfunction Induced by Immunogenic Wheat Proteins. Gastroenterology 156, 2266-2280, doi:10.1053/j.gastro.2019.02.028 (2019).
146 La Rosa, S. L. et al. The human gut Firmicute Roseburia intestinalis is a primary degrader of dietary beta-mannans. Nat Commun 10, 905, doi:10.1038/s41467-019-08812-y (2019).
147 Ke, X. et al. Synbiotic-driven improvement of metabolic disturbances is associated with changes in the gut microbiome in diet-induced obese mice. Mol Metab 22, 96-109, doi:10.1016/j.molmet.2019.01.012 (2019).
148 Manuel, C. R., Latuga, M. S., Ashby, C. R., Jr. & Reznik, S. E. Immune tolerance attenuates gut dysbiosis, dysregulated uterine gene expression and high-fat diet potentiated preterm birth in mice. Am J Obstet Gynecol 220, 596 e591-596 e528, doi:10.1016/j.ajog.2019.02.028 (2019).
149 Abulizi, N. et al. Gut Mucosal Proteins and Bacteriome Are Shaped by the Saturation Index of Dietary Lipids. Nutrients 11, doi:10.3390/nu11020418 (2019).
150 van den Elsen, L. W. J. et al. Prebiotic oligosaccharides in early life alter gut microbiome development in male mice while supporting influenza vaccination responses. Benef Microbes 10, 279-291, doi:10.3920/BM2018.0098 (2019).
151 Petersen, C. et al. Dietary supplementation with strawberry induces marked changes in the composition and functional potential of the gut microbiome in diabetic mice. J Nutr Biochem 66, 63-69, doi:10.1016/j.jnutbio.2019.01.004 (2019).
152 Hussain, M. et al. High dietary fat intake induces a microbiota signature that promotes food allergy. J Allergy Clin Immunol, doi:10.1016/j.jaci.2019.01.043 (2019).
153 Kain, V. et al. Obesogenic diet in aging mice disrupts gut microbe composition and alters neutrophil:lymphocyte ratio, leading to inflamed milieu in acute heart failure. Faseb J 33, 6456-6469, doi:10.1096/fj.201802477R (2019).
154 Laudisi, F. et al. The Food Additive Maltodextrin Promotes Endoplasmic Reticulum Stress-Driven Mucus Depletion and Exacerbates Intestinal Inflammation. Cell Mol Gastroenterol Hepatol 7, 457-473, doi:10.1016/j.jcmgh.2018.09.002 (2019).

155 Gu, J. et al. Dietary Black Raspberries Impact the Colonic Microbiome and Phytochemical Metabolites in Mice. Mol Nutr Food Res 63, e1800636, doi:10.1002/mnfr.201800636 (2019).
156 Xu, J. et al. Jamun (Eugenia jambolana Lam.) Fruit Extract Prevents Obesity by Modulating the Gut Microbiome in High-Fat-Diet-Fed Mice. Mol Nutr Food Res 63, e1801307, doi:10.1002/mnfr.201801307 (2019).
157 Liang, Y. et al. Organophosphorus pesticide chlorpyrifos intake promotes obesity and insulin resistance through impacting gut and gut microbiota. Microbiome 7, 19, doi:10.1186/s40168-019-0635-4 (2019).
158 Cheng, N., Chen, S., Liu, X., Zhao, H. & Cao, W. Impact of SchisandraChinensis Bee Pollen on Nonalcoholic Fatty Liver Disease and Gut Microbiota in HighFat Diet Induced Obese Mice. Nutrients 11, doi:10.3390/nu11020346 (2019).
159 Baxter, N. T., Lesniak, N. A., Sinani, H., Schloss, P. D. & Koropatkin, N. M. The Glucoamylase Inhibitor Acarbose Has a Diet-Dependent and Reversible Effect on the Murine Gut Microbiome. mSphere 4, doi:10.1128/mSphere.00528-18 (2019).
160 Wang, P. et al. Resveratrol-induced gut microbiota reduces obesity in high-fat diet-fed mice. Int J Obes (Lond), doi:10.1038/s41366-019-0332-1 (2019).
161 Wankhade, U. D. et al. Sex-Specific Changes in Gut Microbiome Composition following Blueberry Consumption in C57BL/6J Mice. Nutrients 11, doi:10.3390/nu11020313 (2019).
162 Tousen, Y. et al. Resistant Starch Attenuates Bone Loss in Ovariectomised Mice by Regulating the Intestinal Microbiota and Bone-Marrow Inflammation. Nutrients 11, doi:10.3390/nu11020297 (2019).
163 Zinno, P. et al. Supplementation with dairy matrices impacts on homocysteine levels and gut microbiota composition of hyperhomocysteinemic mice. Eur J Nutr, doi:10.1007/s00394-019-01911-y (2019).
164 Miller, A. W., Orr, T., Dearing, D. & Monga, M. Loss of function dysbiosis associated with antibiotics and high fat, high sugar diet. ISME J 13, 1379-1390, doi:10.1038/s41396-019-0357-4 (2019).
165 Everard, A. et al. Intestinal epithelial N-acylphosphatidylethanolamine phospholipase D links dietary fat to metabolic adaptations in obesity and steatosis. Nat Commun 10, 457, doi:10.1038/s41467-018-08051-7 (2019).
166 Okouchi, R. et al. Simultaneous Intake of Euglena gracilis and Vegetables Exerts Synergistic Anti-Obesity and Anti-Inflammatory Effects by Modulating the Gut Microbiota in Diet-Induced Obese Mice. Nutrients 11, doi:10.3390/nu11010204 (2019).
167 Chen, T. et al. Green Tea Polyphenols Modify the Gut Microbiome in db/db Mice as Co-Abundance Groups Correlating with the Blood Glucose Lowering Effect. Mol Nutr Food Res 63, e1801064, doi:10.1002/mnfr.201801064 (2019).
168 Ntemiri, A. et al. Retention of Microbiota Diversity by Lactose-Free Milk in a Mouse Model of Elderly Gut Microbiota. J Agric Food Chem 67, 2098-2112, doi:10.1021/acs.jafc.8b06414 (2019).
169 Las Heras, V. et al. Short-term consumption of a high-fat diet increases host susceptibility to Listeria monocytogenes infection. Microbiome 7, 7, doi:10.1186/s40168-019-0621-x (2019).
170 Schneider, K. M. et al. Intestinal Microbiota Protects against MCD Diet-Induced Steatohepatitis. Int J Mol Sci 20, doi:10.3390/ijms20020308 (2019).
171 Alves da Silva, A. V. et al. Murine Methyl Donor Deficiency Impairs Early Growth in Association with Dysmorphic Small Intestinal Crypts and Reduced Gut Microbial Community Diversity. Curr Dev Nutr 3, nzy070, doi:10.1093/cdn/nzy070 (2019).
172 Ribeiro, F. M. et al. Limited Effects of Low-to-Moderate Aerobic Exercise on the Gut Microbiota of Mice Subjected to a High-Fat Diet. Nutrients 11, doi:10.3390/nu11010149 (2019).
173 Pak, H. H. et al. The Metabolic Response to a Low Amino Acid Diet is Independent of Diet-Induced Shifts in the Composition of the Gut Microbiome. Sci Rep 9, 67, doi:10.1038/s41598-018-37177-3 (2019).

174 Wu, C. et al. Asperlin Stimulates Energy Expenditure and Modulates Gut Microbiota in HFD-Fed Mice. Mar Drugs 17, doi:10.3390/md17010038 (2019).
175 Tanabe, K. et al. Dietary Fructooligosaccharide and Glucomannan Alter Gut Microbiota and Improve Bone Metabolism in Senescence-Accelerated Mouse. J Agric Food Chem 67, 867-874, doi:10.1021/acs.jafc.8b05164 (2019).
176 Wang, D. et al. Effects of triphenyl phosphate exposure during fetal development on obesity and metabolic dysfunctions in adult mice: Impaired lipid metabolism and intestinal dysbiosis. Environ Pollut 246, 630-638, doi:10.1016/j.envpol.2018.12.053 (2019).
177 Warda, A. K. et al. Heat-killed lactobacilli alter both microbiota composition and behaviour. Behav Brain Res 362, 213-223, doi:10.1016/j.bbr.2018.12.047 (2019).
178 Qu, L. et al. Kiwifruit seed oil prevents obesity by regulating inflammation, thermogenesis, and gut microbiota in high-fat diet-induced obese C57BL/6 mice. Food Chem Toxicol 125, 85-94, doi:10.1016/j.fct.2018.12.046 (2019).
179 Raza, G. S. et al. Hypocholesterolemic Effect of the Lignin-Rich Insoluble Residue of Brewer's Spent Grain in Mice Fed a High-Fat Diet. J Agric Food Chem 67, 1104-1114, doi:10.1021/acs.jafc.8b05770 (2019).
180 Li, A. et al. The impact of Bacillus subtilis 18 isolated from Tibetan yaks on growth performance and gut microbial community in mice. Microb Pathog 128, 153-161, doi:10.1016/j.micpath.2018.12.031 (2019).
181 Zegarra-Ruiz, D. F. et al. A Diet-Sensitive Commensal Lactobacillus Strain Mediates TLR7-Dependent Systemic Autoimmunity. Cell Host Microbe 25, 113-127 e116, doi:10.1016/j.chom.2018.11.009 (2019).
182 Lin, G. et al. Role of Green Macroalgae Enteromorpha Prolifera Polyphenols in the Modulation of Gene Expression and Intestinal Microflora Profiles in Type 2 Diabetic Mice. Int J Mol Sci 20, doi:10.3390/ijms20010025 (2018).
183 Gart, E. et al. Diet-Independent Correlations between Bacteria and Dysfunction of Gut, Adipose Tissue, and Liver: A Comprehensive Microbiota Analysis in Feces and Mucosa of the Ileum and Colon in Obese Mice with NAFLD. Int J Mol Sci 20, doi:10.3390/ijms20010001 (2018).
184 Yan, X. et al. Antidiabetic Potential of Green Seaweed Enteromorpha prolifera Flavonoids Regulating Insulin Signaling Pathway and Gut Microbiota in Type 2 Diabetic Mice. J Food Sci 84, 165-173, doi:10.1111/1750-3841.14415 (2019).
185 Yang, B., Ye, C., Yan, B., He, X. & Xing, K. Assessing the Influence of Dietary History on Gut Microbiota. Curr Microbiol 76, 237-247, doi:10.1007/s00284-018-1616-8 (2019).
186 Liao, W. et al. Resveratrol-Induced White Adipose Tissue Browning in Obese Mice by Remodeling Fecal Microbiota. Molecules 23, doi:10.3390/molecules23123356 (2018).
187 Wu, S. et al. Modulation of Gut Microbiota by Lonicera caerulea L. Berry Polyphenols in a Mouse Model of Fatty Liver Induced by High Fat Diet. Molecules 23, doi:10.3390/molecules23123213 (2018).
188 Ishii, C. et al. A Metabologenomic Approach Reveals Changes in the Intestinal Environment of Mice Fed on American Diet. Int J Mol Sci 19, doi:10.3390/ijms19124079 (2018).
189 Arora, T. et al. Microbial fermentation of flaxseed fibers modulates the transcriptome of GPR41-expressing enteroendocrine cells and protects mice against diet-induced obesity. Am J Physiol Endocrinol Metab 316, E453-E463, doi:10.1152/ajpendo.00391.2018 (2019).
190 Xie, M. et al. Effects of Dicaffeoylquinic Acids from Ilex kudingcha on Lipid Metabolism and Intestinal Microbiota in High-Fat-Diet-Fed Mice. J Agric Food Chem 67, 171-183, doi:10.1021/acs.jafc.8b05444 (2019).
191 Townsend, G. E., 2nd et al. Dietary sugar silences a colonization factor in a mammalian gut symbiont. Proc Natl Acad Sci U S A 116, 233-238, doi:10.1073/pnas.1813780115 (2019).

192 Jia, N. et al. Amelioration of hepatic steatosis is associated with modulation of gut microbiota and suppression of hepatic miR-34a in Gynostemma pentaphylla (Thunb.) Makino treated mice. Nutr Metab (Lond) 15, 86, doi:10.1186/s12986-018-0323-6 (2018).
193 Xie, Y., Zhou, G., Wang, C., Xu, X. & Li, C. Specific Microbiota Dynamically Regulate the Bidirectional Gut-Brain Axis Communications in Mice Fed Meat Protein Diets. J Agric Food Chem 67, 1003-1017, doi:10.1021/acs.jafc.8b05654 (2019).
194 An, Y. et al. Cordycepin reduces weight through regulating gut microbiota in high-fat diet-induced obese rats. Lipids Health Dis 17, 276, doi:10.1186/s12944-018-0910-6 (2018).
195 Lindheim, L. et al. Reproductive and Behavior Dysfunction Induced by Maternal Androgen Exposure and Obesity Is Likely Not Gut Microbiome-Mediated. J Endocr Soc 2, 1363-1380, doi:10.1210/js.2018-00266 (2018).
196 Shi, C. et al. High fat diet exacerbates intestinal barrier dysfunction and changes gut microbiota in intestinal-specific ACF7 knockout mice. Biomed Pharmacother 110, 537-545, doi:10.1016/j.biopha.2018.11.100 (2019).
197 Sgritta, M. et al. Mechanisms Underlying Microbial-Mediated Changes in Social Behavior in Mouse Models of Autism Spectrum Disorder. Neuron 101, 246-259 e246, doi:10.1016/j.neuron.2018.11.018 (2019).
198 Liso, M. et al. A Bronze-Tomato Enriched Diet Affects the Intestinal Microbiome under Homeostatic and Inflammatory Conditions. Nutrients 10, doi:10.3390/nu10121862 (2018).
199 Du, Y. W. et al. Effects of Taste Signaling Protein Abolishment on Gut Inflammation in an Inflammatory Bowel Disease Mouse Model. J Vis Exp, doi:10.3791/58668 (2018).
200 Li, S. et al. Fucosylated chondroitin sulfate from Isostichopus badionotus alleviates metabolic syndromes and gut microbiota dysbiosis induced by high-fat and high-fructose diet. Int J Biol Macromol 124, 377-388, doi:10.1016/j.ijbiomac.2018.11.167 (2019).
201 Hendrikx, T. et al. Bacteria engineered to produce IL-22 in intestine induce expression of REG3G to reduce ethanol-induced liver disease in mice. Gut, doi:10.1136/gutjnl-2018-317232 (2018).
202 Albaugh, V. L. et al. Role of Bile Acids and GLP-1 in Mediating the Metabolic Improvements of Bariatric Surgery. Gastroenterology 156, 1041-1051 e1044, doi:10.1053/j.gastro.2018.11.017 (2019).
203 Suwal, S. et al. The Probiotic Effectiveness in Preventing Experimental Colitis Is Correlated With Host Gut Microbiota. Front Microbiol 9, 2675, doi:10.3389/fmicb.2018.02675 (2018).
204 Graf, B. L. et al. Physicochemical differences between malanga (Xanthosoma sagittifolium) and potato (Solanum tuberosum) tubers are associated with differential effects on the gut microbiome. J Funct Foods 45, 268-276, doi:10.1016/j.jff.2018.04.032 (2018).
205 Zhai, X., Lin, D., Zhao, Y., Li, W. & Yang, X. Effects of Dietary Fiber Supplementation on Fatty Acid Metabolism and Intestinal Microbiota Diversity in C57BL/6J Mice Fed with a High-Fat Diet. J Agric Food Chem 66, 12706-12718, doi:10.1021/acs.jafc.8b05036 (2018).
206 Heeney, D. D. et al. Lactobacillus plantarum bacteriocin is associated with intestinal and systemic improvements in diet-induced obese mice and maintains epithelial barrier integrity in vitro. Gut Microbes 10, 382-397, doi:10.1080/19490976.2018.1534513 (2019).
207 Wankhade, U. D. et al. Maternal High-Fat Diet Programs Offspring Liver Steatosis in a Sexually Dimorphic Manner in Association with Changes in Gut Microbial Ecology in Mice. Sci Rep 8, 16502, doi:10.1038/s41598-018-34453-0 (2018).
208 Patrone, V. et al. Differential effects of coconut versus soy oil on gut microbiota composition and predicted metabolic function in adult mice. BMC Genomics 19, 808, doi:10.1186/s12864-018-5202-z (2018).
209 Koh, A. et al. Microbially Produced Imidazole Propionate Impairs Insulin Signaling through mTORC1. Cell 175, 947-961 e917, doi:10.1016/j.cell.2018.09.055 (2018).

210 Sun, L. et al. Gut microbiota and intestinal FXR mediate the clinical benefits of metformin. Nat Med 24, 1919-1929, doi:10.1038/s41591-018-0222-4 (2018).
211 Kasahara, K. et al. Interactions between Roseburia intestinalis and diet modulate atherogenesis in a murine model. Nat Microbiol 3, 1461-1471, doi:10.1038/s41564-018-0272-x (2018).
212 Nagano, T., Katase, M. & Tsumura, K. Inhibitory effects of dietary soy isoflavone and gut microbiota on contact hypersensitivity in mice. Food Chem 272, 33-38, doi:10.1016/j.foodchem.2018.08.043 (2019).
213 Guss, J. D. et al. The effects of metabolic syndrome, obesity, and the gut microbiome on load-induced osteoarthritis. Osteoarthritis Cartilage 27, 129-139, doi:10.1016/j.joca.2018.07.020 (2019).
214 Ye, J. et al. Butyrate Protects Mice Against Methionine-Choline-Deficient Diet-Induced Non-alcoholic Steatohepatitis by Improving Gut Barrier Function, Attenuating Inflammation and Reducing Endotoxin Levels. Front Microbiol 9, 1967, doi:10.3389/fmicb.2018.01967 (2018).
215 Tazi, A. et al. Disentangling Host-Microbiota Regulation of Lipid Secretion by Enterocytes: Insights from Commensals Lactobacillus paracasei and Escherichia coli. Mbio 9, doi:10.1128/mBio.01493-18 (2018).
216 Lieber, A. D. et al. Loss of HDAC6 alters gut microbiota and worsens obesity. Faseb J 33, 1098-1109, doi:10.1096/fj.201701586R (2019).
217 Wu, T. R. et al. Gut commensal Parabacteroides goldsteinii plays a predominant role in the anti-obesity effects of polysaccharides isolated from Hirsutella sinensis. Gut 68, 248-262, doi:10.1136/gutjnl-2017-315458 (2019).
218 Zheng, J. et al. N-Acetylcysteine alleviates gut dysbiosis and glucose metabolic disorder in high-fat diet-fed mice. J Diabetes 11, 32-45, doi:10.1111/1753-0407.12795 (2019).
219 Chen, Y. T. et al. A combination of Lactobacillus mali APS1 and dieting improved the efficacy of obesity treatment via manipulating gut microbiome in mice. Sci Rep 8, 6153, doi:10.1038/s41598-018-23844-y (2018).
220 Hoving, L. R. et al. Dietary Mannan Oligosaccharides Modulate Gut Microbiota, Increase Fecal Bile Acid Excretion, and Decrease Plasma Cholesterol and Atherosclerosis Development. Mol Nutr Food Res 62, e1700942, doi:10.1002/mnfr.201700942 (2018).
221 Hugenholtz, F. et al. Metatranscriptome analysis of the microbial fermentation of dietary milk proteins in the murine gut. PLoS One 13, e0194066, doi:10.1371/journal.pone.0194066 (2018).
222 den Hartigh, L. J. et al. Obese Mice Losing Weight Due to trans-10,cis-12 Conjugated Linoleic Acid Supplementation or Food Restriction Harbor Distinct Gut Microbiota. J Nutr 148, 562-572, doi:10.1093/jn/nxy011 (2018).
223 Hung, T. V. & Suzuki, T. Dietary Fermentable Fibers Attenuate Chronic Kidney Disease in Mice by Protecting the Intestinal Barrier. J Nutr 148, 552-561, doi:10.1093/jn/nxy008 (2018).
224 Pace, F. et al. Helminth infection in mice improves insulin sensitivity via modulation of gut microbiota and fatty acid metabolism. Pharmacol Res 132, 33-46, doi:10.1016/j.phrs.2018.04.008 (2018).
225 Martinez-Guryn, K. et al. Small Intestine Microbiota Regulate Host Digestive and Absorptive Adaptive Responses to Dietary Lipids. Cell Host Microbe 23, 458-469 e455, doi:10.1016/j.chom.2018.03.011 (2018).
226 Wang, K. et al. Structural Modification of Natural Product Ganomycin I Leading to Discovery of a alpha-Glucosidase and HMG-CoA Reductase Dual Inhibitor Improving Obesity and Metabolic Dysfunction in Vivo. J Med Chem 61, 3609-3625, doi:10.1021/acs.jmedchem.8b00107 (2018).
227 Zheng, X. et al. Food withdrawal alters the gut microbiota and metabolome in mice. Faseb J 32, 4878-4888, doi:10.1096/fj.201700614R (2018).
228 Jin, G. et al. Proanthocyanidin-Rich Grape Seed Extract Modulates Intestinal Microbiota in Ovariectomized Mice. J Food Sci 83, 1149-1152, doi:10.1111/1750-3841.14098 (2018).
229 Miranda, P. M. et al. High salt diet exacerbates colitis in mice by decreasing Lactobacillus levels and butyrate production. Microbiome 6, 57, doi:10.1186/s40168-018-0433-4 (2018).

230 Pan, F. et al. Predominant gut Lactobacillus murinus strain mediates anti-inflammaging effects in calorie-restricted mice. Microbiome 6, 54, doi:10.1186/s40168-018-0440-5 (2018).
231 Sanguinetti, E. et al. Microbiome-metabolome signatures in mice genetically prone to develop dementia, fed a normal or fatty diet. Sci Rep 8, 4907, doi:10.1038/s41598-018-23261-1 (2018).
232 Villamil, S. I., Huerlimann, R., Morianos, C., Sarnyai, Z. & Maes, G. E. Adverse effect of early-life high-fat/high-carbohydrate ("Western") diet on bacterial community in the distal bowel of mice. Nutr Res 50, 25-36, doi:10.1016/j.nutres.2017.11.008 (2018).
233 Fujisaka, S. et al. Diet, Genetics, and the Gut Microbiome Drive Dynamic Changes in Plasma Metabolites. Cell Rep 22, 3072-3086, doi:10.1016/j.celrep.2018.02.060 (2018).
234 Kato, T. et al. ChREBP-Knockout Mice Show Sucrose Intolerance and Fructose Malabsorption. Nutrients 10, doi:10.3390/nu10030340 (2018).
235 Wang, D. et al. In utero and lactational exposure to BDE-47 promotes obesity development in mouse offspring fed a high-fat diet: impaired lipid metabolism and intestinal dysbiosis. Arch Toxicol 92, 1847-1860, doi:10.1007/s00204-018-2177-0 (2018).
236 An, J. et al. Physiological mechanisms of sustained fumagillin-induced weight loss. JCI Insight 3, doi:10.1172/jci.insight.99453 (2018).
237 Chen, G. et al. Fuzhuan Brick Tea Polysaccharides Attenuate Metabolic Syndrome in High-Fat Diet Induced Mice in Association with Modulation in the Gut Microbiota. J Agric Food Chem 66, 2783-2795, doi:10.1021/acs.jafc.8b00296 (2018).
238 Friedman, J. E. et al. Pyrroloquinoline quinone prevents developmental programming of microbial dysbiosis and macrophage polarization to attenuate liver fibrosis in offspring of obese mice. Hepatol Commun 2, 313-328, doi:10.1002/hep4.1139 (2018).
239 Janssen, A. W. F. et al. Loss of angiopoietin-like 4 (ANGPTL4) in mice with diet-induced obesity uncouples visceral obesity from glucose intolerance partly via the gut microbiota. Diabetologia 61, 1447-1458, doi:10.1007/s00125-018-4583-5 (2018).
240 Rodriguez-Palacios, A. et al. 'Cyclical Bias' in Microbiome Research Revealed by A Portable Germ-Free Housing System Using Nested Isolation. Sci Rep 8, 3801, doi:10.1038/s41598-018-20742-1 (2018).
241 Wu, J. et al. Salmonella-Mediated Inflammation Eliminates Competitors for Fructose-Asparagine in the Gut. Infect Immun 86, doi:10.1128/IAI.00945-17 (2018).
242 Zhou, X. L., Yan, B. B., Xiao, Y., Zhou, Y. M. & Liu, T. Y. Tartary buckwheat protein prevented dyslipidemia in high-fat diet-fed mice associated with gut microbiota changes. Food Chem Toxicol 119, 296-301, doi:10.1016/j.fct.2018.02.052 (2018).
243 Chelakkot, C. et al. Akkermansia muciniphila-derived extracellular vesicles influence gut permeability through the regulation of tight junctions. Exp Mol Med 50, e450, doi:10.1038/emm.2017.282 (2018).
244 Ciciliot, S. et al. Interplay between gut microbiota and p66Shc affects obesity-associated insulin resistance. Faseb J 32, 4004-4015, doi:10.1096/fj.201701409R (2018).
245 Zheng, J. et al. Chitin Oligosaccharide Modulates Gut Microbiota and Attenuates High-Fat-Diet-Induced Metabolic Syndrome in Mice. Mar Drugs 16, doi:10.3390/md16020066 (2018).
246 Wang, H., Hong, T., Li, N., Zang, B. & Wu, X. Soluble dietary fiber improves energy homeostasis in obese mice by remodeling the gut microbiota. Biochem Biophys Res Commun 498, 146-151, doi:10.1016/j.bbrc.2018.02.017 (2018).
247 Mischke, M. et al. Specific synbiotics in early life protect against diet-induced obesity in adult mice. Diabetes Obes Metab 20, 1408-1418, doi:10.1111/dom.13240 (2018).
248 Wang, L. et al. Green Tea Polyphenols Modulate Colonic Microbiota Diversity and Lipid Metabolism in High-Fat Diet Treated HFA Mice. J Food Sci 83, 864-873, doi:10.1111/1750-3841.14058 (2018).
249 Lee, E. S. et al. Effects of bentonite Bgp35b-p on the gut microbiota of mice fed a high-fat diet. J Sci Food Agric 98, 4369-4373, doi:10.1002/jsfa.8934 (2018).

250 Wang, X. et al. Diabetic cognitive dysfunction is associated with increased bile acids in liver and activation of bile acid signaling in intestine. Toxicol Lett 287, 10-22, doi:10.1016/j.toxlet.2018.01.006 (2018).
251 Libbey, J. E. et al. Variations in diet cause alterations in microbiota and metabolites that follow changes in disease severity in a multiple sclerosis model. Benef Microbes 9, 495-513, doi:10.3920/BM2017.0116 (2018).
252 Chen, L. et al. Chemoprevention of colorectal cancer by black raspberry anthocyanins involved the modulation of gut microbiota and SFRP2 demethylation. Carcinogenesis 39, 471-481, doi:10.1093/carcin/bgy009 (2018).
253 Battson, M. L. et al. Suppression of gut dysbiosis reverses Western diet-induced vascular dysfunction. Am J Physiol Endocrinol Metab 314, E468-E477, doi:10.1152/ajpendo.00187.2017 (2018).
254 Chen, G. et al. Kudingcha and Fuzhuan Brick Tea Prevent Obesity and Modulate Gut Microbiota in High-Fat Diet Fed Mice. Mol Nutr Food Res 62, e1700485, doi:10.1002/mnfr.201700485 (2018).
255 Carvalho, G. et al. Impact of Trans-Fats on Heat-Shock Protein Expression and the Gut Microbiota Profile of Mice. J Food Sci 83, 489-498, doi:10.1111/1750-3841.13997 (2018).
256 Zhang, Y. et al. Dietary Factors Modulate Colonic Tumorigenesis Through the Interaction of Gut Microbiota and Host Chloride Channels. Mol Nutr Food Res 62, doi:10.1002/mnfr.201700554 (2018).
257 Connor, K. L. et al. Maternal metabolic, immune, and microbial systems in late pregnancy vary with malnutrition in mice. Biol Reprod 98, 579-592, doi:10.1093/biolre/ioy002 (2018).
258 Collins, J. et al. Dietary trehalose enhances virulence of epidemic Clostridium difficile. Nature 553, 291-294, doi:10.1038/nature25178 (2018).
259 Prieto, I. et al. Influence of a diet enriched with virgin olive oil or butter on mouse gut microbiota and its correlation to physiological and biochemical parameters related to metabolic syndrome. PLoS One 13, e0190368, doi:10.1371/journal.pone.0190368 (2018).
260 Schroeder, B. O. et al. Bifidobacteria or Fiber Protects against Diet-Induced Microbiota-Mediated Colonic Mucus Deterioration. Cell Host Microbe 23, 27-40 e27, doi:10.1016/j.chom.2017.11.004 (2018).
261 Zou, J. et al. Fiber-Mediated Nourishment of Gut Microbiota Protects against Diet-Induced Obesity by Restoring IL-22-Mediated Colonic Health. Cell Host Microbe 23, 41-53 e44, doi:10.1016/j.chom.2017.11.003 (2018).
262 Li, C. C. et al. Tomato Powder Inhibits Hepatic Steatosis and Inflammation Potentially Through Restoring SIRT1 Activity and Adiponectin Function Independent of Carotenoid Cleavage Enzymes in Mice. Mol Nutr Food Res 62, e1700738, doi:10.1002/mnfr.201700738 (2018).
263 Zeng, H., Ishaq, S. L., Liu, Z. & Bukowski, M. R. Colonic aberrant crypt formation accompanies an increase of opportunistic pathogenic bacteria in C57BL/6 mice fed a high-fat diet. J Nutr Biochem 54, 18-27, doi:10.1016/j.jnutbio.2017.11.001 (2018).
264 Zhao, C. et al. Regulatory Efficacy of Brown Seaweed Lessonia nigrescens Extract on the Gene Expression Profile and Intestinal Microflora in Type 2 Diabetic Mice. Mol Nutr Food Res 62, doi:10.1002/mnfr.201700730 (2018).
265 Zhu, L. et al. Berberine treatment increases Akkermansia in the gut and improves high-fat diet-induced atherosclerosis in Apoe(-/-) mice. Atherosclerosis 268, 117-126, doi:10.1016/j.atherosclerosis.2017.11.023 (2018).
266 Villa, C. R., Taibi, A., Chen, J., Ward, W. E. & Comelli, E. M. Colonic Bacteroides are positively associated with trabecular bone structure and programmed by maternal vitamin D in male but not female offspring in an obesogenic environment. Int J Obes (Lond) 42, 696-703, doi:10.1038/ijo.2017.294 (2018).

267 Laverdure, R., Mezouari, A., Carson, M. A., Basiliko, N. & Gagnon, J. A role for methanogens and methane in the regulation of GLP-1. Endocrinol Diabetes Metab 1, e00006, doi:10.1002/edm2.6 (2018).
268 Llewellyn, S. R. et al. Interactions Between Diet and the Intestinal Microbiota Alter Intestinal Permeability and Colitis Severity in Mice. Gastroenterology 154, 1037-1046 e1032, doi:10.1053/j.gastro.2017.11.030 (2018).
269 Radulovic, K. et al. A dietary flavone confers communicable protection against colitis through NLRP6 signaling independently of inflammasome activation. Mucosal Immunol 11, 811-819, doi:10.1038/mi.2017.87 (2018).
270 Jiang, Z. et al. Antimicrobial Emulsifier-Glycerol Monolaurate Induces Metabolic Syndrome, Gut Microbiota Dysbiosis, and Systemic Low-Grade Inflammation in Low-Fat Diet Fed Mice. Mol Nutr Food Res 62, doi:10.1002/mnfr.201700547 (2018).
271 Singh, D. P. et al. Co-supplementation of isomalto-oligosaccharides potentiates metabolic health benefits of polyphenol-rich cranberry extract in high fat diet-fed mice via enhanced gut butyrate production. Eur J Nutr 57, 2897-2911, doi:10.1007/s00394-017-1561-5 (2018).
272 Lu, C. et al. Microbial diversity and composition in different gut locations of hyperlipidemic mice receiving krill oil. Appl Microbiol Biotechnol 102, 355-366, doi:10.1007/s00253-017-8601-1 (2018).
273 Chen, S., Li, X., Liu, L., Liu, C. & Han, X. Ophiopogonin D alleviates high-fat diet-induced metabolic syndrome and changes the structure of gut microbiota in mice. Faseb J 32, 1139-1153, doi:10.1096/fj.201700741RR (2018).
274 Gao, X. et al. Polyphenol- and Caffeine-Rich Postfermented Pu-erh Tea Improves Diet-Induced Metabolic Syndrome by Remodeling Intestinal Homeostasis in Mice. Infect Immun 86, doi:10.1128/IAI.00601-17 (2018).
275 Shtriker, M. G. et al. Fenugreek galactomannan and citrus pectin improve several parameters associated with glucose metabolism and modulate gut microbiota in mice. Nutrition 46, 134-142 e133, doi:10.1016/j.nut.2017.07.012 (2018).
276 Henning, S. M. et al. Decaffeinated green and black tea polyphenols decrease weight gain and alter microbiome populations and function in diet-induced obese mice. Eur J Nutr 57, 2759-2769, doi:10.1007/s00394-017-1542-8 (2018).
277 Van Hul, M. et al. Reduced obesity, diabetes, and steatosis upon cinnamon and grape pomace are associated with changes in gut microbiota and markers of gut barrier. Am J Physiol Endocrinol Metab 314, E334-E352, doi:10.1152/ajpendo.00107.2017 (2018).
278 Sarma, S. M. et al. Finger millet arabinoxylan protects mice from high-fat diet induced lipid derangements, inflammation, endotoxemia and gut bacterial dysbiosis. Int J Biol Macromol 106, 994-1003, doi:10.1016/j.ijbiomac.2017.08.100 (2018).
279 Garcia-Mazcorro, J. F., Mills, D. A., Murphy, K. & Noratto, G. Effect of barley supplementation on the fecal microbiota, caecal biochemistry, and key biomarkers of obesity and inflammation in obese db/db mice. Eur J Nutr 57, 2513-2528, doi:10.1007/s00394-017-1523-y (2018).
280 Wang, C. Z. et al. Role of intestinal microbiome in American ginseng-mediated colon cancer prevention in high fat diet-fed AOM/DSS mice [corrected]. Clin Transl Oncol 20, 302-312, doi:10.1007/s12094-017-1717-z (2018).
281 Lamas, B. et al. Card9 mediates susceptibility to intestinal pathogens through microbiota modulation and control of bacterial virulence. Gut 67, 1836-1844, doi:10.1136/gutjnl-2017-314195 (2018).
282 Chang, C. J. et al. Antrodia cinnamomea reduces obesity and modulates the gut microbiota in high-fat diet-fed mice. Int J Obes (Lond) 42, 231-243, doi:10.1038/ijo.2017.149 (2018).
283 Catry, E. et al. Targeting the gut microbiota with inulin-type fructans: preclinical demonstration of a novel approach in the management of endothelial dysfunction. Gut 67, 271-283, doi:10.1136/gutjnl-2016-313316 (2018).

284 Balakumar, M. et al. Improvement in glucose tolerance and insulin sensitivity by probiotic strains of Indian gut origin in high-fat diet-fed C57BL/6J mice. Eur J Nutr 57, 279-295, doi:10.1007/s00394-016-1317-7 (2018).
